# Derepression of the Iroquois Homeodomain Transcription Factor Gene *IRX3* Confers Differentiation Block in Acute Leukemia

**DOI:** 10.1016/j.celrep.2017.12.063

**Published:** 2018-01-29

**Authors:** Tim D.D. Somerville, Fabrizio Simeoni, John A. Chadwick, Emma L. Williams, Gary J. Spencer, Katalin Boros, Christopher Wirth, Eleni Tholouli, Richard J. Byers, Tim C.P. Somervaille

**Affiliations:** 1 Leukaemia Biology Laboratory, Cancer Research UK Manchester Institute, The University of Manchester, Manchester M20 4BX, UK; 2 Department of Histopathology, Manchester University NHS Foundation Trust, Manchester M13 9WL, UK; 3 Applied Computational Biology and Bioinformatics Group, Cancer Research UK Manchester Institute, The University of Manchester, Manchester M20 4BX, UK; 4 Department of Haematology, Manchester University NHS Foundation Trust, Manchester M13 9WL, UK

**Keywords:** IRX3, HOXA9, acute myeloid leukemia, acute lymphoblastic leukemia

## Abstract

The Iroquois homeodomain transcription factor gene *IRX3* is expressed in the developing nervous system, limb buds, and heart, and transcript levels specify obesity risk in humans. We now report a functional role for *IRX3* in human acute leukemia. Although transcript levels are very low in normal human bone marrow cells, high *IRX3* expression is found in ∼30% of patients with acute myeloid leukemia (AML), ∼50% with T-acute lymphoblastic leukemia, and ∼20% with B-acute lymphoblastic leukemia, frequently in association with high-level HOXA gene expression. Expression of *IRX3* alone was sufficient to immortalize hematopoietic stem and progenitor cells (HSPCs) in myeloid culture and induce lymphoid leukemias *in vivo*. *IRX3* knockdown induced terminal differentiation of AML cells. Combined *IRX3* and *Hoxa9* expression in murine HSPCs impeded normal T-progenitor differentiation in lymphoid culture and substantially enhanced the morphologic and phenotypic differentiation block of AML in myeloid leukemia transplantation experiments through suppression of a terminal myelomonocytic program. Likewise, in cases of primary human AML, high *IRX3* expression is strongly associated with reduced myelomonocytic differentiation. Thus, tissue-inappropriate derepression of *IRX3* contributes significantly to the block in differentiation, which is the pathognomonic feature of human acute leukemias.

## Highlights

• *IRX3* is derepressed in ∼30% of AML, ∼50% of T-ALL, and 20% of B-ALL
• IRX3 suppresses a mature myelomonocytic differentiation program in AML
• IRX3 impairs T-lineage differentiation, expanding an early T-progenitor population
• IRX3 contributes to the differentiation block that is characteristic of the disease

## Graphical Abstract

**Figure:**
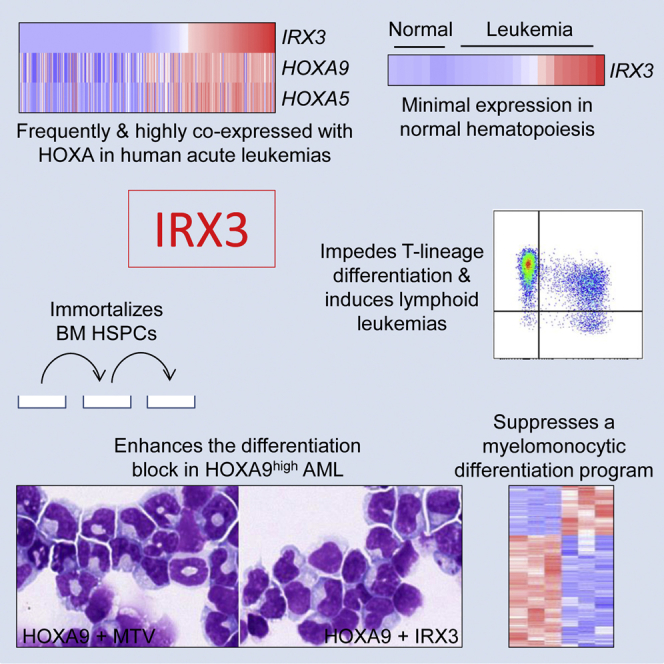

## Teaser

Somerville et al. report that the Iroquois homeodomain transcription factor gene *IRX3* is frequently derepressed in human acute leukemias of multiple lineages and contributes to the differentiation block, which is the pathognomonic feature of the disease.

## Introduction

The cardinal pathologic feature of the acute leukemias is a block to normal blood cell differentiation that results in an accumulation in the bone marrow (BM) of incompletely differentiated blast cells and failure of normal hematopoiesis (Wiseman et al., 2014). Although the spectrum of mutations associated with these diseases is now well established, the biologic basis of how mutations interact with one another to establish the pathognomonic differentiation block is less well understood. We recently reported that the Forkhead box transcription factor FOXC1 is misexpressed in approximately 20% of patients with acute myeloid leukemia (AML), in particular in those cases exhibiting high HOXA/B gene expression (Somerville et al., 2015). *FOXC1* is neither required for nor expressed in normal hematopoietic cells but is essential for normal development of mesenchymal tissues such as the skeleton, heart, and eye and for the normal function of BM niche cells (Omatsu et al., 2014). Its misexpression in leukemic hematopoiesis contributes to a block in differentiation along both monocytic and B cell lineages and is associated with inferior survival. Given the lack of one-to-one correlation with any specific mutation, tissue-inappropriate derepression of *FOXC1* is paradigmatic for a non-mutational mechanism contributing to cellular transformation in myeloid malignancy.

Whether tissue-inappropriate misexpression of other transcription factors contributes to the differentiation block of leukemia is not known. One candidate is the Iroquois homeobox transcription factor gene *IRX3*, which, like *FOXC1*, is expressed in a significant proportion of patients with AML (Somerville et al., 2015) but minimally expressed in both normal hematopoietic stem and progenitor cells (HSPCs) and mature blood cells (ENCODE data; Zhou et al., 2011). IRX3 is a member of the three-amino-acid-loop-extension (TALE) superfamily of homeodomain transcription factors, which also includes MEIS1 and PBX1 (Mukherjee and Bürglin, 2007). In embryogenesis, it is strongly expressed in the developing nervous system, as well as in mesoderm-derived tissues such as the limb buds, kidney, and heart (Houweling et al., 2001). Of note, the developmental expression pattern of the *Irx3* paralog *Irx5*, which sits in the same 2MB topologically associating domain, is strikingly similar (Cohen et al., 2000, Claussnitzer et al., 2015). These genes exhibit functional redundancy because although *Irx3*-null and *Irx5*-null mice are viable and fertile, mice lacking both genes die in utero because of severe cardiac and skeletal defects (Zhang et al., 2011, Gaborit et al., 2012, Li et al., 2014, Smemo et al., 2014). Interestingly, non-coding variation in an enhancer region 500 kb downstream of *IRX3* provides the strongest genetic association with risk for human obesity. Pertinent to this, adult *Irx3*-null mice display a 25%–30% reduction in body weight due to loss of fat mass, increased basal metabolic rate, and browning of white adipose tissue, attributable to loss of hypothalamic (Smemo et al., 2014) or preadipocyte (Claussnitzer et al., 2015) *Irx3* expression. The rs1421085 single-nucleotide variant present in the obesity risk region dictates the extent of local recruitment of ARID5B to the *IRX3* enhancer, with consequent regulation of *IRX3* expression (Claussnitzer et al., 2015).

Whether *IRX3* has a role in human malignancy is unclear. One study reported that *IRX3* is strongly expressed in colorectal adenomas in comparison with normal mucosa and negatively regulates TGF-β signaling in colorectal cancer cell lines (Martorell et al., 2014). However, little else is known. Given this, and the observation that *IRX3* is highly expressed in a subset of AML patients, we evaluated whether *IRX3* has a functional role in acute leukemia.

## Results

### *IRX3* Is Frequently Co-expressed with HOX Genes in Human AML

**Figure 1:**
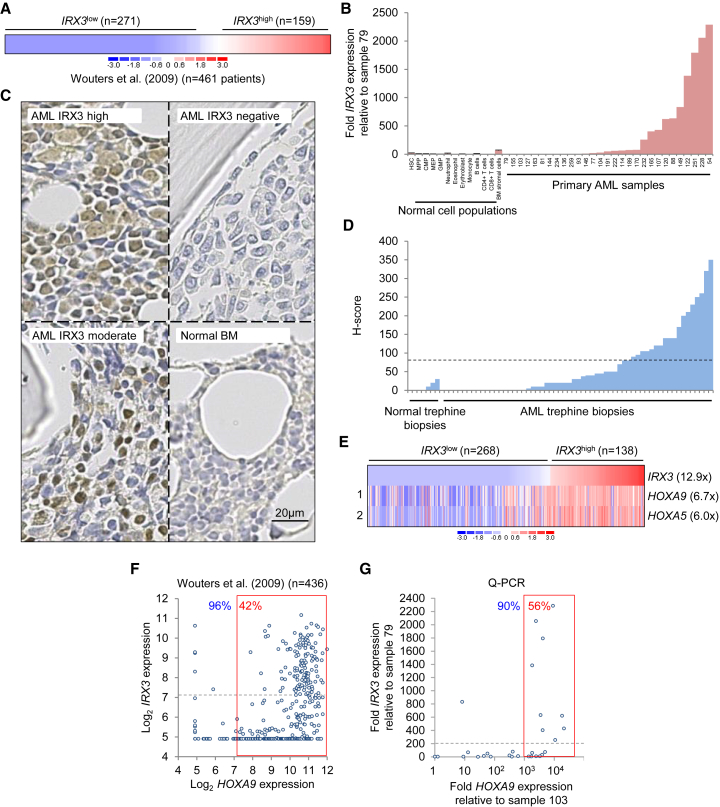
*IRX3* Expression in Human AML (A) *IRX3* expression in bulk human AML samples. (B) Relative *IRX3* expression in bulk primary human AML samples (n = 29) and normal human BM cell populations (n = 4 separate individuals per cell type; error bars = SEM). BM, bone marrow; CMP, common myeloid progenitor; Eosin, eosinophils; EryB, erythroblast; GMP, granulocyte-macrophage progenitor; HSC, hematopoietic stem cell (CD34^+^38^−^90^+^45RA^−^Lin^−^); MEP, megakaryocyte-erythrocyte progenitor; Mono, monocytes; MPP, multipotent progenitor; Neut, neutrophils. AML sample numbers refer to Biobank identifier. (C) Representative images of IRX3 immune staining of human BM trephine biopsies. (D) H scores for IRX3 immune staining. Dashed line indicates cutoff value for moderate/strong immune staining. (E) Heatmap shows *IRX3*^high^ versus *IRX3*^low^ AML cases (excluding APML) (Wouters et al., 2009) and the most differentially expressed transcription factor genes with mean fold change. (F and G) Scatterplots show *IRX3* versus *HOXA9* expression in primary AML samples as determined by (F) array values or (G) qPCR. For scatterplots, percentages in blue text indicate proportion of *IRX3*^high^ samples exhibiting high *HOXA9* expression. Percentages in red text indicate the proportion of *HOXA9*^high^ samples (in the red boxes) additionally exhibiting high *IRX3* expression (above the dotted gray line). See also Figure S1 and Tables S1–S4.

To ascertain the frequency and extent of *IRX3* expression in AML and in flow-sorted normal human BM cell populations, we performed both qPCR and analyses of published datasets. In a Dutch cohort of AML patients treated intensively with anthracycline-based chemotherapy on the Hemato-Oncologie voor Volwassenen Nederland (HOVON) protocols, *IRX3* transcripts were detected at high level (i.e., with a probeset [229638_at] value of log_2_ > 7.1, approximating to a value among the top 25% of array probeset values) in 159 of 461 bulk presentation samples (34%) (Wouters et al., 2009) (Figure 1A). Likewise, in The Cancer Genome Atlas Research Network series, 49 of 163 cases (30%) expressed *IRX3* at high level (Cerami et al., 2012, Ley et al., 2013) (Figure S1A). In flow-sorted populations of AML cells with immature immunophenotypes, *IRX3* was highly expressed (i.e., among the top 25% of array probeset values) in 33% (Saito et al., 2010) (Figure S1B), 58% (Kikushige et al., 2010) (Figure S1C), and 19% (Goardon et al., 2011) (Figure S1D) of samples. Concomitant microarray profiling of normal human immunophenotypic HSPCs suggested low or absent *IRX3* expression (Figures S1B–S1D). In keeping with this, our qPCR analyses revealed very low levels of *IRX3* transcripts in all normal BM populations tested, but in 10 of 29 AML samples (34%), *IRX3* expression was increased at least 250-fold over levels observed in the lowest expressing AML sample (Figure 1B; Table S1). *IRX3* transcript levels were higher in normal human CD45^neg^ BM stromal cells than in normal BM cell populations (Figure 1B) but not as high as those observed in many AML samples. Given that expression of *IRX3* and *IRX5* are co-regulated in development, we also performed qPCR for *IRX5* transcripts. We did not detect *IRX5* expression in normal human BM cell populations (data not shown) but did detect low-level expression in 11 of 28 AML samples (39%), typically in cases with high *IRX3* expression (Figure S1E). In the Dutch AML cohort, 44 of 461 cases (10%) expressed *IRX5* at high level (i.e., probeset [210239_at] value of log_2_ > 7.1), and in every case there was also high IRX3 expression (data not shown). Thus, the set of *IRX5*^high^ AML cases is a subset of the group of *IRX3*^high^ cases.

To confirm that IRX3 protein was also expressed in AML, we performed immunohistochemical staining of a trephine biopsy tissue array. The array included 58 samples from patients with AML and 9 samples from patients with non-malignant BM conditions (Table S2). Immune staining was H-scored by blind evaluation. Strong or moderate nuclear immune staining (i.e., H score ≥ 80) was observed in 20 of 58 cases (34%). Weak or absent immune staining was observed in 38 of 58 AML cases (66%) and in all non-malignant cases (Figures 1C and 1D).

High *IRX3* expression in AML was strongly and positively associated with the presence of an *NPM1* mutation or a *FLT3* internal tandem duplication (Tables S3 and S4) (Wouters et al., 2009, Ley et al., 2013). A strong positive association with acute promyelocytic leukemia (APML) was also noted (Table S3). There were weaker positive associations with intermediate cytogenetic risk, normal karyotype and the presence of an *IDH1* mutation, t(6;9), or an *MLL* gene rearrangement. High *IRX3* expression was negatively associated with the presence of chromosome 5 or 7(q) loss, the presence of t(8;21) or inv(16), the presence of mutations in *NRAS*, *TP53*, or *RUNX1*, or a double *CEBPA* mutation and the presence of high *MECOM* expression (Tables S3 and S4). Although detailed genotyping of the tissue array samples was not performed, there was nevertheless a significant association of normal karyotype with strong or moderate IRX3 expression (14 of 17 available karyotypes were normal where strong or moderate nuclear immune staining was present versus 17 of 34 with weak or absent staining, p = 0.035, Fisher’s exact test) (Table S2).

To identify genes co-expressed with *IRX3* in human AML, we next compared *IRX3*^high^ AMLs (probeset 229638_at value of log_2_ > 7.1) with *IRX3*^low^ AMLs (probeset 229638_at value of log_2_ < 6.1) (Wouters et al., 2009) and found *HOXA9* and *HOXA5* to be the most differentially expressed transcription factor genes in the *IRX3*^high^ group whether (data not shown) or not (Figure 1E) cases of APML (which do not express HOX genes) were included. Of the non-APML cases, 133 of 138 *IRX3*^high^ samples (96%) exhibited high *HOXA9* expression (i.e., probeset value of log_2_ > 7.1), and of the *HOXA9*^high^ samples, 133 of 319 (42%) expressed high levels of *IRX3* (Figure 1F). Of the five *IRX3*^high^ *HOXA9*^low^ samples, three expressed one or more alternative HOX genes at significant level (i.e., log_2_ > 7.1), indicating that overall, 98.6% of *IRX3*^high^ non-APML samples exhibited HOX gene co-expression. Similar results were observed in analyses of The Cancer Genome Atlas (TCGA) dataset (Figure S1F) (Ley et al., 2013) and qPCR analysis of our own samples (Figure 1G). Taken together, these data demonstrate that in human AML, the Iroquois homeodomain transcription factor gene *IRX3*, which is minimally expressed in normal hematopoiesis, is often misexpressed in conjunction with high HOX gene expression, as well as in APML. The former association suggests an explanation for the statistically significant associations of *IRX3* expression with the presence of an *NPM1* mutation, *MLL* gene rearrangements, and a t(6;9) because these molecular subtypes are associated with high HOX gene expression.

### IRX3 Promotes Serial Replating of Normal BM Stem and Progenitor Cells

**Figure 2:**
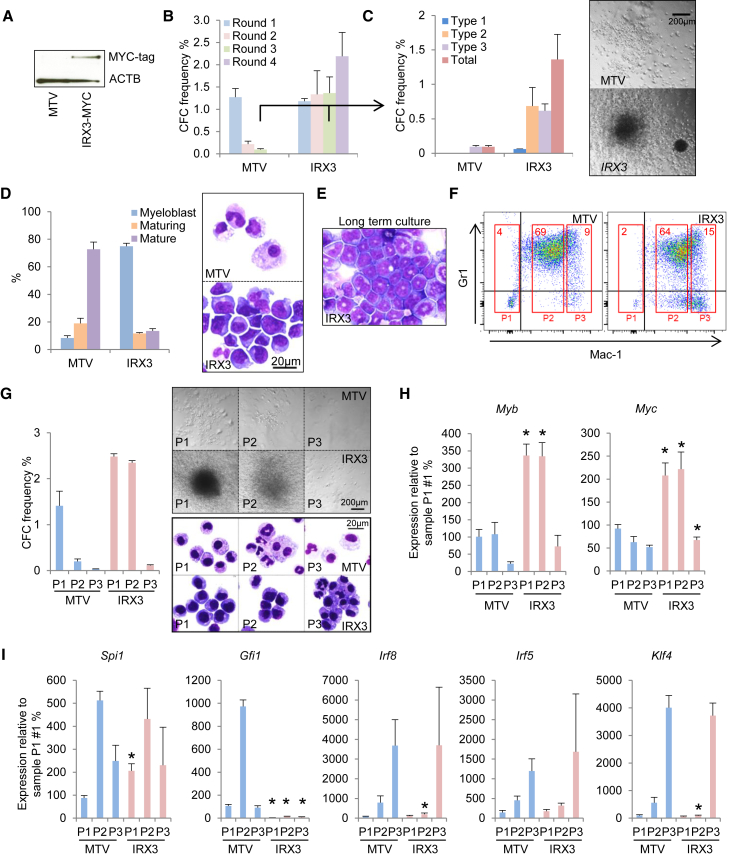
IRX3 Enhances Clonogenic Potential in Normal HSPCs (A) MYC-tagged IRX3 expressed in murine KIT^+^ BM HSPCs 72 hr after retroviral infection. MTV, empty vector. (B) Mean + SEM colony-forming cell (CFC) frequencies during serial replating (n = 3). (C) Bar chart (left) shows mean + SEM colony types in round 3 of culture (n = 3). Type 1, tightly packed colonies, contain blast cells only; type 2, contain mixed population of blasts and mature cells; type 3, contain mature cells only. Image (right) shows representative colonies. (D) Bar chart (left) shows mean + SEM percentage of the indicated cell types in cytospin preparations after 7 days of culture of retrovirally infected murine KIT^+^ BM cells in liquid conditions supporting myeloid development (n = 3). Representative images (right) are shown. (E) Representative image of *IRX3*-expressing mouse BM cells after 5 weeks in liquid culture. (F) Representative flow cytometry plots for the indicated cell surface markers after 7 days in liquid culture; red boxes indicate cell sorting gates. Numbers within boxes indicate percentage of cells with the indicated phenotype. (G) Bar chart (left) shows mean + SEM CFC frequencies for the indicated flow-sorted cell populations enumerated after 5 days (n = 3). Images (right) show representative colonies (top) and cytospins (bottom). (H and I) Bar charts show mean + SEM relative transcript expression for the indicated genes in the indicated cell populations: (H) *Myb* and *Myc*; (I) *Spi1*, *Gfi1*, *Irf8*, *Irf5*, and *Klf4*. ^∗^p ≤ 0.05 for the indicated *IRX3*^+^ population versus the equivalent control population (unpaired t test). See also Figure S1.

To evaluate whether misexpressed *IRX3* has functional consequences, we performed expression experiments in murine KIT^+^ BM HSPCs (Figure 2A). Serial replating assays in conditions supporting myeloid lineage differentiation demonstrated that in comparison with control cells, *IRX3*^+^ cells exhibited enhanced clonogenic activity (Figures 2A and 2B). In the third round of culture, control cells exclusively formed colonies with type 3 morphology (i.e., diffuse colonies containing mature macrophages), whereas *IRX3*^+^ cells in addition formed type 1 (i.e., tightly packed colonies containing blast cells) and type 2 colonies (i.e., mixed colonies containing blast cells and mature cells) (Figure 2C). Reflecting these observations, *IRX3*^+^ BM HSPCs cultured in liquid conditions (with interleukin-3 [IL-3], IL-6, granulocyte-macrophage colony-stimulating factor [GM-CSF], and stem cell factor [SCF]) for 7 days following retroviral transduction exhibited significantly impaired morphologic differentiation in comparison with control cells (Figure 2D) and could readily be grown on for at least 5 weeks (Figure 2E). The great majority of day 7 *IRX3*^+^ and control cells in liquid culture exhibited a Mac1^+^Gr1^+^ immunophenotype, although there was a modest but significant reduction in the percentage of Gr1^+^ cells in the *IRX3*^+^ condition (Figure S1G). Flow-sorting analyses confirmed, as expected, that the clonogenic activity of control day 7 cells was near exclusively confined to cells with a Mac-1^neg^ immunophenotype. In contrast, IRX3^+^ cells with a Mac-1^intermediate^ immunophenotype also exhibited strong clonogenic activity (Figures 2F and 2G). qPCR analysis of genes associated with self-renewal in myeloid leukemia (Somervaille et al., 2009) demonstrated significant upregulation of *Myc* and *Myb* in *IRX3*^+^ populations with significant clonogenic potential (P1 and P2). Furthermore, there was significantly reduced expression in the aberrantly clonogenic *IRX3*^+^ P2 population of transcription factor genes such as *Gfi1*, *Irf8*, and *Klf4*, which are associated with myelomonocytic lineage differentiation (Figure 2I). Thus, expression of *IRX3* in normal BM HSPCs in conditions supporting myeloid lineage differentiation confers a morphologic and functional differentiation block resulting in sustained clonogenic activity, elevated expression of self-renewal genes *Myc* and *Myb*, and reduced expression of myelomonocytic differentiation genes *Gfi1*, *Irf8*, and *Klf4*.

### IRX3 Cooperates with HOXA9 to Enhance Differentiation Block in AML

**Figure 3:**
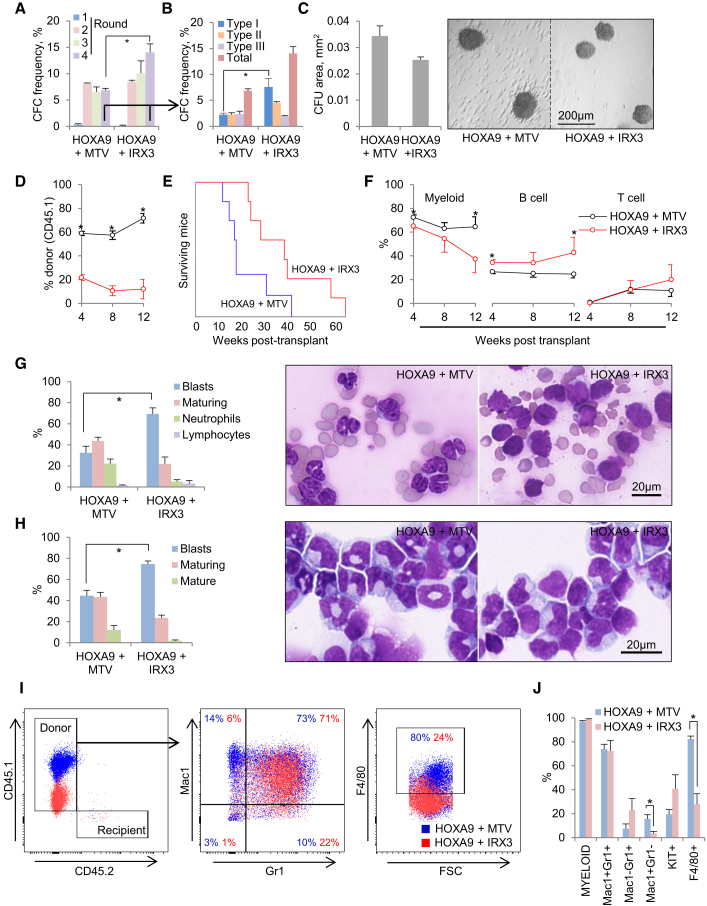
IRX3 Expression Confers an Enhanced Myeloid Differentiation Block in *Hoxa9*^+^ AML (A) Bar chart shows mean + SEM colony-forming cell (CFC) frequencies during serial replating of murine KIT^+^ BM cells co-transduced with the indicated retroviral or control (MTV) expression vectors (n = 3). ^∗^p < 0.05 by unpaired t test. (B) Bar chart shows mean + SEM colony types in round 4 (n = 3). ^∗^p < 0.05 by unpaired t test. (C) Bar chart (left) shows mean + SEM area of type 1 colonies (n = 26–40) from (B) and representative images (right). (D–J) Murine CD45.1^+^ KIT^+^ BM cells were infected in pairwise combination with retroviral vectors, and 96 hr later 10^6^ drug-resistant cells were transplanted into CD45.2^+^ irradiated congenic recipients. (D) Line graph shows mean + SEM percentage donor-derived CD45.1^+^ cells in blood at the indicated times post-transplantation. (E) Survival curves of transplanted mice (n = 7 per cohort). (F) Line graphs show the mean + SEM percentage contribution of donor-derived cells in blood to the indicated lineages at the indicated times post-transplantation. (G) Bar chart (left) shows mean + SEM percentage leucocyte type in blood at death, as determined by morphologic analysis of blood smears (n = 4 or 5 per cohort). Representative images (right) are shown. (H) Bar chart (left) shows mean + SEM percentage cell type in BM at death (n = 5 per cohort). Representative images (left) are shown. (I) Representative flow cytometry plots from (H). (J) Bar chart shows the mean + SEM percentage of donor-derived cells positive for the indicated cell surface markers in BM of leukemic mice, as determined by flow cytometry. ^∗^p < 0.05 by unpaired t test. See also Figures S1–S3.

Given the strong association of *IRX3* and *HOXA9* expression in human AML, we next evaluated the consequences of *IRX3* co-expression in *Hoxa9*-expressing murine BM HSPCs. Cells were infected in pairwise combinations with retroviral vectors expressing *Hoxa9*, *IRX3*, or a control vector (to generate *Hoxa9*/*IRX3* and *Hoxa9/*MTV cells, respectively) and serially replated in semisolid culture. Consistent with *IRX3* co-expression conferring an enhanced differentiation block to *Hoxa9*-immortalized HSPCs, by the fourth round of culture, *Hoxa9*/*IRX3* cells formed significantly more colonies than *Hoxa9/*MTV cells (Figure 3A), and these were significantly more likely to exhibit type 1 blast-like morphology (Figure 3B). Of note, *Hoxa9*/*IRX3* type 1 colonies were on average 25% smaller in cross-sectional area than *Hoxa9/*MTV type 1 colonies (Figure 3C); this was explained by there being a significantly reduced proportion of *Hoxa9*/*IRX3* cells in the SG_2_M phase of the cell cycle (Figure S1H). The immunophenotype of *Hoxa9*/*IRX3* and *Hoxa9/*MTV cells was similar (Figure S1I).

Transplantation of *Hoxa9*-expressing murine BM HSPCs into irradiated syngeneic recipients results in short latency AMLs, which exhibit significant myelomonocytic maturation (Kroon et al., 1998, Somerville et al., 2015). To determine whether co-expression of IRX3 influences leukemia cell differentiation *in vivo*, we transplanted *Hoxa9*/*IRX3* and *Hoxa9*/MTV double-transduced BM HSPCs into irradiated congenic recipients. Consistent with the cell-cycle status of *in vitro* transformed *Hoxa9*/*IRX3* cells, analysis of blood at 4, 8, and 12 weeks post-transplantation demonstrated reduced donor:recipient chimerism in blood in *Hoxa9*/*IRX3* versus *Hoxa9/*MTV recipients (Figure 3D), and *Hoxa9*/*IRX3* recipient mice exhibited delayed onset of donor-derived AML in comparison with *Hoxa9*/MTV recipients (median 125 versus 270 days) (Figure 3E). Mice from both cohorts presented with substantially elevated blood leucocyte counts (Figure S2A), hepatosplenomegaly (Figure S2B), and effacement of BM due to infiltration by leukemia cells (Figures S2C–S2F).

In keeping with a model whereby *IRX3* misexpression *in vivo* blocks myeloid lineage differentiation, evaluation of the lineage composition of donor-derived cells in blood prior to development of AML demonstrated a significant proportional reduction in myeloid and a proportional increase in B-lineage differentiation at 4 and 12 weeks post-transplantation in *Hoxa9*/*IRX3* versus *Hoxa9/*MTV recipients (Figures 3F and S3A). Once mice developed full-fledged AML, this was further confirmed by analysis of blood smear and BM cytospin morphology: *Hoxa9*/*IRX3* recipients developed leukemias exhibiting significantly greater differentiation block in comparison with *Hoxa9*/MTV controls, as demonstrated by the proportion of blast cells being on average twice as high in the former versus the latter and the proportion of more differentiated leukemia cells being approximately half as much (Figures 3G and 3H). Flow cytometry analysis of leukemic BM cells confirmed donor origin and myeloid lineage (Figures 3I, 3J, and S3B) and also revealed a distinctive immunophenotype: *Hoxa9*/*IRX3* AMLs expressed significantly lower levels of both CD45.1 (Figures 3I and S3C) and the monocyte/macrophage differentiation marker F4/80 versus *Hoxa9*/MTV controls. There were also significantly fewer Mac1^+^Gr1^−^ leukemia cells in *Hoxa9*/*IRX3* recipients and, on average, double the percentage of cells positive for the stem and progenitor marker KIT (Figure 3J), although in view of heterogeneous levels of expression, this difference did not reach statistical significance. *Hoxa9*/*IRX3* AML cells induced leukemias in secondarily transplanted recipients (Figure S3D), and high-level *IRX3* expression was readily detected in *Hoxa9/IRX3* AML BM cells (Figure S3E). Interestingly, in contrast to observations *in vitro*, cell-cycle analysis of leukemia cells from BM and spleen of leukemic mice demonstrated no difference in the fraction of cycling cells between the cohorts (Figure S3F), although in both cases, the SG2M fraction was substantially lower in the *in vivo* setting in comparison with the *in vitro* setting (i.e., 10%–20% versus 40%–50%).

Altogether our observations demonstrate that co-expression of *IRX3* modulates the phenotypic consequences of *Hoxa9* expression both *in vitro* and *in vivo*, conferring a significantly enhanced myeloid lineage differentiation block.

### Functional Contribution of IRX3 to Differentiation Block in AML Cells

**Figure 4:**
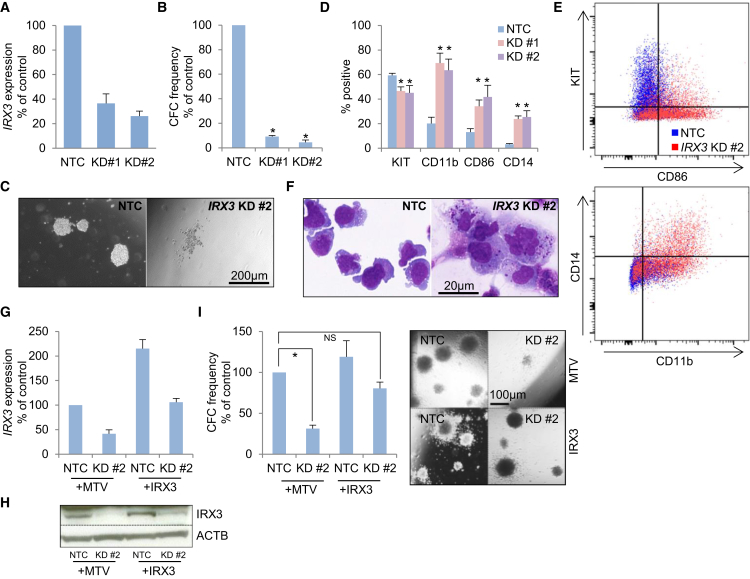
IRX3 Sustains the Differentiation Block and Clonogenic Potential of AML Cells Human THP1 AML cells were infected with lentiviral vectors targeting *IRX3* for KD or a non-targeting control vector (NTC). (A) Bar chart shows mean + SEM relative transcript expression in *IRX3* KD versus control cells (n = 3) after 48 hr. (B) Bar chart shows the mean + SEM colony-forming cell (CFC) frequencies of KD cells relative to control cells enumerated after 10 days in semi-solid culture (n = 4). (C) Representative images of colonies of cells from (B). (D) Bar chart shows mean + SEM percentage of cells positive for the indicated cell surface markers, as determined by flow cytometry analysis 6 days following initiation of KD (n = 4). ^∗^p ≤ 0.05 using one-way ANOVA with Fisher’s least significant difference post hoc analysis for KD conditions versus NTC. (E) Representative flow cytometry plots from (D). (F) Representative images of cytospins of cells from (D). (G) Bar chart shows mean + SEM relative transcript expression in THP1 AML cells expressing either *IRX3* or a control retroviral vector (MTV) in *IRX3* KD#2 cells relative to control cells (n = 3). (H) Western blot shows expression of the indicated proteins in the indicated conditions. (I) Bar chart (left) shows mean + SEM CFC frequencies of THP1 AML cells expressing either *IRX3* or a control retroviral vector (MTV) in *IRX3* KD#2 cells relative to control cells. Colonies were enumerated after 10 days in semi-solid culture (n = 4). Image (right) shows representative colonies. ^∗^p ≤ 0.05 using one-way ANOVA with Fisher’s least significant difference post hoc analysis for the indicated comparison. NS, not significant. See also Figure S4.

To further confirm that tissue-inappropriate expression of *IRX3* contributes functionally to the differentiation block in AML, we performed knockdown (KD) experiments in human THP1 AML cells, which exhibit the highest levels of *IRX3* expression among AML cell lines we tested (Figure S4A). *IRX3* KD led to loss of clonogenic potential (Figures 4A–4C), which was due to induction of differentiation, as evidenced by upregulation of the myeloid differentiation markers CD11b, CD14, and CD86 (Figures 4D and 4E) and morphologic analysis (Figure 4F). The proportion of apoptotic cells was unaffected (Figure S4B). To confirm that the observed phenotype was an on-target consequence of *IRX3* KD, similar experiments were performed using KD construct #2, which targets the 3′UTR region of *IRX3*, in a THP1 line engineered to express an *IRX3* cDNA that lacks it. Sustained *IRX3* expression in THP1 cells infected with lentiviral vectors expressing KD construct #2 was confirmed (Figures 4G and 4H), as was rescue of the differentiation phenotype (i.e., loss of clonogenic potential and upregulation of the differentiation marker CD86) (Figures 4I, S4C, and S4D). Similar experiments in murine MLL-AF9 AML cells, which also express *Irx3*, gave similar results: KD cells exhibited loss of clonogenic potential and terminal monocyte/macrophage lineage differentiation (Figures S4E–S4G). *IRX3* KD in a range of additional human AML cell lines also led to loss of clonogenic potential in many cases, but not all (Figure S4H). Of note, KD construct #2 had no effect on the clonogenic potential of K562 cells, which do not express *IRX3* (Figure S4A). Furthermore, the formation of colony-forming unit granulocyte monocyte (CFU-GM) and colony-forming unit monocyte/macrophage (CFU-M) from normal human CD34^+^ cells (which express *IRX3* at very low levels) was unaffected by *IRX3* KD construct #2 (Figures S4I and S4J), although, for unclear reasons, there was a reduction in the formation of erythroid burst-forming units.

Thus, in keeping with *in vitro* and *in vivo* murine experiments, misexpressed *IRX3* contributes to the differentiation block in AML cells and sustains clonogenic potential.

### IRX3 Expression in AML Represses a Myelomonocytic Differentiation Program

**Figure 5:**
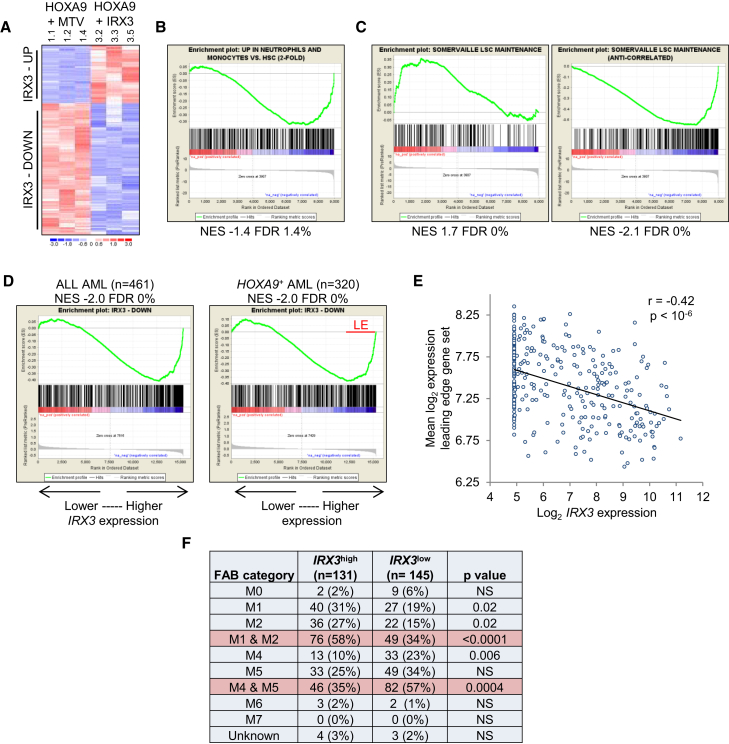
IRX3 Represses a Myelomonocytic Differentiation Program in Murine and Human AML (A) Heatmap shows differentially expressed protein-coding genes (p < 0.05, unpaired t test, >2-fold). (B–D) GSEA plots show enriched expression of (B) mature myelomonocytic genes, (C) LSC maintenance signature genes, and (D) IRX3-repressed genes in (B and C) *Hoxa9*/MTV versus *Hoxa9*/*IRX3* AML cells or (D) primary human AMLs ranked by *IRX3* expression, respectively. (E) Scatterplot shows expression of *IRX3* in primary human *HOXA9*^+^ AML samples versus mean log_2_ expression level for the leading-edge gene set shown in (D). (F) Morphologic classification of *IRX3*^high^ versus *IRX3*^*l*ow^ primary *HOXA9*^+^ AML samples (Wouters et al., 2009). p values were calculated using Fisher’s exact test. See also Figure S5 and Tables S5, S6, and S7.

To evaluate the consequences for the transcriptome of co-expression of *IRX3* with *Hoxa9*, we performed RNA sequencing of flow-sorted KIT^+^Gr1^+^ leukemia cells recovered from the BM of sick mice, three from each cohort. Cells with this immunophenotype are enriched for leukemia-initiating activity in *Hoxa9/Meis1* murine leukemias (Gibbs et al., 2012). Analysis of 8,954 protein-coding genes that passed threshold criteria (i.e., expression >0.5 reads per kilobase per million mapped reads [RPKM] in at least one sample) revealed that 197 were upregulated and 403 downregulated by at least 2-fold in *Hoxa9*/*IRX3* versus *Hoxa9/MTV* leukemias (Figure 5A). Gene Ontology analysis revealed significant enrichment within the *Hoxa9*/*IRX3* downregulated gene set of biological process terms such as “immune response,” “leucocyte activation,” and “defense response” (Table S5), suggesting that IRX3-repressed genes are associated with mature myeloid cells. At similar levels of significance, there were no enriched terms among the *Hoxa9*/*IRX3* upregulated gene set. Gene set enrichment analysis (GSEA) confirmed that co-expression of *IRX3* with *Hoxa9* led to repression of a mature myeloid lineage program in KIT^+^Gr1^+^ leukemia cells, in keeping with morphologic analysis (Figures 3G and 3H). Among genes downregulated in *Hoxa9*/*IRX3* versus *Hoxa9/MTV* AML cells, there was significant enrichment for genes highly expressed in both mature monocytes and mature neutrophils (i.e., myelomonocytic genes) (Figure 5B; Tables S6 and S7).

GSEA also revealed significant overlap with expression patterns observed in murine models of leukemia associated with high-level expression of *Hoxa9* and *Meis1*. Mixed-lineage leukemia (MLL) fusion oncogenes require *Hoxa9* and *Meis1* to properly transform HSPCs and to establish a leukemia cell hierarchy. We observed that in *Hoxa9*/*IRX3* versus *Hoxa9/MTV* AML cells, there was significant enrichment of expression of the transcriptional sub-program that contributes to blocked differentiation and enhanced self-renewal of leukemia stem cells (LSCs) in murine MLL leukemias (LSC maintenance program; Somervaille et al., 2009) (Figure 5C; Tables S6 and S7). Likewise, the set of genes anti-correlated with the LSC maintenance program (i.e., associated with differentiated leukemia cells, downstream of the LSC) was strongly repressed in *Hoxa9*/*IRX3* versus *Hoxa9/MTV* AML cells (Figure 5C; Tables S6 and S7). Similar positive and negative enrichments were observed for gene sets derived from transformed progenitor cells with sustained (HOXA9_MEIS1 POSITIVELY REGULATED) or withdrawn (HOXA9_MEIS1 NEGATIVELY REGULATED) *Hoxa9*/*Meis1* dual expression (Hess et al., 2006) (Figure S5A). All together, these analyses demonstrate that co-expression of *IRX3* with *Hoxa9* enhances expression of genes previously associated with LSC self-renewal and represses expression of a terminal myeloid lineage differentiation program.

We next evaluated whether a signature of IRX3 transcriptional activity could be detected in human AML. We ranked protein-coding genes in *IRX3*^high^ versus IRX3^low^ human AMLs (Wouters et al., 2009) using a signal-to-noise ranking metric (Table S6) and performed GSEA using the set of genes repressed by IRX3 in murine *Hoxa9*/*IRX3* AML cells (Table S5). There was highly significant negative enrichment of IRX3-repressed genes in human *IRX3*^high^ versus *IRX3*^low^ human AMLs whether all AMLs were considered or just those expressing high levels of *HOXA9* (Figure 5D). Indeed, in leading-edge analyses, there was a highly significant association of higher *IRX3* expression in human *HOXA9*^+^ AML with greater repression of IRX3-repressed genes identified in murine leukemias (Figure 5E; Table S7). Remarkably, when the morphologic classification of *HOXA9*^+^ AMLs was considered, among cases with high *IRX3* expression, there were significantly fewer AMLs exhibiting myelomonocytic differentiation (i.e., French-American-British [FAB] M4 subtype) and significantly more AMLs exhibiting minimal differentiation (i.e., FAB M1 subtype) or maturation toward the granulocytic lineage (i.e., FAB M2 subtype) (Figure 5F). Thus, in primary human AML, as in murine AML, misexpression of *IRX3* contributes functionally to blockade of myelomonocytic lineage differentiation.

### IRX3 and FOXC1 Differentially Repress Expression of Myelomonocytic Transcription Factors

We previously reported that the mesenchymal transcription factor gene *FOXC1* is also frequently misexpressed in human AML to confer, in particular, a monocytic lineage differentiation block (Somerville et al., 2015). With regard to the morphologic classification of human AML, *FOXC1*-expressing leukemias were significantly less likely to exhibit monocytic lineage differentiation (i.e., FAB-M5) and significantly more likely to exhibit granulocytic lineage differentiation (i.e., FAB M2) (Somerville et al., 2015). The presence of high *FOXC1* expression in AML was also associated with inferior outcome, in contrast to the presence of high *IRX3* expression (Figure S5B). Murine *Hoxa9/FOXC1* leukemias also exhibited shortened latency versus *Hoxa9/MTV* leukemias (Somerville et al., 2015), in contrast to the latencies observed for *Hoxa9/IRX3* leukemias (Figure 3E).

**Figure 6:**
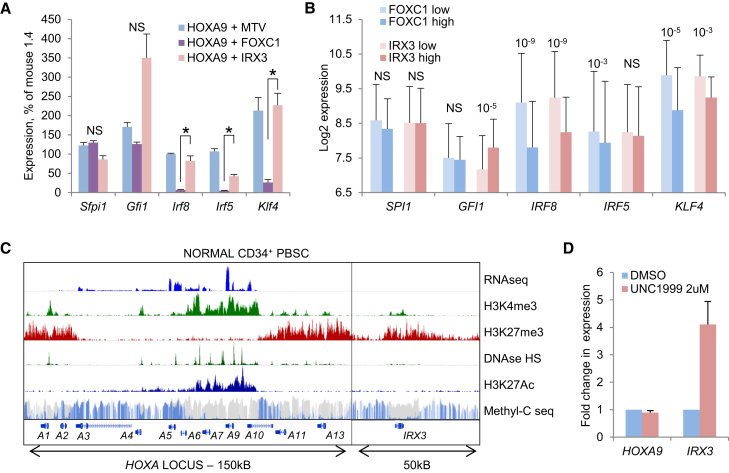
IRX3 Represses Myelomonocytic Genes in Human AML (A) Bar chart shows mean + SEM relative expression of the indicated genes in flow-sorted murine KIT^+^Gr1^+^ BM AML cells (MTV, control, empty vector) (n = 5 or 6 per cohort). ^∗^p ≤ 0.05 using one-way ANOVA with Fisher’s least significant difference post hoc analysis for the indicated comparison. NS, not significant. (B) Bar chart shows mean + SD log_2_ array expression values for the indicated genes in *FOXC1*^high^ (n = 95) versus *FOXC1*^*l*ow^ (n = 175) and *IRX3*^high^ (n = 133) versus *IRX3*^*l*ow^ (n = 158) human *HOXA9*^+^ AML cases (Wouters et al., 2009). p values (unpaired t test) are shown where significant. NS, not significant. (C) Image shows high-throughput sequencing tracks from the ENCODE consortium for the *HOXA* locus and *IRX3*. (D) Bar chart shows mean + SEM relative expression of *IRX3* and *HOXA9* in normal human CD34^+^ cells from separate donors following 5 days of treatment with UNC1999 or DMSO in serum-free liquid culture (n = 4). See also Figure S5.

To evaluate the consequences of misexpressed *IRX3* and *FOXC1* on expression levels of transcription factors required for normal myelomonocytic lineage differentiation, and to determine why the phenotypic consequences of *IRX3* and *FOXC1* misexpression differed one from another, we performed qPCR using murine leukemia samples and analyzed expression levels in published human AML datasets. In flow-sorted murine BM KIT^+^Gr1^+^ AML cells, expression levels of transcription factor genes such as *Irf8*, *Irf5*, and *Klf4* (which promote monocytic lineage differentiation) were significantly lower in *Hoxa9*/*FOXC1* AMLs in comparison with *Hoxa9*/*IRX3* and *Hoxa9*/MTV AMLs (Figure 6A). There was no significant difference in expression levels of the myeloid lineage master regulator *Sfpi1*. In the case of *Gfi1* (which promotes granulocytic lineage differentiation), there was a variable increase in expression in *Hoxa9*/*IRX3* AMLs compared with the other subtypes, although this did not achieve statistical significance.

In primary human AML samples, a very similar pattern was observed. In comparison with *FOXC1*^low^ or *IRX3*^low^ cases, *FOXC1*^high^ or *IRX3*^high^ cases respectively exhibited significantly lower level expression of *IRF8* and *KLF4*, and for both genes, the proportionate reduction in expression was greater for *FOXC1*^high^ cases than for *IRX3*^high^ cases (Figure 6B). *IRF5* expression was significantly lower in *FOXC1*^high^ versus *FOXC1*^low^ cases and no different between the *IRX3* groups, whereas *GFI1* expression was significantly higher in *IRX3*^high^ versus *IRX3*^low^ cases and no different between the *FOXC1* groups. Expression levels of *SPI1* did not differ. Similar analyses using a separate, smaller dataset from TCGA also gave similar results (Figure S5C), in particular with regard to expression levels of *GFI1* and *IRF8*. Together these data demonstrate that although *IRX3* and *FOXC1* misexpression contribute to the differentiation block observed in human AML, they repress myelomonocytic lineage transcription factor genes in a distinctive manner: FOXC1 represses monocytic lineage genes more profoundly than does IRX3, whereas the opposite is the case for GFI1.

### Polycomb Activity Sustains IRX3 Repression in Normal Human CD34^+^ Stem and Progenitor Cells

*IRX3* is only minimally expressed in normal CD34^+^ cells, and its genetic locus is marked by high levels of H3K27 trimethylation (Zhou et al., 2011) (Figure 6C), suggesting that its relative transcriptional silence is maintained by Polycomb-mediated repression. To address this question, normal human CD34^+^ HSPCs from multiple donors were treated *in vitro* for 5 days with a dual EZH1 and EZH2 inhibitor (UNC1999; Konze et al., 2013). We observed a significant increase in *IRX3* expression but no change in *HOXA9* expression (Figure 6D); the *HOXA9* locus is not marked by H3K27 trimethylation in normal CD34^+^ HSPCs (Figure 6C). Thus, the Polycomb complex contributes to continued repression of *IRX3* in normal HSPCs.

### *IRX3* Is Frequently Co-expressed with HOX Genes in Human Acute Lymphoblastic Leukemia

**Figure 7:**
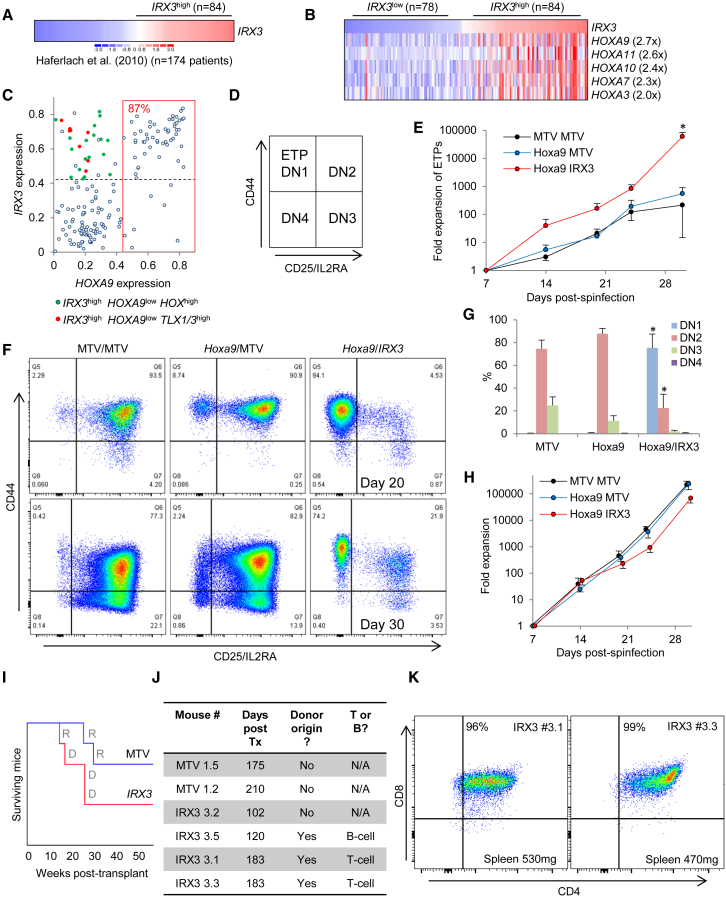
IRX3 Misexpression in Lymphoblastic Leukemia (A) *IRX3* expression in bulk human T-ALL samples. (B) Heatmap shows *IRX3*^high^ versus *IRX3*^low^ T-ALL cases and the most differentially expressed transcription factor genes with mean fold change. (C) *IRX3* versus *HOXA9* expression in primary human T-ALL (Haferlach et al., 2010). Percentage in red text indicates the proportion of *HOXA9*^high^ samples (red box) with high *IRX3* expression (above the dotted gray line). (D) Immunophenotypic definition of early T cell progenitor double-negative (DN) populations. (E) Mean + SEM fold expansion in OP9 DL1 stromal culture of ETP/DN1 cells over input number on day 7 (n = 4–6). MTV, empty vector. (F) Representative flow cytometry plots. (G) Immunophenotypic developmental stage of Lin^neg^ BM HSPCs expressing the indicated gene combinations cultured for 30 days on OP9 DL1 stroma (mean + SEM percentage, n = 6). (H) Mean + SEM fold expansion in OP9 DL1 stromal culture of total cell number over input numbers on day 7 (n = 4–6). For (G) and (H), ^∗^p ≤ 0.05 using one-way ANOVA with Fisher’s least significant difference post hoc analysis for *Hoxa9/IRX3* versus *Hoxa9* and MTV conditions. Murine CD45.1^+^ KIT^+^ BM cells were infected with *IRX3*-expressing or control retroviral vectors, and 96 hr later, 10^6^ drug-resistant cells were transplanted into irradiated CD45.2^+^ congenic recipients. (I) Survival curves. D, donor-derived leukemia; R, recipient-derived leukemia. (J) Survival times and cause of death. (K) Flow cytometry plots show immunophenotype of CD45.1^+^ BM cells at death in the indicated mice. See also Figures S6 and S7.

To provide a more comprehensive evaluation of the role of *IRX3* in human acute leukemia, we analyzed published expression datasets from patients with acute lymphoblastic leukemia (ALL). In a cohort of T-ALL patients (Microarray Innovations in Leukemia [MILE] study; Haferlach et al., 2010), *IRX3* transcripts were detected at high level (i.e., with a probeset [229638_at] value of ≥ 0.42, approximating to a value among the top 25% of array probeset values) in 84 of 174 cases (48%) (Figure 7A). To identify transcription factor genes concordantly expressed with *IRX3* in T-ALL, we compared *IRX3*^high^ (array signal intensity > 0.42, n = 84) with *IRX3*^low^ (array signal intensity < 0.3, n = 78) cases and observed that only *HOXA* genes were upregulated by greater than a mean 2-fold change in array signal intensity (and with p < 10^−5^, by unpaired t test) (Figure 7B). Of the 84 *IRX3*^high^ cases, 52 (62%) expressed *HOXA9* at high level, 18 (21%) were *HOXA9*^low^ but expressed one or more alternate HOX genes, and 8 (14%) were HOX^low^ but instead expressed the homeodomain transcription factors *TLX1* or *TLX3* (Figure 7C). Of the *HOXA9*^high^ T-ALL cases (i.e., 60 of 174), 52 of 60 (87%) were *IRX3*^high^ (Figure 7C). High HOXA expression is a feature of human T-ALL associated with *CALM-AF10* fusions, *SET-NUP214* fusions, *MLL* gene rearrangements, or inv(7) or t(7;7) translocations (resulting in apposition of the HOXA locus to *TCR-β* regulatory elements; Soulier et al., 2005, Van Vlierberghe et al., 2008).

In B-acute lymphoblastic leukemia (B-ALL), high *IRX3* expression was detected in 116 of 563 cases (21%) in the MILE cohort (Figure S6A). There was a particular association of high *IRX3* expression with the presence of an *MLL* gene rearrangement (31 of 70 cases [44%]), or the presence of t(12;21), the cytogenetic hallmark of the *ETV6-RUNX1* fusion (44 of 58 cases [76%]) (Figure S6B). To identify transcription factor genes exhibiting concordant expression with *IRX3* in B-ALL, we compared *IRX3*^high^ (array signal intensity > 0.42, n = 116) with *IRX3*^low^ (array signal intensity < 0.3, n = 398) B-ALLs. Only *HOXA9*, *MEIS1*, and *SOX11* were upregulated by greater than a mean 2-fold change in array signal intensity (and with p < 10^−5^, by unpaired t test) in *IRX3*^high^ B-ALLs (Figure S6B). Of the 57 *MLL* rearranged *HOXA9*^high^ cases, 27 (47%) were *IRX3*^high^ (Figure S6C). *IRX3*^high^ cases with a t(12;21) did not express HOX genes at high level (Figure S6B). Together these data demonstrate that *IRX3*, which is expressed at very low levels in normal hematopoiesis, is frequently highly expressed in human ALL.

### *IRX3* Impedes Phenotypic Differentiation of T Cell Precursors and Induces Lymphoid Leukemias

To evaluate the functional consequences of *IRX3* expression in early stage lymphoid development, Lin^neg^ murine BM HSPCs were double-transduced with pairwise combinations of *IRX3*, *Hoxa9*, or control retroviral vectors and, following drug selection, co-cultured on stromal layers. Where BM cells were co-cultured on OP9 stroma (which supports B-lineage differentiation), we observed no significant difference in upregulation of the B-lineage markers B220 and CD19 or expansion of cell numbers (Figure S6D). In contrast, where cells were co-cultured on OP9 DL1 stroma (which ectopically expresses the Notch ligand DLL1 and supports T-lineage differentiation), we observed a highly significant block in differentiation of *Hoxa9/IRX3* co-expressing cells at the early thymic progenitor (ETP) stage. Differentiating T cell progenitors exhibit sequential expression of CD44 and CD25, which together define developmental stages ETP/DN1 through to DN4 (Figure 7D) (Yui and Rothenberg, 2014). Over the 4 week assay, there was a mean 100-fold greater expansion of cells with an ETP/DN1 immunophenotype in the presence of *Hoxa9/IRX3* co-expression, whereas cells expressing *Hoxa9* alone or control cells readily progressed to DN2 and DN3 downstream differentiation stages (Figures 7E–7G). Overall expansion of cell numbers was similar (Figure 7H). Of note, in contrast to BM HSPCs cultured in myeloid conditions (Figure 2), *IRX3*/MTV expressing Lin^neg^ cells failed to expand on OP9 DL1 stroma (n = 4).

In keeping with a role for IRX3 in promoting the development of lymphoid leukemias, we found that irradiated congenic mice transplanted with *IRX3*-expressing KIT^+^ BM HSPCs developed lymphoid leukemias with incomplete penetrance. In the 12 weeks after transplantation, although there was significantly reduced donor:recipient chimerism in comparison with animals receiving control cells (Figure S6E) (as observed in mice transplanted with *IRX3*/*Hoxa9*-expressing cells; Figure 3D), there was no significant proportional difference in myeloid, B-lineage, and T-lineage engraftment (Figures S6E and S6F). Three of six *IRX3*-expressing HSPC recipients developed donor-derived lymphoid leukemia (Figures 7I and 7J); in two cases, the leukemia was T-lineage, and mice exhibited splenomegaly and near total BM involvement (Figures 7K and S7A). These cases expressed *Hoxa* genes at comparable levels to KIT^+^ BM HSPCs (Figure S7B). In the third case, although the mouse was found dead and detailed autopsy could not be completed, flow cytometry analysis of blood cells performed 8 days before death revealed a CD45.1^lo^B220^+^CD19^+^ population accounting for 92% of donor-derived leucocytes (Figure S7C). This population was not present 4 weeks earlier, suggesting that this mouse died of a B-lineage leukemia. Three other mice died during the 400 day follow-up period, but all succumbed to recipient-derived hematologic malignancies likely induced by irradiation at transplant conditioning (Figures 7I, 7J, and S7A). At experiment termination, there was no evidence of incipient hematologic neoplasms in remaining mice. Together these data demonstrate that *IRX3* expression impedes normal T-progenitor differentiation *in vitro* and induces T-lineage leukemias *in vivo*.

## Discussion

Our studies demonstrate that tissue-inappropriate misexpression of *IRX3* is both frequent and functional in human acute leukemias of multiple lineages. The lack of a major role for *IRX3* and its paralog *IRX5* in normal hematopoiesis is emphasized by the observation of minimal or absent expression in human BM cell populations and genetic knockout experiments that demonstrate that *Irx3*^−/−^ and *Irx5*^−/−^ mice are viable and fertile (Smemo et al., 2014). At least in AML, *IRX3* misexpression is found in both bulk and the putative stem cell compartments, suggesting functional contribution throughout the leukemia clone. With regard to co-regulated gene expression within the *IRX3* topologically associated domain, our qPCR and published dataset analyses indicate that the set of *IRX5*-expressing AMLs is a subset of the *IRX3*-expressing cases, with lower expression levels of *IRX5* than *IRX3*.

Why is *IRX3* expressed so extensively in acute leukemia? Our *in vitro* and *in vivo* studies demonstrate that misexpression of *IRX3* contributes to the cardinal pathologic feature of acute leukemia, the differentiation block. In cultures supporting myeloid lineage differentiation, *IRX3* expression alone or in combination with *Hoxa9* enhanced clonogenic potential and impeded differentiation of normal HSPCs. *In vivo*, *IRX3* co-expression with *Hoxa9* dramatically enhanced the degree of differentiation block in murine AMLs, even though the onset of AML was delayed. Critically, the same transcriptional signature of IRX3-mediated repression of myelomonocytic differentiation was readily identified in human AML, confirming that *IRX3* misexpression is both frequent and functional in human acute leukemia. The paradigm that misexpression of *IRX3* confers a differentiation block is further supported by our observation that co-expression of *IRX3* with *Hoxa9* in T-lineage cultures impeded differentiation of ETPs into downstream developmental stages and that *IRX3*-expressing HSPCs generated lymphoid leukemias *in vivo*. The expanded ETP population may serve as a reservoir for acquisition of genetic mutations required for full-fledged leukemia. The observations that HSPCs expressing *IRX3* alone were immortalized in myeloid culture, failed to expand significantly in OP9 stromal culture, but generated *Hoxa*-expressing lymphoid leukemias *in vivo* reflect the importance of the cellular microenvironment in supporting phenotypic outcome. The outcomes also emphasize the importance of the interaction of misexpressed IRX3 with cell type-specific patterns of chromatin accessibility for transcription factor binding. Cells of different lineages, and of different differentiation states, express different repertoires of transcription factor genes; it is likely that IRX3 binds to chromatin and interferes with gene expression in distinct ways in cells of different lineages.

How is *IRX3* gene expression turned on in hematopoietic cells? Although its expression is positively associated with NPM1 and FLT3 mutations in AML, the link is not absolute; for example, more than a third of NPM1 mutant cases do not express *IRX3* (Wouters et al., 2009). The positive association with other genetic lesions that near invariably lead to high-level HOX gene expression (i.e., a t[6;9] or translocations targeting *MLL* at chromosome 11q23) raises the possibility that *IRX3* is positively regulated by HOX transcription factors. In keeping with this, when *HOXA9* or *HOXA10* is expressed in human CD34^+^ HSPCs, *IRX3* is upregulated (Ferrell et al., 2005). Relatedly, in *Xenopus* development, HOXB4 and IRX5 have overlapping patterns of expression, and the latter is a direct target of the former (Theokli et al., 2003). However, high-level HOX gene expression alone in AML is not sufficient to result in *IRX3* expression, because only 40% of *HOXA9*^+^ cases express *IRX3*. This suggests that additional factors act combinatorially to induce *IRX3*. For example, the Wnt signaling pathway, which is active in AML, induces *IRX3* in forebrain development (Braun et al., 2003). In addition to these candidate positive regulators, it seems likely that loss of repressor activity makes a significant contribution. Our observation that the *IRX3* locus is marked by H3K27me3 in normal CD34^+^ HSPCs and that treatment of cells with a dual EZH1 and EZH2 inhibitor led to *IRX3* upregulation demonstrates that its repression in normal hematopoiesis is dependent on Polycomb.

As well as the strong association of *IRX3* with HOX gene expression in acute leukemias of multiple lineages, there was also high-level expression of *IRX3* in ∼90% of cases of APML and in ∼75% of cases of t(12;21) B-ALL, leukemias that do not express HOX genes. It is possible that *IRX3* is induced as a direct consequence of PML-RARA or ETV6-RUNX1 fusions, respectively, although the close association of co-expressed *SOX11* in the latter case suggests potential collaboration.

The molecular consequences of *IRX3* misexpression in the acute leukemias remain unclear. It is known that TALE family transcription factors such as MEIS1 and PBX can form triple complexes with HOXA9 that bind to PBX-HOXA9 consensus sequences to regulate gene expression (Shen et al., 1999). We speculate that misexpressed IRX3 might alter the function or stability of HOX transcription factor heterotrimeric complexes, perhaps to prevent downregulation of self-renewal genes or upregulation of transcription factors required for terminal differentiation. Alternatively, it may redirect HOX transcription factors to new binding sites or function on its own to activate or repress key transcription regulators.

Like *IRX3*, the Forkhead transcription factor gene *FOXC1* is also frequently misexpressed in AML, although the phenotypic consequences in mouse models and primary human AMLs are quite distinct. This is likely related to distinct mechanisms of action and sites of genomic binding; for example, Forkhead and Iroquois transcription factors have different consensus binding motifs and will bind different sites in the genome to regulate overlapping but fundamentally distinct gene sets. In particular, FOXC1 seems more effective than IRX3 at suppressing expression of monocytic lineage transcription factor genes such as *IRF8*, *IRF5*, and *KLF4*. In contrast, in comparison with *IRX3*^low^ AML cases, in *IRX3*^high^ cases there is increased expression of the granulocytic lineage regulator gene *GFI1*. In some cases both *FOXC1* and *IRX3* are misexpressed, and here the resulting cellular phenotype will represent the integrated consequence of the prevailing nuclear transcription factor milieu.

In summary, we demonstrate that the Iroquois homeodomain transcription factor IRX3 is frequently misexpressed in human acute leukemia to contribute to the differentiation block that is the pathognomonic feature of the disease. Future investigations will identify approaches to target these transcription factors for pro-differentiation therapies to improve patient outcomes.

## Experimental Procedures

### Human Tissue and Ethical Approval

Normal CD34^+^ HSPCs surplus to requirements were from patients undergoing autologous transplantation for lymphoma. Their use was authorized by the Salford and Trafford Research Ethics Committee and, for samples collected since 2006, following written informed consent from donors. Normal human BM was collected with informed consent from healthy adult male donors, with the ethical approval of the Yorkshire Independent Research Ethics Committee. Primary human AML samples were from Manchester Cancer Research Centre’s Tissue Biobank (approved by the South Manchester Research Ethics Committee). Their use was authorized by the Tissue Biobank’s scientific sub-committee, with the informed consent of donors.

### Murine Experiments

Experiments using mice (female, aged 6–12 weeks) were approved by Cancer Research UK Manchester Institute’s Animal Ethics Committee and performed under a project license issued by the United Kingdom Home Office, in keeping with the Home Office Animal Scientific Procedures Act of 1986. C57BL/6 (CD45.2^+^) mice were from Envigo. B6.SJL-*Ptprc*^a^*Pepc*^b^/BoyJ (CD45.1^+^) mice were from Jackson Laboratories and bred in house. Details of transplantation procedures are in the Supplemental Information.

### Reagents, Cell Culture, and Flow Cytometry

Details are in the Supplemental Information.

### RNA Preparation, qPCR, RNA Sequencing, Bioinformatics, and Statistics

Details are in the Supplemental Information.

### Statistical Analyses

Statistical analyses were performed using StatsDirect software version 1.9.7 (StatsDirect), Microsoft Excel, or SPSS for Mac version 22 (IBM). Survival curves were generated using Prism software version 6.0 (GraphPad Software).

## Data and Software Availability

The accession number for the RNA sequencing data files reported in this paper is GEO: GSE97450.

## Supplemental Information

Document S1. Supplemental Experimental Procedures, Figures S1–S7, and Tables S1–S4

Table S5. Genes Up- or Downregulated in Murine Hoxa9/IRX3 Leukemias versus Hoxa9/MTV Leukemias and Gene Ontology Annotations, Related to Figure 5 Differentially expressed (p < 0.05, unpaired t test, two-fold) protein coding genes passing threshold criteria (expression > 0.5 RPKM in at least one sample). Murine genes were converted to human homologs to facilitate cross species comparison.

Table S6. Signal-to-Noise Rankings for Gene Set Enrichment Analyses, Related to Figure 5 Murine gene names were converted to human gene symbols to facilitate cross species comparisons.

Table S7. Gene Sets, Related to Figure 5 Murine genes were converted to human gene symbols to facilitate cross species comparisons. For the “Up in neutrophils and monocytes vs HSC” gene set, normal human BM cells were sorted as described (Huang et al., 2014) and profiled by exon array analysis as described (Somerville et al., 2015). Protein coding genes with expression at least 2-fold higher in normal neutrophils and normal monocytes versus normal HSCs are shown.

Document S2. Article plus Supplemental Information
