# Supplementary material for: Derepression of the Iroquois Homeodomain Transcription Factor Gene IRX3 Confers Differentiation Block in Acute Leukemia
Source: Cell Rep. 2018 Jan 29;22(3):638–52. doi: 10.1016/j.celrep.2017.12.063 (PMC5792454; doi:10.1016/j.celrep.2017.12.063)
Supplement: Document S1. Supplemental Experimental Procedures, Figures S1–S7, and Tables S1–S4 [file mmc1.pdf]

Cell Reports, Volume *22*

Supplemental Information

Derepression of the Iroquois Homeodomain Transcription Factor Gene *IRX3* Confers Differentiation Block in Acute Leukemia

Tim D.D. Somerville, Fabrizio Simeoni, John A. Chadwick, Emma L. Williams, Gary J. Spencer, Katalin Boros, Christopher Wirth, Eleni Tholouli, Richard J. Byers, and Tim C.P. Somervaille

Table S1. Karyotype of 29 AML samples analyzed for *IRX3* expression. Related to Figure 1.

Samples in red exhibit high *IRX3* expression (see Figure 1G). PB = peripheral blood blasts; BM = bone marrow blasts; sl = stemline; sdl = sideline; der = derivative; t = translocation; del = deletion; add = additional material of unknown origin; idem = denotes the stemline karyotype in a subclone; inv = inversion; mar = marker chromosome; cp = composite karyotype.

ID	Sample	Disease Status	Gender	Age	Cytogenetics
54	PB	2nd Relapse	Male	75	47,XY,+11[1]/48,sl,+8[7]/49,sdl,+4[2]
77	BM	Diagnosis	Female	67	Normal
79	BM	Diagnosis	Female	65	47,XX,der(3)t(3;9;14;4)(q2?1;q34;q24;q21),der(4)t(3;9;14;4),del(7)(q22q32),add(8)(q24),der(9)del(9)(p2?3)t(3;9;14;4),der(14)t(3;9;14;4),+18,add(19)(q13.3)x2[9]/46,XX[1]
81	PB	1st Relapse	Male	64	Normal
88	BM	Refractory	Female	64	46,XX,del(12)(p1?2p1?3)[18]/46,XX[2]
93	BM	Diagnosis	Male	59	Normal
103	BM	Diagnosis	Male	51	Normal
104	BM	1st Relapse	Female	31	46,XX,t(6;9;11)(p2?1;p22;q23)[6]/45,idem,der(15)t(15;17)(p11.2;q11.2),-17[4]
107	BM	1st Relapse	Female	16	45,XX,del(7)(q11.2q3?2),t(8;21)(q22;q22),der(12)t(12;18)(p11.2;q11.2),-18[10]/46,XX[1]
114	BM	Diagnosis	Male	56	Normal
120	BM	Diagnosis	Female	68	Normal
122	PB	Diagnosis	Male	66	Normal
127	BM	Diagnosis	Female	63	46,XX,inv(16)(p13q22)[8]/46,XX[2]
136	BM	Diagnosis	Female	61	Normal
144	PB	Diagnosis	Male	71	Normal
146	BM	Diagnosis	Male	52	46,Y,?t(X;6)(p22.1;q?25),t(15;17)(q22;q11.2)[9]/46,XY[1]
149	BM	Diagnosis	Female	49	46,XX,t(15;17)(q22;q11.2)[7]/46,sl,-6,add(16)(q12),+mar[3]/46,XX[3]
155	PB	Diagnosis	Male	63	Normal
163	BM	Diagnosis	Male	20	45,X,-Y,t(8;21)(q22;q22)[8]/46,XY[2]
165	BM	Diagnosis	Female	48	46,XX,t(8;22)(p11;q13),del(9)(q13q32)[10]
170	BM	1st Relapse	Male	50	46,XY,add(6)(p22)[8]/46,XY[2]
189	BM	Diagnosis	Female	76	Normal
191	BM	Diagnosis	Female	38	Normal
222	BM	Diagnosis	Female	80	Normal
228	BM	Diagnosis	Male	54	46,XY,t(1;2)(q2?4;p1?3)[9]/46,XY[1]
232	PB	Diagnosis	Female	85	Normal
234	PB	Diagnosis	Male	84	47,XY,+13[3]/93~94,idemx2[cp3]/92~94<4n>,XXYY,+X,der(5)t(1;5)(q21;q31),+13[cp5]/46,XY[4]
251	BM	Diagnosis	Male	16	46,XY,t(6;9)(p22;q34)[9]/46,XY,der(6)t(6;9),der(9)t(6;9)del(9)(q21q34)[2]
259	BM	Diagnosis	Male	75	47,XY,+8[10]

Table S2. H scoring of trephine biopsy material. Related to Figure 1.

H score and karyotype of 58 AML samples and nine normal samples analyzed for IRX3 protein expression by bone marrow trephine biopsy tissue array. del = deletion; der = derivative; mar = marker chromosome; idem = denotes the stemline karyotype in a subclone; cp = composite karyotype; add = additional material of unknown origin; dic = dicentric; t = translocation; inv = inversion.

Sample number	H score	Karyotype of AML cells or indication for bone marrow (non-malignant)
**1**	0	Weight loss of unclear cause
**2**	0	Normal
**3**	0	Wegener's granulomatosis with pyrexia of unknown origin
**4**	0	Pyrexia of unknown origin
**5**	10	Anaemia
**6**	20	Systemic lupus erythematosus
**7**	20	Normal
**8**	30	Pyrexia of unknown origin
**9**	30	Pyrexia of unknown origin
**1**	0	failed
**2**	0	44X,-Y,t(4;15)(q23;q22),-5,del(17)(p12),+mar1/45X,-Y,idem,+mar2
**3**	0	45XX,der(3)t(3;?11)(p21;q13),-11,-17,+mar1,+?mar2[cp14]/46xx[2]
**4**	0	46,XY,del(7)(q22) [7] / 46,XY [14]
**5**	0	46,XX [73] / 92,XXXX [19]
**6**	0	47,XY,+8[3]/46,XY[9]
**7**	0	Normal
**8**	0	46XX,t(6;9)(q23;q34)/46XX
**9**	0	46XY,add(7)(q3?2),t(8;21)(q22;q22)[2]/45,idem,-Y[8]
**10**	0	Normal
**11**	0	46XX,?del(12)(q24)/46XX
**12**	0	Normal
**13**	0	Normal
**14**	0	42-45,XY,add(1)(q21),-3,-5,add(7)(q3?6),del(11)(q13),-13,-16,dic(16;17)(p11;p11),-21,+2-3 mar[cp3]/83-87,idem[cp3]/46,XY
**15**	0	Normal
**16**	0	failed
**17**	0	46XY,inv(9)(p11;q33)inv(12)(p11;q14)/46XY,inv(9)(p11;q13)
**18**	0	92,XXYY,der(2),t(1;2)(q12;q23)/46XY
**19**	0	45XY,-7/46,idem,+9/46XY
**20**	5	Normal
**21**	10	Normal
**22**	10	Normal
**23**	10	Normal
**24**	20	Normal
**25**	20	Normal
**26**	20	Normal
**27**	20	Normal
**28**	20	46XX,?22p+
**29**	30	Normal
**30**	37.5	failed
**31**	40	Normal
**32**	40	Normal
**33**	45	46-48XX,der(3)t(1;3)(p21;q28),del(5)(q13;q33),der(7)?t(7;13)(q22;q13),-13,add(13)(p12),-21,+mar[cp10]
**34**	45	44,XX,der(4)t(4;12)(p16;q13),-5,-7,-9,-12,+mar1,+mar2
**35**	50	failed
**36**	50	47-50,XY,+1,-5,inv(9)(p11q13),+11,add(17)(p1?1),-18,-22,+r1,+r2,+mar[cp8]
**37**	50	Normal
**38**	70	46XX,del(9)(q21q32),inv(16)(p13q22) [8] /idem,+21 [2]
**39**	80	Normal
**40**	80	Normal
**41**	90	failed
**42**	95	Normal
**43**	105	46XX,t(6;9)(p23;q24)[8]/46XX[5]
**44**	105	Normal
**45**	110	Normal
**46**	120	47XY,+8
**47**	120	45XX,-7,tri(21)(q10)[9]/46XX[2]
**48**	140	Normal
**49**	140	Normal
**50**	140	Normal
**51**	170	failed
**52**	200	Normal
**53**	210	Normal
**54**	230	Normal
**55**	250	failed
**56**	260	Normal
**57**	320	Normal
**58**	350	Normal

Table S3. Association of *IRX3* expression with diagnostic and genetic features in AML. Related to Figure 1.

AML cases were divided into those expressing high or low level *IRX3* based on $\log_2$ expression values of <6.1 or >7.1 respectively (probeset 229638_at). Statistical significance for the indicated comparisons was assessed with Fisher's Exact Test, except for age where an unpaired t-test was used. FAB= French-American-British. NS = not significant.

**Characteristic**		**IRX3^{low} (n=271)**	***IRX3*^{high} (n=159)**	**p value**
**Gender**		137M:134F	79M:80F	NS
**Age**		44 (range 15-61)	43 (range 16-60)	NS
**Cytogenetic risk**	Good	70	24	NS
	Intermediate	137 (51%)	104 (65%)	0.004
	Poor	59	26	NS
	Unknown	5	5	NS
**Karyotype**	Trisomy 8	10	8	NS
	11q23	1 (0%)	7 (4%)	0.005
	5 or 7(q) loss	25 (9%)	4 (3%)	0.009
	Complex	12	3	NS
	Normal	98 (36%)	78 (49%)	0.01
	Other	34	16	NS
	t(6;9)	1 (0%)	5 (3%)	0.03
	t(8;21)	33 (12%)	1 (1%)	<0.0001
	inv(16)	31 (11%)	2 (1%)	<0.0001
	t(15;17)	3 (1%)	21 (11%)	<0.0001
	abn(3q)	2	0	NS
	minus 9q	4	1	NS
	t(9;22)	1	0	NS
	Unknown	16	17	NS
**Gene mutations**	*CEBPA* double	24 (9%)	0 (0%)	<0.0001
	IDH1	12 (4%)	19 (12%)	0.006
	IDH2	21	15	NS
	FLT3 ITD	52 (19%)	66 (42%)	<0.0001
	FLT3 TKD	25	21	NS
	NRAS	34 (13%)	7 (4%)	0.006
	KRAS	2	2	NS
	NPM1	44 (16%)	80 (50%)	<0.0001
	FLT3 ITD and *NPM1*	21 (8%)	44 (28%)	0.001
**High *MECOM***		24 (9%)	2 (1%)	0.001

Table S4. Association of *IRX3* expression with genetic features in AML. Related to Figure 1.

Expression data from 163 AML cases with exome or whole genome sequencing were analyzed using cBioPortal (Cerami et al., 2012; The Cancer Genome Atlas Research Network, 2013). Cases were divided into *IRX3*^{low} and *IRX3*^{high} groups based on log_2 expression values of <6.1 or >7.1 respectively. Statistical significance for the indicated associations was assessed using Fisher's Exact Test.

**Mutated gene**	***IRX3*^{low} (n=101)**	***IRX3*^{high} (n=49)**	**p value**
DNMT3A	25	14	NS
TET2	9	6	NS
NPM1	16 (16%)	24 (50%)	<0.0001
IDH1	8	6	NS
IDH2	13	2	NS
RUNX1	15 (15%)	1 (2%)	0.02
TP53	12 (12%)	0 (0%)	0.009
WT1	4	5	NS
PTPN11	2	4	NS
KIT	5	0	NS
EZH2	3	0	NS
HNRNPK	1	0	NS
U2AF1	4	1	NS
SMC1A	5	0	NS
SMC3	5	1	NS
BRINP3	3	2	NS
PHF6	2	3	NS
STAG2	2	3	NS
RAD21	2	3	NS
FLT3 ITD	11 (11%)	17 (34%)	0.0008
FLT3 D835X	6	4	NS
FLT3 ITD & *NPM1* mutation	3 (3%)	12 (24%)	0.0001

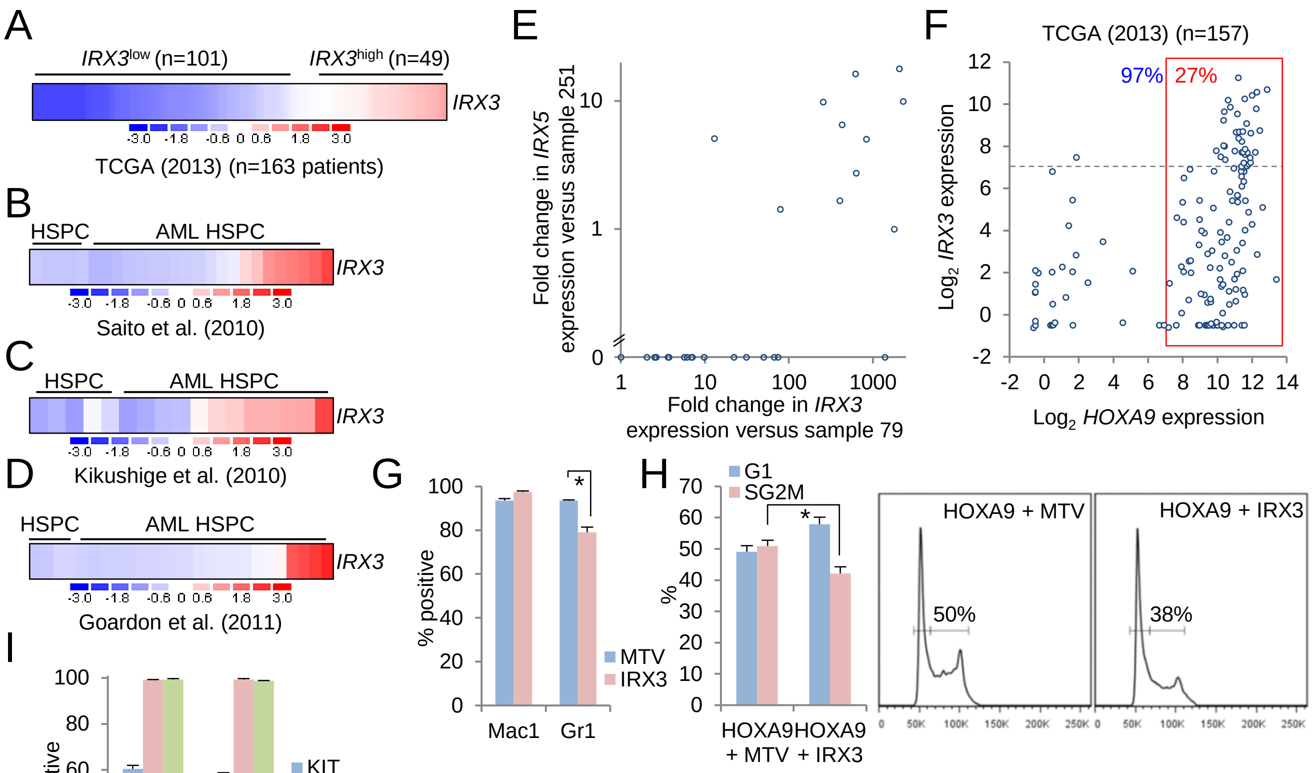

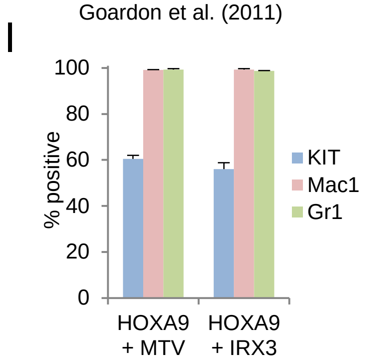

Figure S1. *IRX3* expression in human leukemia. Related to Figures 1-3.

(A) Heat map shows expression of *IRX3* in 163 bulk AML samples (data extracted from cBioPortal; Cerami et al., 2012; The Cancer Genome Atlas Research Network, 2013). Samples were divided into *IRX3*^{low} and *IRX3*^{high} groups based on log_2 expression values of <6.1 or >7.1 respectively. (B-D) Heat maps show the expression of *IRX3* in AML HSPC versus normal adult immunophenotypic BM HSPC. HSPC definitions were (B) $CD34^+CD38^-$ for normal (n=5) and AML cells (n=21); (C) $CD34^+CD38^-Lin^-$ for normal cells (n=5) and $CD34^+CD38^-$ for AML cells (n=12) and (D) $CD34^+CD38^-CD90^+CD45RA^-Lin^-$ for normal cells (n=5) and $CD34^+CD38^+CD123^{+/lo}$ $CD110^-CD45RA^+Lin^-$ (n=21) for AML samples. (E) Scatter plot shows relative expression of *IRX3* versus *IRX5* in primary human bulk AML samples, as determined by quantitative PCR. (F) Scatter plot shows the array expression value of *IRX3* versus *HOXA9* in 157 non-APML primary AML patient samples from the indicated study. Percentage in blue text indicates proportion of *IRX3*^{high} samples exhibiting high *HOXA9* expression; percentage in red text indicates proportion of *HOXA9*^{high} samples exhibiting high *IRX3* expression.

(G) Bar chart shows mean+SEM percentage of cells positive for the indicated cell surface markers as determined by FACS analysis following seven days of liquid culture in conditions favouring myeloid differentiation (n=3).

(H) Bar chart (left panel) shows mean+SEM percentage of cells co-transduced with the indicated retroviral or control (MTV) expression vectors in the indicated stages of the cell cycle (n=3). Representative profiles (right panel) are shown. (I) Bar chart shows mean+SEM percentage of cells positive for the indicated cell surface markers (as determined by flow cytometry) following six days in liquid culture (n=3).

A

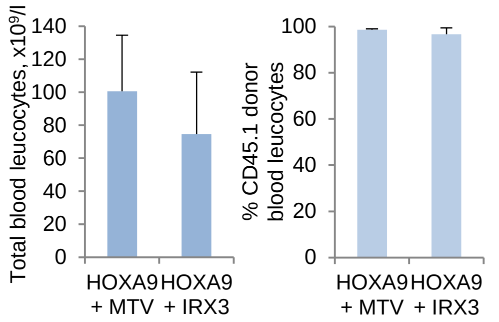

B

Combination	Liver (mg)	Spleen (mg)	Number
Hoxa9/MTV	2313±604	461±66	6
Hoxa9/IRX3	2193±560	447±147	6

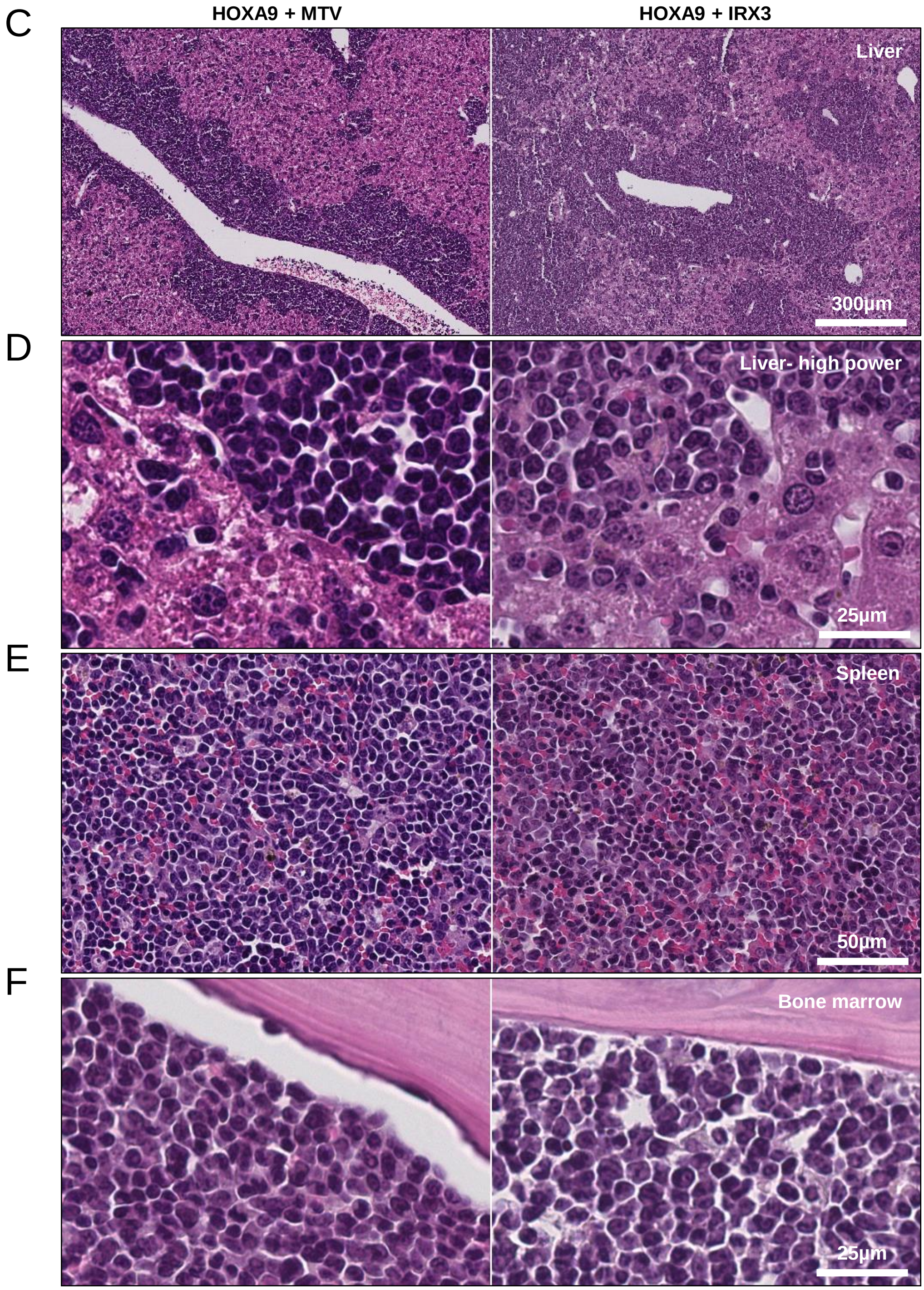

Figure S2. *IRX3* expression enhances the differentiation block in *Hoxa9* induced AML - I. Related to Figure 3.

(A) Bar chart shows mean+SEM total blood leucocyte count at death in the indicated cohorts, as determined by hemocytometer counting (n=4-6 per cohort) (left panel) and the percentage of leucocytes of CD45.1 donor origin. (B) Table shows mean±SD organ masses of leukemic animals at death. Images are representative of (C & D) liver, (E) spleen and (F) BM histology from sick mice.

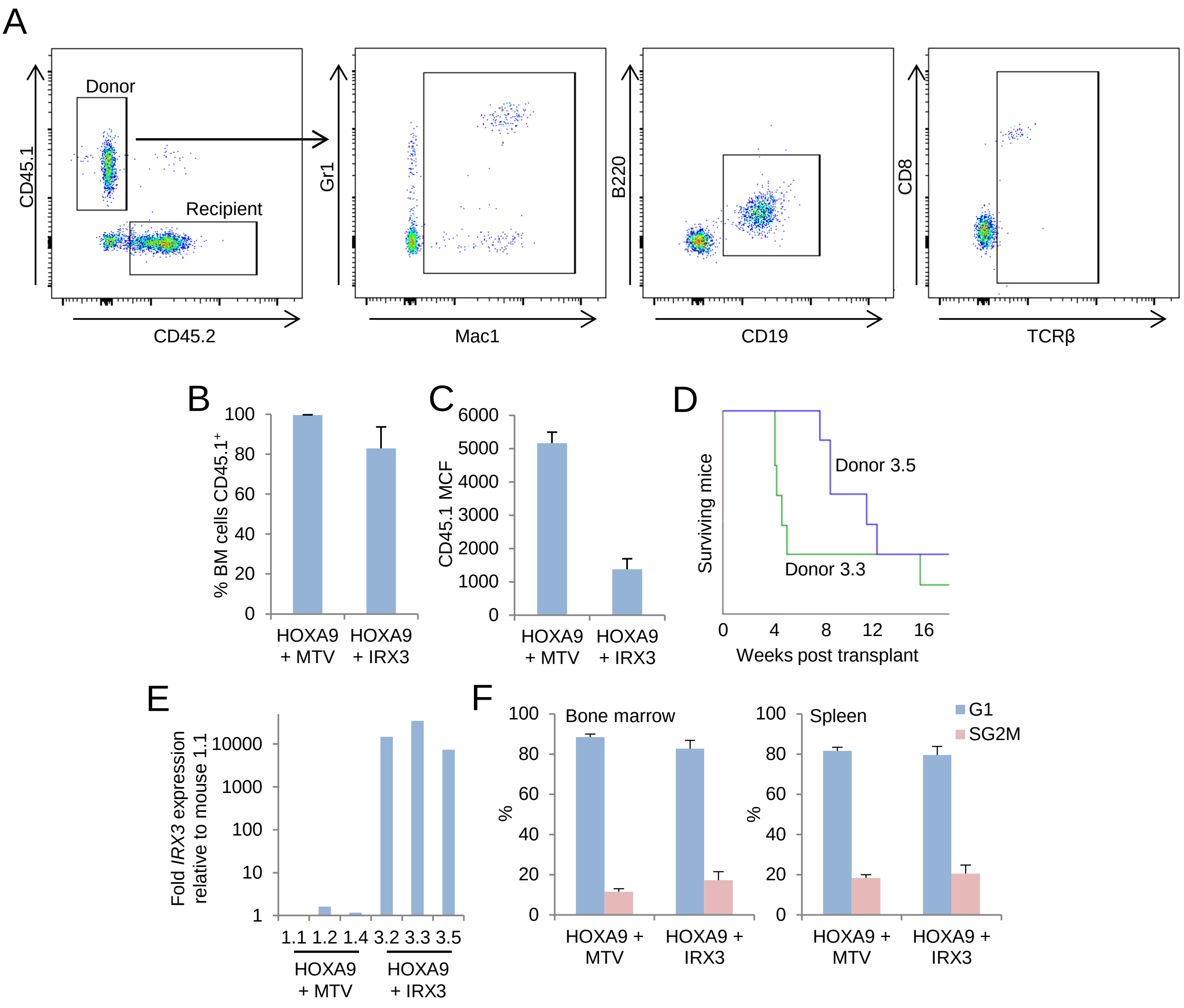

Figure S3. *IRX3* expression enhances the differentiation block in *Hoxa9* induced AML - II. Related to Figure 3.

(A) Representative flow cytometry plots indicate the gating strategy for identifying the percentage of donor-derived CD45.1$^+$ cells, and their lineage contributions, in blood. Bar charts show mean+SEM percentage (B) and mean cellular fluorescence (C) of donor-derived CD45.1$^+$ cells in BM at death (n=6 per cohort). (D) Survival curves of sub-lethally (4.5Gy) irradiated mice secondarily transplanted with 3.3x10^5 (donor 3.3) or 10^6 (donor 3.5) *Hoxa9*/*IRX3* AML cells from the indicated primary recipients. (E) Bar chart shows relative expression of *IRX3* in AML BM cells at the point of death from the indicated recipient mice. (F) Bar charts show mean+SEM percentage of leukemia cells present in the BM (left) and spleen (right) in the indicated phase of the cell cycle at death, as determined by propidium iodide staining and flow cytometry analysis.

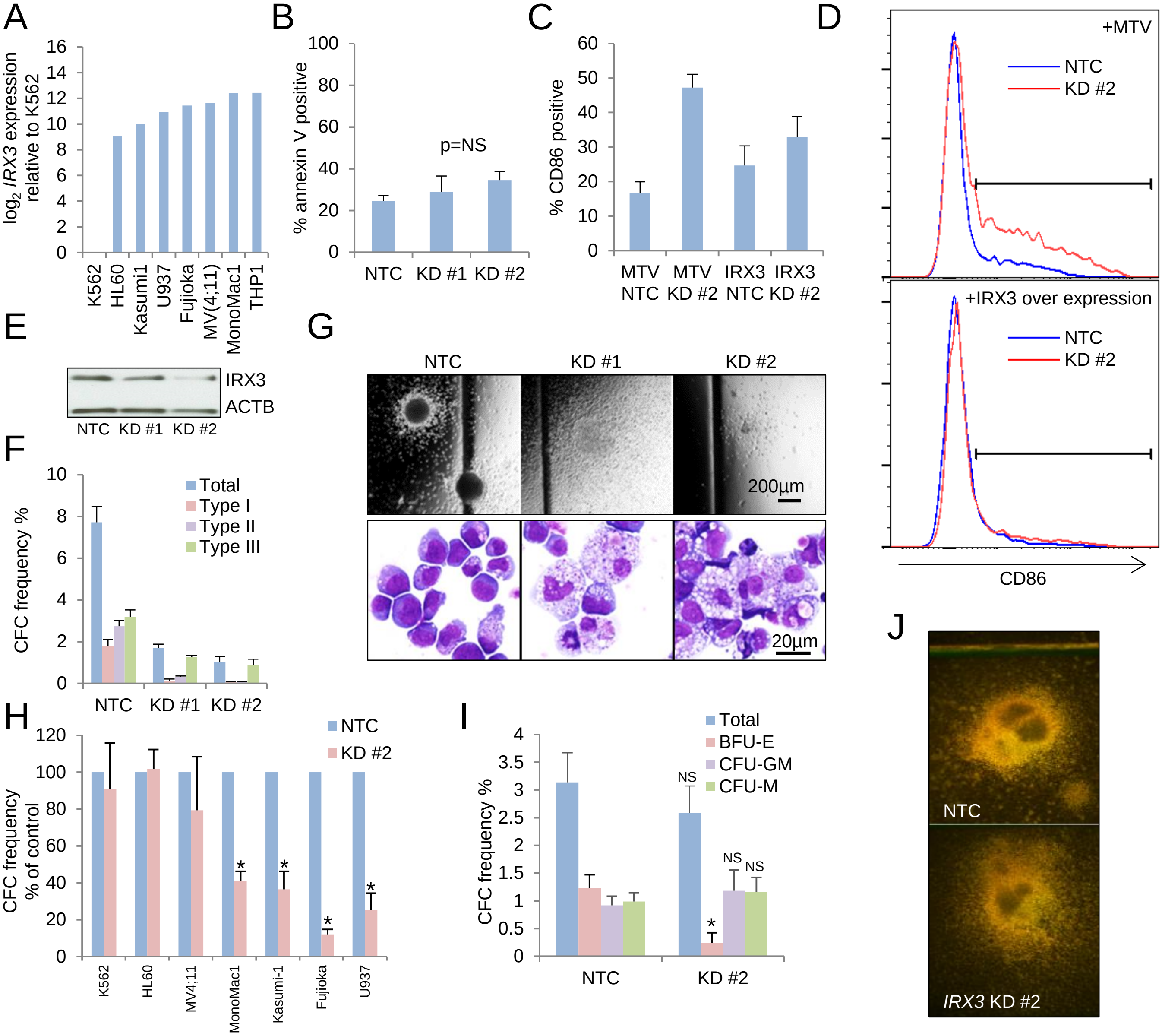

Figure S4. IRX3 sustains the differentiation block and clonogenic potential of AML cells. Related to Figure 4.

(A) Bar chart shows $\log_2$ *IRX3* expression relative to K562 cells in the AML cell lines as determined by qPCR. (B) Bar chart shows mean+SEM apoptotic cells as determined by flow cytometry after seven days of KD (n=3). (C) Bar chart shows mean+SEM percentage of cells positive for the indicated cell surface markers in the indicated conditions, as determined by flow cytometry after six days of KD (n=3). (D) Representative flow cytometry plots from (C). (E-G) Murine MLL-AF9 AML cells were infected with lentiviral vectors targeting *Irx3* for KD (KD#1 and KD#2) or a non-targeting control vector (NTC). (E) Western blot shows expression of the indicated proteins in the indicated conditions. (F) Bar chart shows the mean+SEM colony-forming cell (CFC) frequencies enumerated after seven days (n=3). Type I colonies: contain only blast cells; Type II colonies: mixed blasts and differentiating myeloid cells; Type III colonies: terminally differentiated myeloid cells. (G) Images show representative colonies (top panel) and cytospins (bottom panel) from (F). (H-J) Human AML cell lines (H) or normal human $CD34^+$ HSPCs (I & J) were infected with *IRX3* KD#2 vector or a NTC. (H) Bar chart shows mean+SEM CFC frequencies for KD cells relative to controls, enumerated after ten days (n=3). *indicates $p<0.05$, unpaired t-test. (I) Bar chart shows mean+SEM % total and types of colonies formed by normal human $CD34^+$ HSPCs infected with the *IRX3* KD#2 vector or a NTC (n=3 separate individuals). Colonies were enumerated after 14 days. (J) Representative images of CFU-GM colonies from (H). BFU-E, burst-forming unit erythroid; CFU-GM, colony-forming unit granulocyte/macrophage; CFU-M, colony-forming unit macrophage.

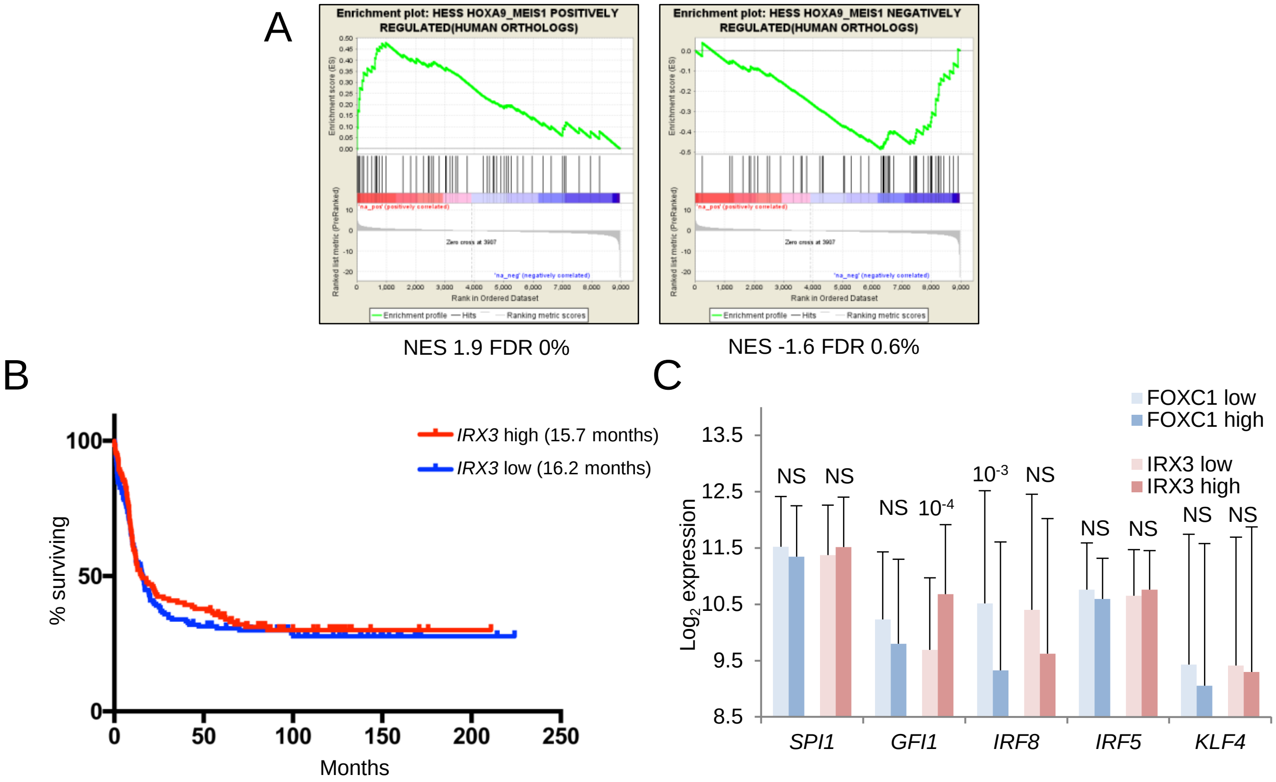

Figure S5. IRX3 represses a myelomonocytic differentiation program in murine and human AML. Related to Figures 5 & 6.

(A) GSEA plots show enriched expression of HOXA9/MEIS1 regulated genes (Hess et al., 2006) in *Hoxa9/IRX3* AMLs versus *Hoxa9/MTV* AMLs.

(B) Survival curves of patients with *IRX3*^{high} versus *IRX3*^{low} AML. (C) Bar chart shows mean+SD $\log_2$ RNAseq expression values for the indicated myelomonocytic transcription factor genes in *HOXA9*$^{+}$ *FOXC1*^{high} (n=45) versus *FOXC1*^{low} (n=69) and *IRX3*^{high} (n=35) versus *IRX3*^{low} (n=81) human AML cases (data extracted from cBioPortal; Cerami et al., 2012; The Cancer Genome Atlas Research Network, 2013). *P* values (unpaired t-test) are shown where significant. NS = not significant.

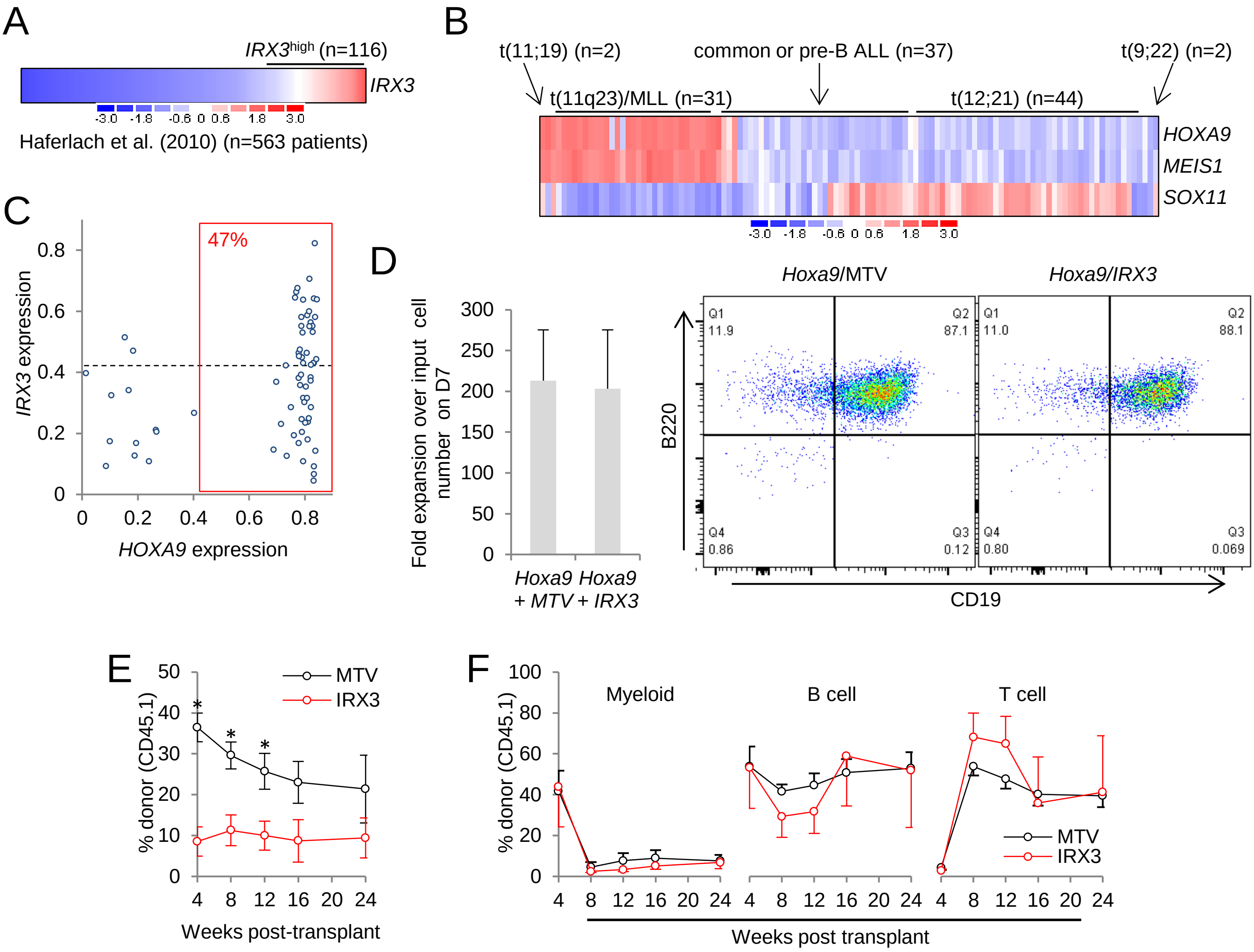

Figure S6. The association of *IRX3* expression with lymphoid leukemia - I. Related to Figure 7.

(A) Heat map shows *IRX3* expression in 563 bulk B-ALL samples (data from Haferlach et al., 2010). The *IRX3*^{high} group was defined by expression values of ≥0.42 (probeset 229638_at). (B) Heat map shows relative expression of the indicated genes in 116 *IRX3*^{high} B-ALL patient samples. (C) Expression values of *IRX3* versus *HOXA9* in primary B-ALL patient samples with an MLL-gene rearrangement (Haferlach et al., 2010). For heat maps, color scale indicates standardized expression level.

(D) Bar chart (left panel) shows mean+SEM fold expansion over input numbers after 21 days of culture on OP9 stroma of Lin^{neg} BM HSPCs expressing the indicated combination of genes following retroviral infection (MTV – empty vector). Right panel shows representative flow cytometry profiles of D21 cells.

(E & F) Murine CD45.1^{+} CD117^{+} BM cells were infected with *IRX3*-expressing or control (MTV) retroviral vectors and 96 hours later 10^{6} drug resistant cells were transplanted into irradiated CD45.2^{+} congenic recipients. Line graphs show the mean±SEM percentage (E) total and (F) lineage specific contribution of donor-derived cells in blood at the indicated times post-transplantation.

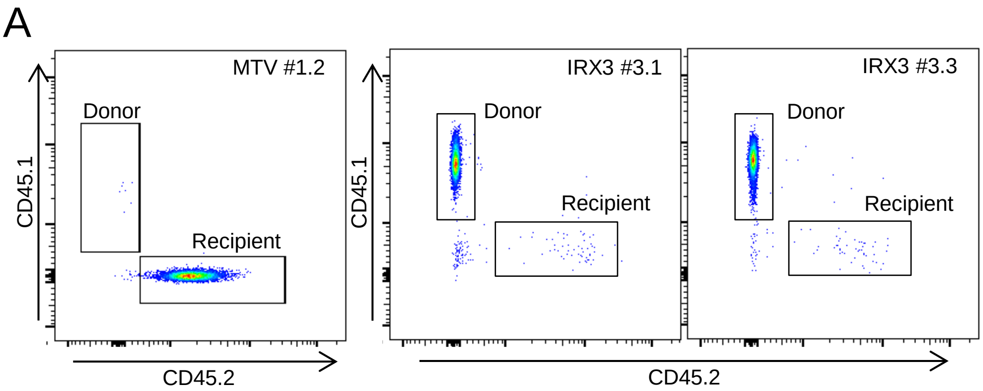

Mouse	Blood leucocyte count ($x10^9$/L)	% donor
3.1	16.5	84
3.3	39.6	96
Normal range	4-7	-

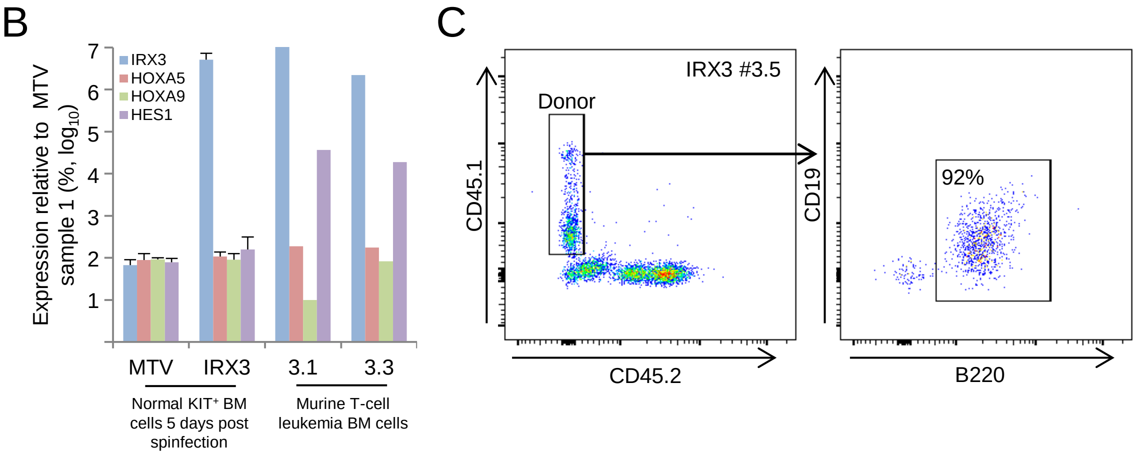

Figure S7. The association of *IRX3* expression with lymphoid leukemia - II. Related to Figure 7.

Murine $CD45.1^+$ $CD117^+$ BM cells were infected with *IRX3*-expressing or control (MTV) retroviral vectors and 96 hours later 10^6 drug resistant cells were transplanted into irradiated $CD45.2^+$ congenic recipients. (A) Representative flow cytometry plots indicate the proportional contribution of donor ($CD45.1^+$) and recipient ($CD45.2^+$) cells to the BM of the indicated mice at death. Table (right panel) shows total white cell counts in blood at death in the indicated mice. (B) Bar chart shows gene expression (as determined by Q-PCR) in the indicated cell populations. For KIT^+ BM cells, mean±SEM for three biological replicates is shown. For murine leukemias, the mean of three technical replicates is shown. (C) Flow cytometry plots show immunophenotype of $CD45.1^+$ nucleated cells in blood in the indicated mouse eight days prior to death.

Supplemental experimental procedures

Murine experiments

For transplantation experiments, BM cells were harvested from the long bones of six to 12 week-old female CD45.1$^+$ mice and KIT$^+$ cells recovered with an AutoMACS Pro device (Miltenyi Biotec, Bergisch Gladbach, Germany). Cells were incubated overnight in RPMI with 20% fetal bovine serum (FBS) (R20) and 20ng/ml SCF, 10ng/ml IL6, 10ng/ml GM-CSF and 10ng/ml IL3 (Peprotech, London, UK). Cells were then spinoculated with retroviral supernatant, prepared as described (Harris et al., 2012), and incubated overnight in R20 with growth factors prior to the addition of puromycin (3µg/ml) and/or neomycin (1.5mg/ml). After 96hr of drug selection $1x10^6$ viable cells (as determined by Trypan blue dye exclusion) were injected into the tail veins of lethally irradiated (800 cGy) recipient six to 12 week-old female CD45.2$^+$ mice. Mice receiving doubly transduced cells received in addition $2x10^5$ CD45.2$^+$ unfractionated BM cells. Tail bleeds were performed monthly and blood donor:recipient chimerism and lineage contributions determined by flow cytometry following ammonium chloride lysis of erythrocytes. Blood leucocytes were counted using a hemocytometer following staining with Türk's solution. Sick mice were euthanized and leukemia cells cryopreserved for later use.

Reagents, plasmids and virus manufacture

Puromycin, neomycin and UNC1999 were from Sigma (St Louis, MO). Polybrene® was from Millipore (Billerica, MA). Retroviral constructs encoding FLAG-Hoxa9 (pMSCVneo and pMSCVpuro) are as described (Somerville et al., 2015). To generate a lentiviral expression vector for *IRX3, IRX3* cDNA (NM_024336.2) was first excised from pOTB7-*IRX3* (Plasmid ID Repository, Harvard Medical School, Boston MA) through triple digest with the *EcoRI*, *XhoI* and *ClaI* restriction enzymes and, using *EcoRI* and *XhoI* restriction sites, sub-cloned into pLentiGS-EGFP (Huang et al., 2014). To generate a retroviral expression vector for *IRX3,* the coding sequence for *IRX3* was PCR amplified from pLentiGS-EGFP-*IRX3* using primers designed to incorporate *EcoRI* and *XhoI* restriction sites, complete with a 3'MYC-tag. Gel purified, amplified DNA fragments were sub-cloned directly into the *EcoRI* and *XhoI* sites of pMSCV(P2)-puro (Somervaille et al., 2009).

Lentiviral vectors for KD experiments were from Sigma: non-targeting control pLKO.1 (SHC002), *IRX3* KD#1 pLKO.1 (TRCN0000016902) and *IRX3* KD#2 pLKO.2 (TRCN0000436197). Lentiviral supernatants were prepared and normal or leukemic human or murine cells infected with viral particles as described (Harris et al., 2012; Somerville et al., 2015).

Cell culture

THP1, HL60 and MonoMac-1 cells were from DMSZ (Braunschweig, Germany). MV(4;11), U937, Fujioka and K562 cells were from Vaskar Saha (Manchester, UK) and Kasumi-1 cells were from Constanze Bonifer (Birmingham, UK). Cell lines were cultured as recommended by DMSZ and their identities verified by STR analysis. Experimentally initiated MLL-AF9 AML cells were generated and cultured as described (Somervaille and Cleary, 2006). Primary normal human CD34$^+$ cells were isolated and cultured as described (Somerville et al., 2015).

For clonogenic assays, human cells were cultured in methylcellulose medium (H4320, Stem Cell Technologies, Vancouver, Canada) and for those involving normal $CD34^+$ cells, the following cytokines were added: 10ng/ml SCF, 10ng/ml IL6, 50ng/ml GM-CSF, 20ng/ml IL3, 10ng/ml IL11, 50ng/ml TPO, 10ng/ml FL, (Peprotech), 50ng/ml G-CSF (Chugai, London, UK) and 4U/ml EPO (Janssen Cilag, Beerse, Belgium). Primary human BM stromal cells from patients with acute promyelocytic leukemia in complete molecular remission were isolated by thawing cryopreserved BM into Minimum Essential Medium-alpha (Sigma) and 15% FBS at a density of 10^6 cells/ml. Three days later non-adherent cells were removed and cells cultured to 80% confluence for three passages prior to RNA extraction. Stromal cells displayed the expected $CD45^{neg}$ immunophenotype demonstrating non-hematopoietic origin.

Clonogenic assays of retrovirally transduced murine KIT^+ BM cells were performed in methylcellulose medium (M3231, Stem Cell Technologies) with 20ng/ml SCF, 10ng/ml IL6, 10ng/ml GM-CSF and 10ng/ml IL3 (Peprotech) and puromycin (3µg/ml) or neomycin (1.5mg/ml) as described (Somerville et al., 2015). For liquid culture of murine myeloid cells, R20 supplemented with the same growth factors and antibiotics (where required) was used.

For stromal B- and T-cell lineage co-cultures, Lin^{neg} BM cells were recovered from C57BL/6 mice using immunomagnetic beads and an AutoMacs Pro device (Miltenyi Biotec) and incubated overnight in R20 with 100 ng/ml SCF, 50 ng/ml TPO and 100 ng/ml FLT3L (Peprotech). Cells were next spinfected with retroviral supernatant and then cultured overnight in the same growth factors. Following 96hr of drug selection with puromycin (3 µg/ml) and neomycin (1.5 mg/ml), cells were washed and seeded onto OP9-GFP (for B lineage differentiation) or OP9-DL1-GFP (for T lineage differentiation) monolayers in 24-well plates in α-MEM medium supplemented with 10% FBS with 5ng/ml FLT3L and 1ng/ml IL7 (Peprotech). OP9 cells were a gift from Georges Lacaud. Co-cultures were harvested by forceful pipetting at the indicated time points (i.e. every 5-7 days) and OP9-GFP/OP9-DL1-GFP clumps cells were eliminated by filtering cells through a 70-µm cell strainer prior to counting, replating and flow cytometric analysis. After Day 12, the IL7 concentration used for OP9-DL1-GFP co-cultures was decreased to 0.25ng/ml.

Flow cytometry, protocols and antibodies

Flow cytometry analyses were performed with a LSR Model II flow cytometer (BD Biosciences, Oxford, UK). Cell sorting experiments were performed with an Influx or FACS Aria II flow cytometer (BD Biosciences). Antibodies for flow cytometry were: (A) human antibodies - KIT-APC (clone 104D2), CD11b-PE (clone ICRF44), CD14-FITC (clone 61D3) and CD86-PerCP-eFluor®710 (clone B7-2); (B) murine antibodies - KIT-APC (clone 2B8), Mac1-PE (clone M1/70), Gr1-PECy7 (clone R86-8C5), F4/80-eFluor®450 (clone BM8), CD45.1-APC (clone A20), CD45.2-PerCPCy5.5 (clone104), CD19-PECy7 (clone eBio1D3), B220-PE (clone RA3-6B2), TCRβ-APCeFluor®780 (clone H57-597), CD3-APC (clone 17A2), CD8a-PE (clone 53-6.7), CD4-eFluor®450 (clone GK1.5), CD44-PECy7 (clone IM7) and CD25-PerCPCy5.5 (clone PC61.5). Antibodies were used at 1/200 dilution except for F4/80-eFluor®450 and TCRβ-APCeFluor®780 (both1/100), and CD8a-PE (1/500). All antibodies were from eBioscience (Hatfield, UK).

Normal human BM populations were isolated as described (Huang et al., 2014; Somerville et al., 2015). Human CD19$^+$ B cells and CD3$^+$CD4$^+$ or CD3$^+$CD8$^+$ T cells were flow sorted from thawed, cryopreserved normal BM samples. Apoptosis was assessed using a BD Pharmingen APC Annexin V kit. Propidium iodide cell cycle analyses were performed as described (Somerville et al., 2015).

Trephine biopsy tissue array immune staining was performed using anti-IRX3 (NBP2-32363, Novus Biologicals, Littleton, CO). Immunoreactivity was semi-quantitatively scored by blind evaluation for intensity (weak, medium or strong) and percentage staining to generate the H-score value.

Antibodies for western blotting were: anti-IRX3 (ab25703, Abcam, Cambridge, UK), anti-MYC-tag (2276, Cell Signaling, Beverley, MA) and anti-ACTB (C4, Millipore). Western blotting was performed as described (Somerville et al., 2015).

RNA preparation and quantitative PCR

RNA was extracted and quantitative PCR performed as described (Somerville et al., 2015). Universal Probe Library System (Roche, Basel, Switzerland) designed primers and probes were as follows:

Gene	Sequence (5'→3')	Probe number
ACTB **F**	ATTGGCAATGAGCGGTTC	11
ACTB **R**	GGATGCCACAGGACTCCAT	
HOXA9 **F**	AAAACAATGCTGAGAATGAGAGC	3
HOXA9 **R**	TATAGGGGCACCGCTTTTT	
IRX3 **F**	AAAAGTTACTCAAGACAGCTT	57
IRX3 **R**	GGATGAGGAGAGAGCCGATA	
SPI1 F	CCACTGGAGGTGTCTGACG	51
SPI1 R	CTGGTACAGGCGGATCTTCT	
IRF5 F	GGCTTCAGGGAGCTTCTCTC	1
IRF5 R	AGGTCTGGCAGGAGCTGTT	
Actb **F**	TGACAGGATGCAGAAGGAGA	106
Actb **R**	CGCTCAGGAGGAGCAATG	
Myc **F**	CCTAGTGCTGCATGAGGAGA	77
Myc **R**	TCCACAGACACCACATCAATT	
Myb **F**	CCTTCTCTCCCTCGCAGTT	11
Myb **R**	TGGAGGGTAAGGTAGGTGCAT	
Spi1 **F**	ATGGAGAAGCTGATGGCTTG	27
Spi1 **R**	GGAACTGGTACAGGCGAATC	
Gfi1 **F**	ATGTGCGGCAAGACCTTC	1
Gfi1 **R**	ACAGTCAAAGCTGCGTTCCT	
Irf8 **F**	CCGGCAAGCAGGATTACA	10
Irf8 **R**	GCTTTGTCTCCCTCTTTAAACTTC	
Irf5 **F**	AACCCACTGATGATTACGTTCTG	101
Irf5 **R**	GCCTGGTAGCATTCTCTGGA	
Klf4 **F**	GCTCCTCTACAGCCGAGAATC	10
Klf4 **R**	ATGTCCGCCAGGTTGAAG	

TaqMan primer/probe assays (Life Technologies, Carlsbad, CA) were used for *KLF4* (Hs00358836_m1), *GFI1* (Hs00382207_m1), *IRX5* (Hs04334749_m1) and *IRF8* (Hs00175238_m1). ΔCt values relative to *ACTB* or *Actb* were assessed using SDS software v2.1 (Applied Biosystems, Foster City, CA).

Bioinformatics and RNA sequencing

Analyses of published microarray and RNA sequencing datasets were performed as described (Somerville et al., 2015). Murine gene symbols were converted to human orthologs as described (Harris et al., 2012). For RNA sequencing analysis of murine leukemia samples, BM from sick mice (three animals per cohort) was thawed, and donor derived ($CD45.1^+$) viable KIT^+Gr1^+ leukemia cells were flow sorted. RNA was extracted and its quality confirmed using an Agilent Bioanalyzer (Agilent Technologies, Santa Clara, CA). Indexed PolyA libraries were prepared using 200ng of total RNA and 14 cycles of amplification with the Agilent SureSelect Strand Specific RNA Library Prep Kit for Illumina Sequencing (Agilent, Santa Clara, CA). Libraries were quantified by quantitative PCR using a Kapa Library Quantification Kit for Illumina sequencing platforms (Roche). Paired-end 75bp sequencing was carried out by clustering 1.8 pM of the pooled libraries on a NextSeq 500 sequencer (Illumina, San Diego, CA). Reads were aligned to the mouse genome (GRCm38) with Bowtie2, version 2.2.1 (Langmead et al., 2012) using default settings. SAMtools (v 0.1.19) was used to filter out unaligned reads, non-primary alignments or alignments with a Phred quality score of <20. There were 18.5-21.8 million input reads per sample and 8.3-10.2 million reads per sample remained after filtering. Reads were mapped to annotated protein coding genes (ENSEMBL v76) using the Annmap database, R and Bioconductor (Gentleman et al., 2004). RPKM (reads per kilobase per million uniquely mapped reads) was computed for each gene.

Supplemental references

Gentleman RC, Carey VJ, Bates DM, Bolstad B, Dettling M, Dudoit S, Ellis B, Gautier L, Ge Y, Gentry J et al. 2004. Bioconductor: open software development for computational biology and bioinformatics. *Genome Biology* **5**: R80.

Harris WJ, Huang X, Lynch JT, Spencer GJ, Hitchin JR, Li Y, Ciceri F, Blaser JG, Greystoke BF, Jordan AM et al. 2012. The histone demethylase KDM1A sustains the oncogenic potential of MLL-AF9 leukemia stem cells. *Cancer Cell* **21**: 473-487.

Huang X, Spencer GJ, Lynch JT, Ciceri F, Somerville TD, Somervaille TC. 2014. Enhancers of Polycomb EPC1 and EPC2 sustain the oncogenic potential of MLL leukemia stem cells. *Leukemia* **28**: 1081-1091.

Langmead B, Salzberg SL. 2012. Fast gapped-read alignment with Bowtie 2. *Nature Methods* **9**: 357-359.
