# Supplementary material for: Derepression of the Iroquois Homeodomain Transcription Factor Gene IRX3 Confers Differentiation Block in Acute Leukemia
Source: Cell Rep. 2018 Jan 29;22(3):638–52. doi: 10.1016/j.celrep.2017.12.063 (PMC5792454; doi:10.1016/j.celrep.2017.12.063)
Supplement: Document S2. Article plus Supplemental Information [file mmc5.pdf]

Cell Reports

Article

Derepression of the Iroquois Homeodomain Transcription Factor Gene *IRX3* Confers Differentiation Block in Acute Leukemia

Graphical Abstract

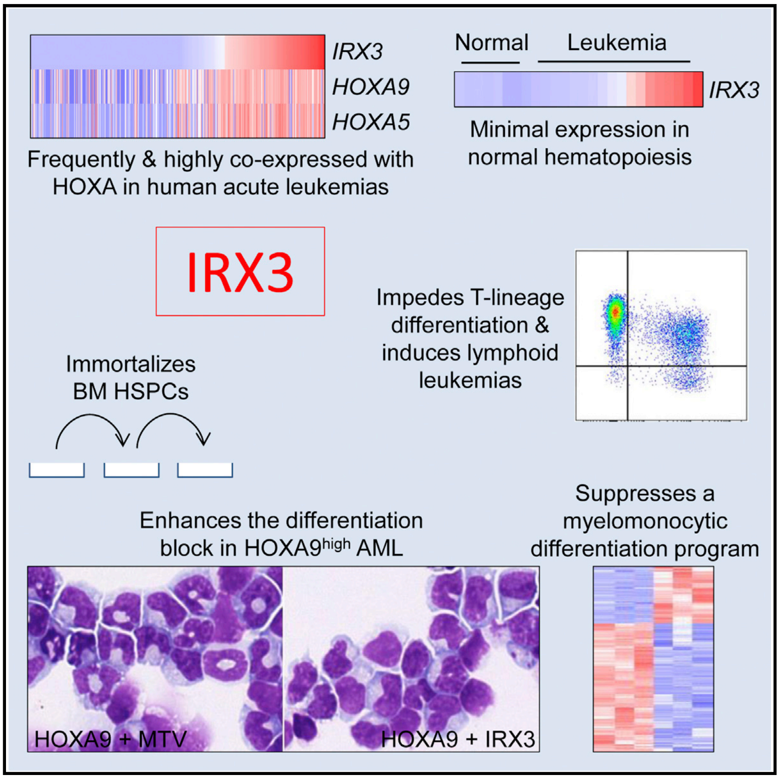

Authors

Tim D.D. Somerville, Fabrizio Simeoni, John A. Chadwick, ..., Eleni Tholouli, Richard J. Byers, Tim C.P. Somervaille

Correspondence

tim.somervaille@cruk.manchester.ac.uk

In Brief

Somerville et al. report that the Iroquois homeodomain transcription factor gene *IRX3* is frequently derepressed in human acute leukemias of multiple lineages and contributes to the differentiation block, which is the pathognomonic feature of the disease.

Highlights

- *IRX3* is derepressed in ~30% of AML, ~50% of T-ALL, and 20% of B-ALL
- IRX3 suppresses a mature myelomonocytic differentiation program in AML
- IRX3 impairs T-lineage differentiation, expanding an early T-progenitor population
- IRX3 contributes to the differentiation block that is characteristic of the disease

Data and Software Availability

GSE97450

Somerville et al., 2018, Cell Reports *22*, 638–652
January 16, 2018 © 2018 The Authors.
https://doi.org/10.1016/j.celrep.2017.12.063

CellPress

OPEN ACCESS
CellPress

Cell Reports
Article

Derepression of the Iroquois Homeodomain Transcription Factor Gene *IRX3* Confers Differentiation Block in Acute Leukemia

Tim D.D. Somerville,[1] Fabrizio Simeoni,[1] John A. Chadwick,[1] Emma L. Williams,[1] Gary J. Spencer,[1] Katalin Boros,[2] Christopher Wirth,[3] Eleni Tholouli,[4] Richard J. Byers,[2] and Tim C.P. Somervaille[1,5,*]
[1]Leukaemia Biology Laboratory, Cancer Research UK Manchester Institute, The University of Manchester, Manchester M20 4BX, UK
[2]Department of Histopathology, Manchester University NHS Foundation Trust, Manchester M13 9WL, UK
[3]Applied Computational Biology and Bioinformatics Group, Cancer Research UK Manchester Institute, The University of Manchester, Manchester M20 4BX, UK
[4]Department of Haematology, Manchester University NHS Foundation Trust, Manchester M13 9WL, UK
[5]Lead Contact
*Correspondence: tim.somervaille@cruk.manchester.ac.uk
https://doi.org/10.1016/j.celrep.2017.12.063

SUMMARY

The Iroquois homeodomain transcription factor gene *IRX3* is expressed in the developing nervous system, limb buds, and heart, and transcript levels specify obesity risk in humans. We now report a functional role for *IRX3* in human acute leukemia. Although transcript levels are very low in normal human bone marrow cells, high *IRX3* expression is found in ∼30% of patients with acute myeloid leukemia (AML), ∼50% with T-acute lymphoblastic leukemia, and ∼20% with B-acute lymphoblastic leukemia, frequently in association with high-level HOXA gene expression. Expression of *IRX3* alone was sufficient to immortalize hematopoietic stem and progenitor cells (HSPCs) in myeloid culture and induce lymphoid leukemias *in vivo*. *IRX3* knockdown induced terminal differentiation of AML cells. Combined *IRX3* and *Hoxa9* expression in murine HSPCs impeded normal T-progenitor differentiation in lymphoid culture and substantially enhanced the morphologic and phenotypic differentiation block of AML in myeloid leukemia transplantation experiments through suppression of a terminal myelomonocytic program. Likewise, in cases of primary human AML, high *IRX3* expression is strongly associated with reduced myelomonocytic differentiation. Thus, tissue-inappropriate derepression of *IRX3* contributes significantly to the block in differentiation, which is the pathognomonic feature of human acute leukemias.

INTRODUCTION

The cardinal pathologic feature of the acute leukemias is a block to normal blood cell differentiation that results in an accumulation in the bone marrow (BM) of incompletely differentiated blast cells and failure of normal hematopoiesis (Wiseman et al., 2014). Although the spectrum of mutations associated with these diseases is now well established, the biologic basis of how mutations interact with one another to establish the pathognomonic differentiation block is less well understood. We recently reported that the Forkhead box transcription factor FOXC1 is misexpressed in approximately 20% of patients with acute myeloid leukemia (AML), in particular in those cases exhibiting high HOXA/B gene expression (Somerville et al., 2015). *FOXC1* is neither required for nor expressed in normal hematopoietic cells but is essential for normal development of mesenchymal tissues such as the skeleton, heart, and eye and for the normal function of BM niche cells (Omatsu et al., 2014). Its misexpression in leukemic hematopoiesis contributes to a block in differentiation along both monocytic and B cell lineages and is associated with inferior survival. Given the lack of one-to-one correlation with any specific mutation, tissue-inappropriate derepression of *FOXC1* is paradigmatic for a non-mutational mechanism contributing to cellular transformation in myeloid malignancy.

Whether tissue-inappropriate misexpression of other transcription factors contributes to the differentiation block of leukemia is not known. One candidate is the Iroquois homeobox transcription factor gene *IRX3*, which, like *FOXC1*, is expressed in a significant proportion of patients with AML (Somerville et al., 2015) but minimally expressed in both normal hematopoietic stem and progenitor cells (HSPCs) and mature blood cells (ENCODE data; Zhou et al., 2011). IRX3 is a member of the three-amino-acid-loop-extension (TALE) superfamily of homeodomain transcription factors, which also includes MEIS1 and PBX1 (Mukherjee and Bürglin, 2007). In embryogenesis, it is strongly expressed in the developing nervous system, as well as in mesoderm-derived tissues such as the limb buds, kidney, and heart (Houweling et al., 2001). Of note, the developmental expression pattern of the *Irx3* paralog *Irx5*, which sits in the same 2MB topologically associating domain, is strikingly similar (Cohen et al., 2000; Claussnitzer et al., 2015). These genes exhibit functional redundancy because although *Irx3*-null and *Irx5*-null mice are viable and fertile, mice lacking both genes die in utero because of severe cardiac and skeletal defects (Zhang et al., 2011; Gaborit et al., 2012; Li et al., 2014; Smemo et al., 2014). Interestingly, non-coding variation in an enhancer

This is an open access article under the CC BY license (http://creativecommons.org/licenses/by/4.0/).

region 500 kb downstream of *IRX3* provides the strongest genetic association with risk for human obesity. Pertinent to this, adult *Irx3*-null mice display a 25%–30% reduction in body weight due to loss of fat mass, increased basal metabolic rate, and browning of white adipose tissue, attributable to loss of hypothalamic (Smemo et al., 2014) or preadipocyte (Claussnitzer et al., 2015) *Irx3* expression. The rs1421085 single-nucleotide variant present in the obesity risk region dictates the extent of local recruitment of ARID5B to the *IRX3* enhancer, with consequent regulation of *IRX3* expression (Claussnitzer et al., 2015).

Whether *IRX3* has a role in human malignancy is unclear. One study reported that *IRX3* is strongly expressed in colorectal adenomas in comparison with normal mucosa and negatively regulates TGF-β signaling in colorectal cancer cell lines (Martorell et al., 2014). However, little else is known. Given this, and the observation that *IRX3* is highly expressed in a subset of AML patients, we evaluated whether *IRX3* has a functional role in acute leukemia.

RESULTS

IRX3 Is Frequently Co-expressed with HOX Genes in Human AML

To ascertain the frequency and extent of *IRX3* expression in AML and in flow-sorted normal human BM cell populations, we performed both qPCR and analyses of published datasets. In a Dutch cohort of AML patients treated intensively with anthracycline-based chemotherapy on the Hemato-Oncologie voor Volwassenen Nederland (HOVON) protocols, *IRX3* transcripts were detected at high level (i.e., with a probeset [229638_at] value of $\log_2 > 7.1$, approximating to a value among the top 25% of array probeset values) in 159 of 461 bulk presentation samples (34%) (Wouters et al., 2009) (Figure 1A). Likewise, in The Cancer Genome Atlas Research Network series, 49 of 163 cases (30%) expressed *IRX3* at high level (Cerami et al., 2012; Ley et al., 2013) (Figure S1A). In flow-sorted populations of AML cells with immature immunophenotypes, *IRX3* was highly expressed (i.e., among the top 25% of array probeset values) in 33% (Saito et al., 2010) (Figure S1B), 58% (Kikushige et al., 2010) (Figure S1C), and 19% (Goardon et al., 2011) (Figure S1D) of samples. Concomitant microarray profiling of normal human immunophenotypic HSPCs suggested low or absent *IRX3* expression (Figures S1B–S1D). In keeping with this, our qPCR analyses revealed very low levels of *IRX3* transcripts in all normal BM populations tested, but in 10 of 29 AML samples (34%), *IRX3* expression was increased at least 250-fold over levels observed in the lowest expressing AML sample (Figure 1B; Table S1). *IRX3* transcript levels were higher in normal human CD45^{neg} BM stromal cells than in normal BM cell populations (Figure 1B) but not as high as those observed in many AML samples. Given that expression of *IRX3* and *IRX5* are co-regulated in development, we also performed qPCR for *IRX5* transcripts. We did not detect *IRX5* expression in normal human BM cell populations (data not shown) but did detect low-level expression in 11 of 28 AML samples (39%), typically in cases with high *IRX3* expression (Figure S1E). In the Dutch AML cohort, 44 of 461 cases (10%) expressed *IRX5* at high level (i.e., probeset [210239_at] value of $\log_2 > 7.1$), and in every case there was also high IRX3 expression (data not shown). Thus, the set of *IRX5*^{high} AML cases is a subset of the group of *IRX3*^{high} cases.

To confirm that IRX3 protein was also expressed in AML, we performed immunohistochemical staining of a trephine biopsy tissue array. The array included 58 samples from patients with AML and 9 samples from patients with non-malignant BM conditions (Table S2). Immune staining was H-scored by blind evaluation. Strong or moderate nuclear immune staining (i.e., H score $\geq$ 80) was observed in 20 of 58 cases (34%). Weak or absent immune staining was observed in 38 of 58 AML cases (66%) and in all non-malignant cases (Figures 1C and 1D).

High *IRX3* expression in AML was strongly and positively associated with the presence of an *NPM1* mutation or a *FLT3* internal tandem duplication (Tables S3 and S4) (Wouters et al., 2009; Ley et al., 2013). A strong positive association with acute promyelocytic leukemia (APML) was also noted (Table S3). There were weaker positive associations with intermediate cytogenetic risk, normal karyotype and the presence of an *IDH1* mutation, t(6;9), or an *MLL* gene rearrangement. High *IRX3* expression was negatively associated with the presence of chromosome 5 or 7(q) loss, the presence of t(8;21) or inv(16), the presence of mutations in *NRAS*, *TP53*, or *RUNX1*, or a double *CEBPA* mutation and the presence of high *MECOM* expression (Tables S3 and S4). Although detailed genotyping of the tissue array samples was not performed, there was nevertheless a significant association of normal karyotype with strong or moderate IRX3 expression (14 of 17 available karyotypes were normal where strong or moderate nuclear immune staining was present versus 17 of 34 with weak or absent staining, $p = 0.035$, Fisher's exact test) (Table S2).

To identify genes co-expressed with *IRX3* in human AML, we next compared *IRX3*^{high} AMLs (probeset 229638_at value of $\log_2 > 7.1$) with *IRX3*^{low} AMLs (probeset 229638_at value of $\log_2 < 6.1$) (Wouters et al., 2009) and found *HOXA9* and *HOXA5* to be the most differentially expressed transcription factor genes in the *IRX3*^{high} group whether (data not shown) or not (Figure 1E) cases of APML (which do not express HOX genes) were included. Of the non-APML cases, 133 of 138 *IRX3*^{high} samples (96%) exhibited high *HOXA9* expression (i.e., probeset value of $\log_2 > 7.1$), and of the *HOXA9*^{high} samples, 133 of 319 (42%) expressed high levels of *IRX3* (Figure 1F). Of the five *IRX3*^{high} *HOXA9*^{low} samples, three expressed one or more alternative HOX genes at significant level (i.e., $\log_2 > 7.1$), indicating that overall, 98.6% of *IRX3*^{high} non-APML samples exhibited HOX gene co-expression. Similar results were observed in analyses of The Cancer Genome Atlas (TCGA) dataset (Figure S1F) (Ley et al., 2013) and qPCR analysis of our own samples (Figure 1G). Taken together, these data demonstrate that in human AML, the Iroquois homeodomain transcription factor gene *IRX3*, which is minimally expressed in normal hematopoiesis, is often misexpressed in conjunction with high HOX gene expression, as well as in APML. The former association suggests an explanation for the statistically significant associations of *IRX3* expression with the presence of an *NPM1* mutation, *MLL* gene rearrangements, and a t(6;9) because these molecular subtypes are associated with high HOX gene expression.

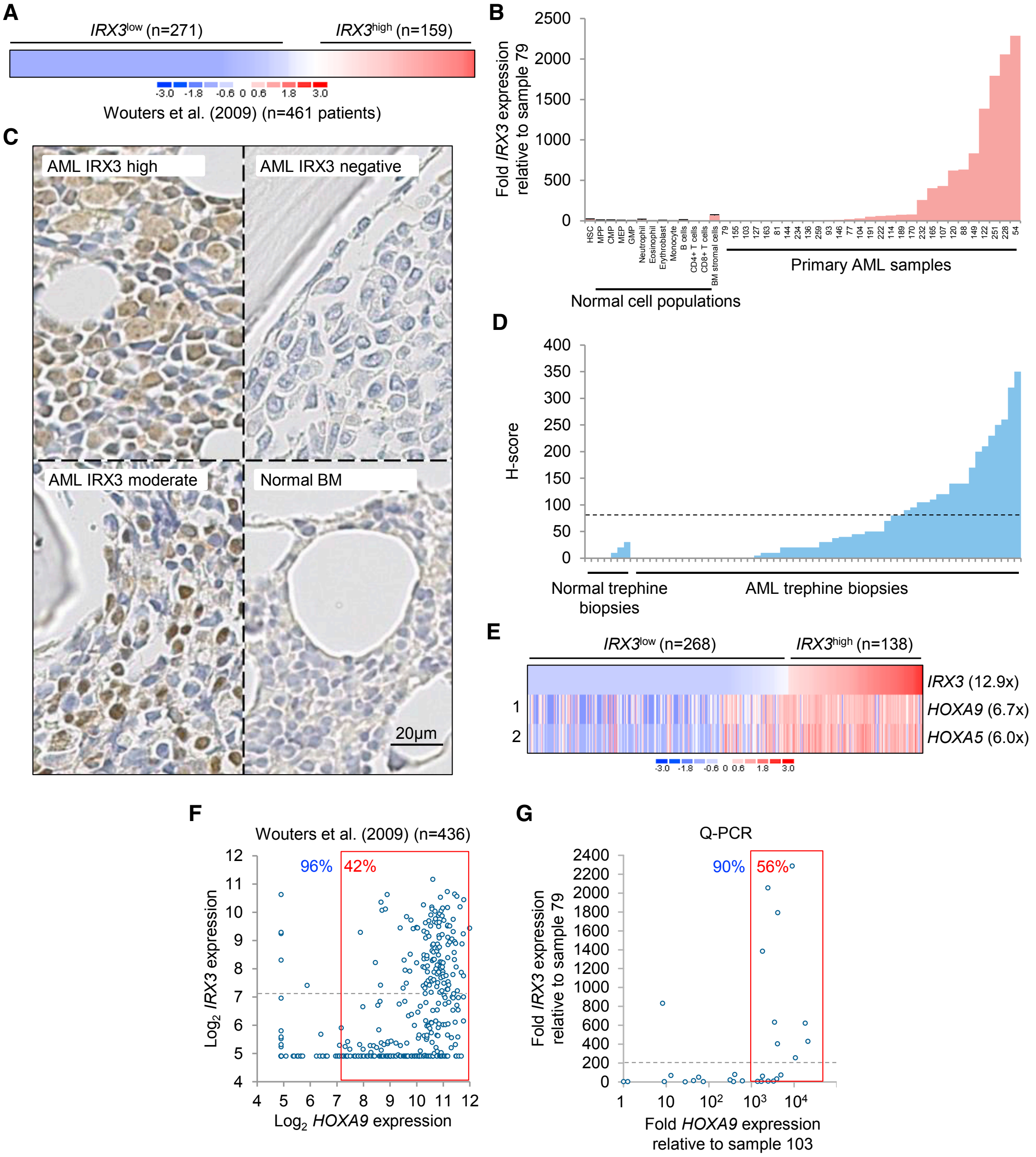

Figure 1. *IRX3* Expression in Human AML

(A) *IRX3* expression in bulk human AML samples.

(B) Relative *IRX3* expression in bulk primary human AML samples (n = 29) and normal human BM cell populations (n = 4 separate individuals per cell type; error bars = SEM). BM, bone marrow; CMP, common myeloid progenitor; Eosin, eosinophils; EryB, erythroblast; GMP, granulocyte-macrophage progenitor; HSC, hematopoietic stem cell ($CD34^{+}38^{-}90^{+}45RA^{-}Lin^{-}$); MEP, megakaryocyte-erythrocyte progenitor; Mono, monocytes; MPP, multipotent progenitor; Neut, neutrophils. AML sample numbers refer to Biobank identifier.

(C) Representative images of IRX3 immune staining of human BM trephine biopsies.

(D) H scores for IRX3 immune staining. Dashed line indicates cutoff value for moderate/strong immune staining.

(E) Heatmap shows $IRX3^{high}$ versus $IRX3^{low}$ AML cases (excluding APML) (Wouters et al., 2009) and the most differentially expressed transcription factor genes with mean fold change.

(F and G) Scatterplots show *IRX3* versus *HOXA9* expression in primary AML samples as determined by (F) array values or (G) qPCR. For scatterplots, percentages in blue text indicate proportion of $IRX3^{high}$ samples exhibiting high *HOXA9* expression. Percentages in red text indicate the proportion of $HOXA9^{high}$ samples (in the red boxes) additionally exhibiting high *IRX3* expression (above the dotted gray line).

See also Figure S1 and Tables S1–S4.

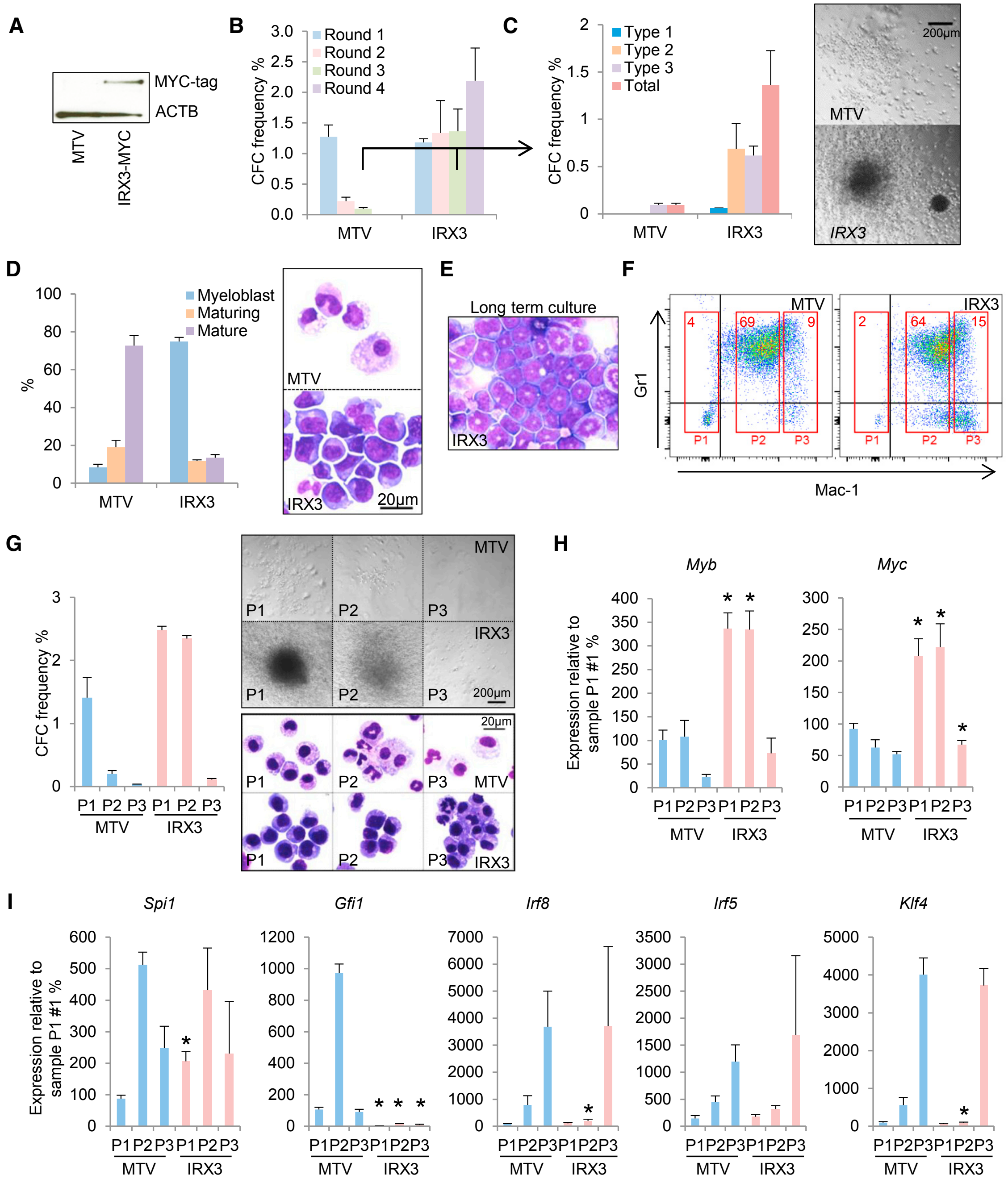

Figure 2. IRX3 Enhances Clonogenic Potential in Normal HSPCs

(A) MYC-tagged IRX3 expressed in murine KIT+ BM HSPCs 72 hr after retroviral infection. MTV, empty vector.

(B) Mean + SEM colony-forming cell (CFC) frequencies during serial replating (n = 3).

(C) Bar chart (left) shows mean + SEM colony types in round 3 of culture (n = 3). Type 1, tightly packed colonies, contain blast cells only; type 2, contain mixed population of blasts and mature cells; type 3, contain mature cells only. Image (right) shows representative colonies.

(D) Bar chart (left) shows mean + SEM percentage of the indicated cell types in cytospin preparations after 7 days of culture of retrovirally infected murine KIT+ BM cells in liquid conditions supporting myeloid development (n = 3). Representative images (right) are shown.

(E) Representative image of *IRX3*-expressing mouse BM cells after 5 weeks in liquid culture.

(F) Representative flow cytometry plots for the indicated cell surface markers after 7 days in liquid culture; red boxes indicate cell sorting gates. Numbers within boxes indicate percentage of cells with the indicated phenotype.

(legend continued on next page)

IRX3 Promotes Serial Replating of Normal BM Stem and Progenitor Cells

To evaluate whether misexpressed *IRX3* has functional consequences, we performed expression experiments in murine KIT^+ BM HSPCs (Figure 2A). Serial replating assays in conditions supporting myeloid lineage differentiation demonstrated that in comparison with control cells, $IRX3^+$ cells exhibited enhanced clonogenic activity (Figures 2A and 2B). In the third round of culture, control cells exclusively formed colonies with type 3 morphology (i.e., diffuse colonies containing mature macrophages), whereas $IRX3^+$ cells in addition formed type 1 (i.e., tightly packed colonies containing blast cells) and type 2 colonies (i.e., mixed colonies containing blast cells and mature cells) (Figure 2C). Reflecting these observations, $IRX3^+$ BM HSPCs cultured in liquid conditions (with interleukin-3 [IL-3], IL-6, granulocyte-macrophage colony-stimulating factor [GM-CSF], and stem cell factor [SCF]) for 7 days following retroviral transduction exhibited significantly impaired morphologic differentiation in comparison with control cells (Figure 2D) and could readily be grown on for at least 5 weeks (Figure 2E). The great majority of day 7 $IRX3^+$ and control cells in liquid culture exhibited a $Mac1^+Gr1^+$ immunophenotype, although there was a modest but significant reduction in the percentage of $Gr1^+$ cells in the $IRX3^+$ condition (Figure S1G). Flow-sorting analyses confirmed, as expected, that the clonogenic activity of control day 7 cells was near exclusively confined to cells with a $Mac\text{-}1^{neg}$ immunophenotype. In contrast, $IRX3^+$ cells with a $Mac\text{-}1^{intermediate}$ immunophenotype also exhibited strong clonogenic activity (Figures 2F and 2G). qPCR analysis of genes associated with self-renewal in myeloid leukemia (Somervaille et al., 2009) demonstrated significant upregulation of *Myc* and *Myb* in $IRX3^+$ populations with significant clonogenic potential (P1 and P2). Furthermore, there was significantly reduced expression in the aberrantly clonogenic $IRX3^+$ P2 population of transcription factor genes such as *Gfi1*, *Irf8*, and *Klf4*, which are associated with myelomonocytic lineage differentiation (Figure 2I). Thus, expression of *IRX3* in normal BM HSPCs in conditions supporting myeloid lineage differentiation confers a morphologic and functional differentiation block resulting in sustained clonogenic activity, elevated expression of self-renewal genes *Myc* and *Myb*, and reduced expression of myelomonocytic differentiation genes *Gfi1*, *Irf8*, and *Klf4*.

IRX3 Cooperates with HOXA9 to Enhance Differentiation Block in AML

Given the strong association of *IRX3* and *HOXA9* expression in human AML, we next evaluated the consequences of *IRX3* co-expression in *Hoxa9*-expressing murine BM HSPCs. Cells were infected in pairwise combinations with retroviral vectors expressing *Hoxa9*, *IRX3*, or a control vector (to generate *Hoxa9/IRX3* and *Hoxa9*/MTV cells, respectively) and serially replated in semisolid culture. Consistent with *IRX3* co-expression conferring an enhanced differentiation block to *Hoxa9*-immortalized HSPCs, by the fourth round of culture, *Hoxa9/IRX3* cells formed significantly more colonies than *Hoxa9*/MTV cells (Figure 3A), and these were significantly more likely to exhibit type 1 blast-like morphology (Figure 3B). Of note, *Hoxa9/IRX3* type 1 colonies were on average 25% smaller in cross-sectional area than *Hoxa9*/MTV type 1 colonies (Figure 3C); this was explained by there being a significantly reduced proportion of *Hoxa9/IRX3* cells in the SG_2M phase of the cell cycle (Figure S1H). The immunophenotype of *Hoxa9/IRX3* and *Hoxa9*/MTV cells was similar (Figure S1I).

Transplantation of *Hoxa9*-expressing murine BM HSPCs into irradiated syngeneic recipients results in short latency AMLs, which exhibit significant myelomonocytic maturation (Kroon et al., 1998; Somerville et al., 2015). To determine whether co-expression of IRX3 influences leukemia cell differentiation *in vivo*, we transplanted *Hoxa9/IRX3* and *Hoxa9*/MTV double-transduced BM HSPCs into irradiated congenic recipients. Consistent with the cell-cycle status of *in vitro* transformed *Hoxa9/IRX3* cells, analysis of blood at 4, 8, and 12 weeks post-transplantation demonstrated reduced donor:recipient chimerism in blood in *Hoxa9/IRX3* versus *Hoxa9*/MTV recipients (Figure 3D), and *Hoxa9/IRX3* recipient mice exhibited delayed onset of donor-derived AML in comparison with *Hoxa9*/MTV recipients (median 125 versus 270 days) (Figure 3E). Mice from both cohorts presented with substantially elevated blood leucocyte counts (Figure S2A), hepatosplenomegaly (Figure S2B), and effacement of BM due to infiltration by leukemia cells (Figures S2C–S2F).

In keeping with a model whereby *IRX3* misexpression *in vivo* blocks myeloid lineage differentiation, evaluation of the lineage composition of donor-derived cells in blood prior to development of AML demonstrated a significant proportional reduction in myeloid and a proportional increase in B-lineage differentiation at 4 and 12 weeks post-transplantation in *Hoxa9/IRX3* versus *Hoxa9*/MTV recipients (Figures 3F and S3A). Once mice developed full-fledged AML, this was further confirmed by analysis of blood smear and BM cytospin morphology: *Hoxa9/IRX3* recipients developed leukemias exhibiting significantly greater differentiation block in comparison with *Hoxa9*/MTV controls, as demonstrated by the proportion of blast cells being on average twice as high in the former versus the latter and the proportion of more differentiated leukemia cells being approximately half as much (Figures 3G and 3H). Flow cytometry analysis of leukemic BM cells confirmed donor origin and myeloid lineage (Figures 3I, 3J, and S3B) and also revealed a distinctive immunophenotype: *Hoxa9/IRX3* AMLs expressed significantly lower levels of both CD45.1 (Figures 3I and S3C) and the monocyte/macrophage differentiation marker F4/80 versus *Hoxa9*/MTV controls. There were also significantly fewer $Mac1^+Gr1^-$ leukemia cells in *Hoxa9/IRX3* recipients and, on average, double the percentage of cells positive for the stem and progenitor

(G) Bar chart (left) shows mean + SEM CFC frequencies for the indicated flow-sorted cell populations enumerated after 5 days (n = 3). Images (right) show representative colonies (top) and cytospins (bottom).
(H and I) Bar charts show mean + SEM relative transcript expression for the indicated genes in the indicated cell populations: (H) *Myb* and *Myc*; (I) *Spi1*, *Gfi1*, *Irf8*, *Irf5*, and *Klf4*. $^*p \leq 0.05$ for the indicated $IRX3^+$ population versus the equivalent control population (unpaired t test).
See also Figure S1.

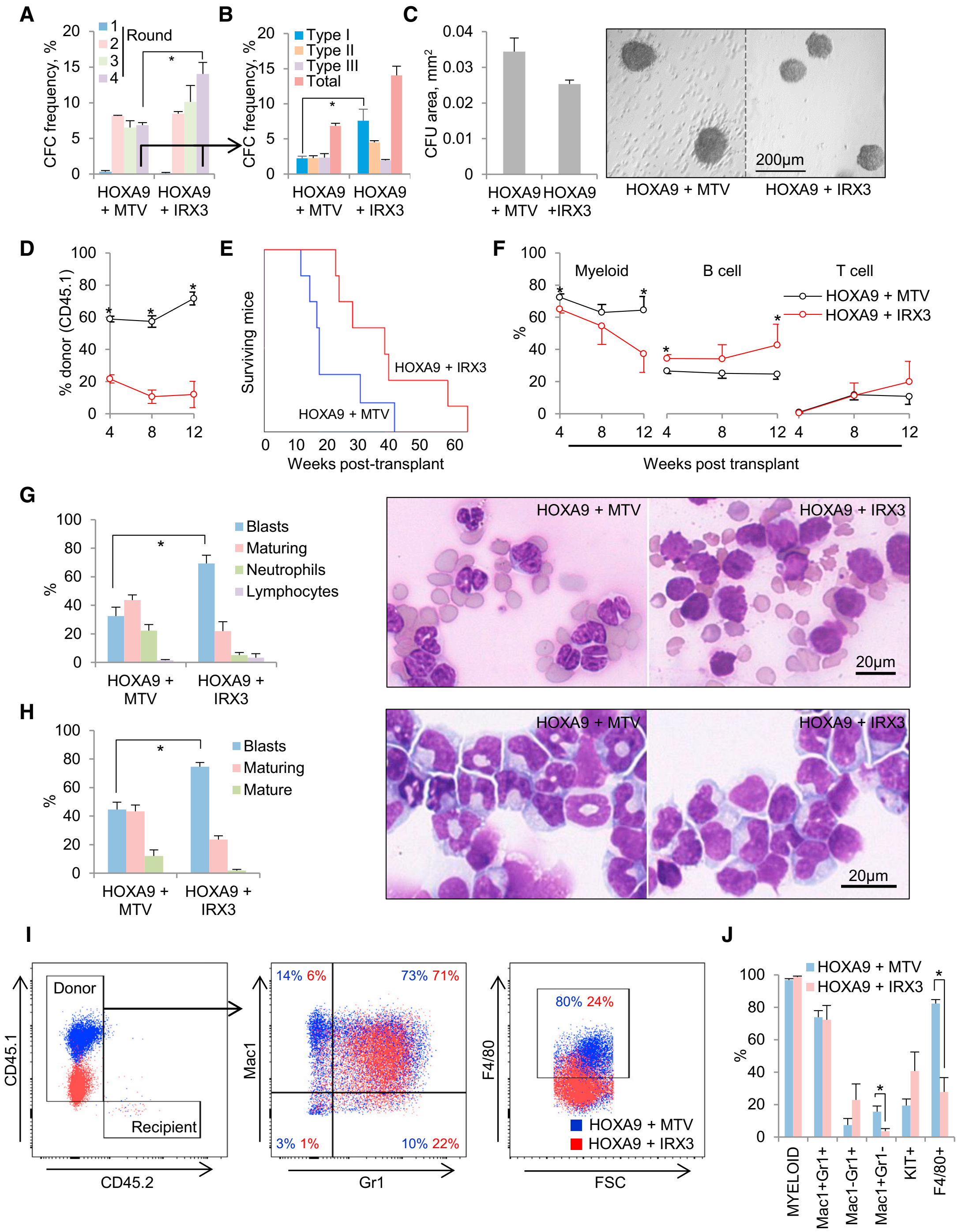

Figure 3. IRX3 Expression Confers an Enhanced Myeloid Differentiation Block in *Hoxa9*$^{+}$ AML

(A) Bar chart shows mean + SEM colony-forming cell (CFC) frequencies during serial replating of murine KIT^{+} BM cells co-transduced with the indicated retroviral or control (MTV) expression vectors (n = 3). *p < 0.05 by unpaired t test.

(B) Bar chart shows mean + SEM colony types in round 4 (n = 3). *p < 0.05 by unpaired t test.

(C) Bar chart (left) shows mean + SEM area of type 1 colonies (n = 26–40) from (B) and representative images (right).

(D–J) Murine CD45.1^{+} KIT^{+} BM cells were infected in pairwise combination with retroviral vectors, and 96 hr later 10^{6} drug-resistant cells were transplanted into CD45.2^{+} irradiated congenic recipients. (D) Line graph shows mean + SEM percentage donor-derived CD45.1^{+} cells in blood at the indicated times post-transplantation. (E) Survival curves of transplanted mice (n = 7 per cohort). (F) Line graphs show the mean + SEM percentage contribution of donor-derived cells in blood to the indicated lineages at the indicated times post-transplantation. (G) Bar chart (left) shows mean + SEM percentage leucocyte type in blood at death, as determined by morphologic analysis of blood smears (n = 4 or 5 per cohort). Representative images (right) are shown. (H) Bar chart (left) shows mean + SEM

(legend continued on next page)

marker KIT (Figure 3J), although in view of heterogeneous levels of expression, this difference did not reach statistical significance. *Hoxa9/IRX3* AML cells induced leukemias in secondarily transplanted recipients (Figure S3D), and high-level *IRX3* expression was readily detected in *Hoxa9/IRX3* AML BM cells (Figure S3E). Interestingly, in contrast to observations *in vitro*, cell-cycle analysis of leukemia cells from BM and spleen of leukemic mice demonstrated no difference in the fraction of cycling cells between the cohorts (Figure S3F), although in both cases, the SG2M fraction was substantially lower in the *in vivo* setting in comparison with the *in vitro* setting (i.e., 10%–20% versus 40%–50%).

Altogether our observations demonstrate that co-expression of *IRX3* modulates the phenotypic consequences of *Hoxa9* expression both *in vitro* and *in vivo*, conferring a significantly enhanced myeloid lineage differentiation block.

Functional Contribution of IRX3 to Differentiation Block in AML Cells

To further confirm that tissue-inappropriate expression of *IRX3* contributes functionally to the differentiation block in AML, we performed knockdown (KD) experiments in human THP1 AML cells, which exhibit the highest levels of *IRX3* expression among AML cell lines we tested (Figure S4A). *IRX3* KD led to loss of clonogenic potential (Figures 4A–4C), which was due to induction of differentiation, as evidenced by upregulation of the myeloid differentiation markers CD11b, CD14, and CD86 (Figures 4D and 4E) and morphologic analysis (Figure 4F). The proportion of apoptotic cells was unaffected (Figure S4B). To confirm that the observed phenotype was an on-target consequence of *IRX3* KD, similar experiments were performed using KD construct #2, which targets the 3′UTR region of *IRX3*, in a THP1 line engineered to express an *IRX3* cDNA that lacks it. Sustained *IRX3* expression in THP1 cells infected with lentiviral vectors expressing KD construct #2 was confirmed (Figures 4G and 4H), as was rescue of the differentiation phenotype (i.e., loss of clonogenic potential and upregulation of the differentiation marker CD86) (Figures 4I, S4C, and S4D). Similar experiments in murine MLL-AF9 AML cells, which also express *Irx3*, gave similar results: KD cells exhibited loss of clonogenic potential and terminal monocyte/macrophage lineage differentiation (Figures S4E–S4G). *IRX3* KD in a range of additional human AML cell lines also led to loss of clonogenic potential in many cases, but not all (Figure S4H). Of note, KD construct #2 had no effect on the clonogenic potential of K562 cells, which do not express *IRX3* (Figure S4A). Furthermore, the formation of colony-forming unit granulocyte monocyte (CFU-GM) and colony-forming unit monocyte/macrophage (CFU-M) from normal human $CD34^+$ cells (which express *IRX3* at very low levels) was unaffected by *IRX3* KD construct #2 (Figures S4I and S4J), although, for unclear reasons, there was a reduction in the formation of erythroid burst-forming units.

Thus, in keeping with *in vitro* and *in vivo* murine experiments, misexpressed *IRX3* contributes to the differentiation block in AML cells and sustains clonogenic potential.

IRX3 Expression in AML Represses a Myelomonocytic Differentiation Program

To evaluate the consequences for the transcriptome of co-expression of *IRX3* with *Hoxa9*, we performed RNA sequencing of flow-sorted KIT^+Gr1^+ leukemia cells recovered from the BM of sick mice, three from each cohort. Cells with this immunophenotype are enriched for leukemia-initiating activity in *Hoxa9/Meis1* murine leukemias (Gibbs et al., 2012). Analysis of 8,954 protein-coding genes that passed threshold criteria (i.e., expression >0.5 reads per kilobase per million mapped reads [RPKM] in at least one sample) revealed that 197 were upregulated and 403 downregulated by at least 2-fold in *Hoxa9/IRX3* versus *Hoxa9/MTV* leukemias (Figure 5A). Gene Ontology analysis revealed significant enrichment within the *Hoxa9/IRX3* downregulated gene set of biological process terms such as "immune response," "leucocyte activation," and "defense response" (Table S5), suggesting that IRX3-repressed genes are associated with mature myeloid cells. At similar levels of significance, there were no enriched terms among the *Hoxa9/IRX3* upregulated gene set. Gene set enrichment analysis (GSEA) confirmed that co-expression of *IRX3* with *Hoxa9* led to repression of a mature myeloid lineage program in KIT^+Gr1^+ leukemia cells, in keeping with morphologic analysis (Figures 3G and 3H). Among genes downregulated in *Hoxa9/IRX3* versus *Hoxa9/MTV* AML cells, there was significant enrichment for genes highly expressed in both mature monocytes and mature neutrophils (i.e., myelomonocytic genes) (Figure 5B; Tables S6 and S7).

GSEA also revealed significant overlap with expression patterns observed in murine models of leukemia associated with high-level expression of *Hoxa9* and *Meis1*. Mixed-lineage leukemia (MLL) fusion oncogenes require *Hoxa9* and *Meis1* to properly transform HSPCs and to establish a leukemia cell hierarchy. We observed that in *Hoxa9/IRX3* versus *Hoxa9/MTV* AML cells, there was significant enrichment of expression of the transcriptional sub-program that contributes to blocked differentiation and enhanced self-renewal of leukemia stem cells (LSCs) in murine MLL leukemias (LSC maintenance program; Somervaille et al., 2009) (Figure 5C; Tables S6 and S7). Likewise, the set of genes anti-correlated with the LSC maintenance program (i.e., associated with differentiated leukemia cells, downstream of the LSC) was strongly repressed in *Hoxa9/IRX3* versus *Hoxa9/MTV* AML cells (Figure 5C; Tables S6 and S7). Similar positive and negative enrichments were observed for gene sets derived from transformed progenitor cells with sustained (HOXA9_MEIS1 POSITIVELY REGULATED) or withdrawn (HOXA9_MEIS1 NEGATIVELY REGULATED) *Hoxa9/Meis1* dual expression (Hess et al., 2006) (Figure S5A). All together, these analyses demonstrate that co-expression of *IRX3* with *Hoxa9* enhances expression of genes previously associated with LSC

percentage cell type in BM at death (n = 5 per cohort). Representative images (left) are shown. (I) Representative flow cytometry plots from (H). (J) Bar chart shows the mean + SEM percentage of donor-derived cells positive for the indicated cell surface markers in BM of leukemic mice, as determined by flow cytometry. *p < 0.05 by unpaired t test.
See also Figures S1–S3.

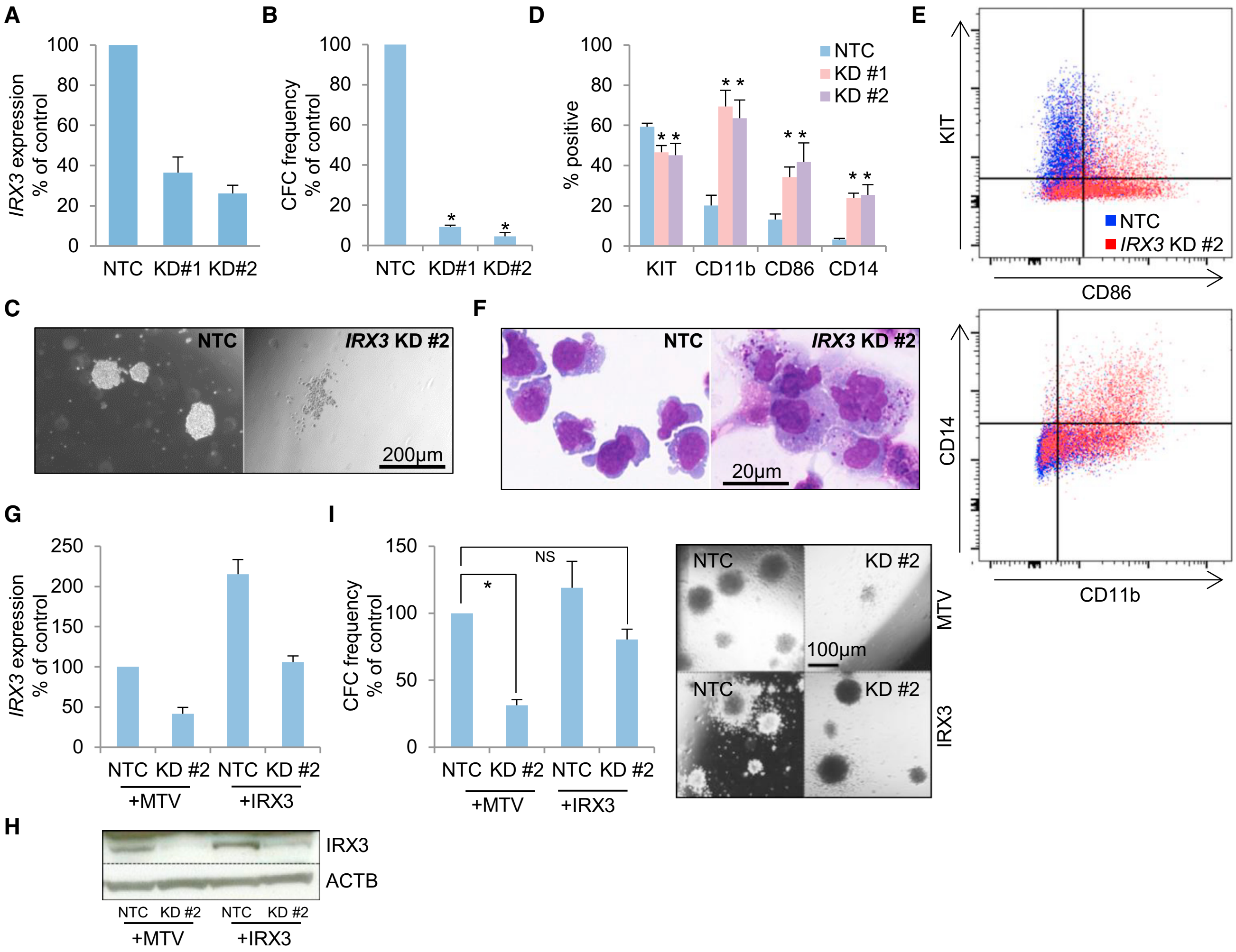

Figure 4. IRX3 Sustains the Differentiation Block and Clonogenic Potential of AML Cells

Human THP1 AML cells were infected with lentiviral vectors targeting *IRX3* for KD or a non-targeting control vector (NTC).

(A) Bar chart shows mean + SEM relative transcript expression in *IRX3* KD versus control cells (n = 3) after 48 hr.

(B) Bar chart shows the mean + SEM colony-forming cell (CFC) frequencies of KD cells relative to control cells enumerated after 10 days in semi-solid culture (n = 4).

(C) Representative images of colonies of cells from (B).

(D) Bar chart shows mean + SEM percentage of cells positive for the indicated cell surface markers, as determined by flow cytometry analysis 6 days following initiation of KD (n = 4). *$p \leq 0.05$ using one-way ANOVA with Fisher's least significant difference post hoc analysis for KD conditions versus NTC.

(E) Representative flow cytometry plots from (D).

(F) Representative images of cytospins of cells from (D).

(G) Bar chart shows mean + SEM relative transcript expression in THP1 AML cells expressing either *IRX3* or a control retroviral vector (MTV) in *IRX3* KD#2 cells relative to control cells (n = 3).

(H) Western blot shows expression of the indicated proteins in the indicated conditions.

(I) Bar chart (left) shows mean + SEM CFC frequencies of THP1 AML cells expressing either *IRX3* or a control retroviral vector (MTV) in *IRX3* KD#2 cells relative to control cells. Colonies were enumerated after 10 days in semi-solid culture (n = 4). Image (right) shows representative colonies. *$p \leq 0.05$ using one-way ANOVA with Fisher's least significant difference post hoc analysis for the indicated comparison. NS, not significant.

See also Figure S4.

self-renewal and represses expression of a terminal myeloid lineage differentiation program.

We next evaluated whether a signature of IRX3 transcriptional activity could be detected in human AML. We ranked protein-coding genes in *IRX3*^{high} versus IRX3^{low} human AMLs (Wouters et al., 2009) using a signal-to-noise ranking metric (Table S6) and performed GSEA using the set of genes repressed by IRX3 in murine *Hoxa9/IRX3* AML cells (Table S5). There was highly significant negative enrichment of IRX3-repressed genes in human *IRX3*^{high} versus *IRX3*^{low} human AMLs whether all AMLs were considered or just those expressing high levels of *HOXA9* (Figure 5D). Indeed, in leading-edge analyses, there was a highly

OPEN ACCESS
CellPress

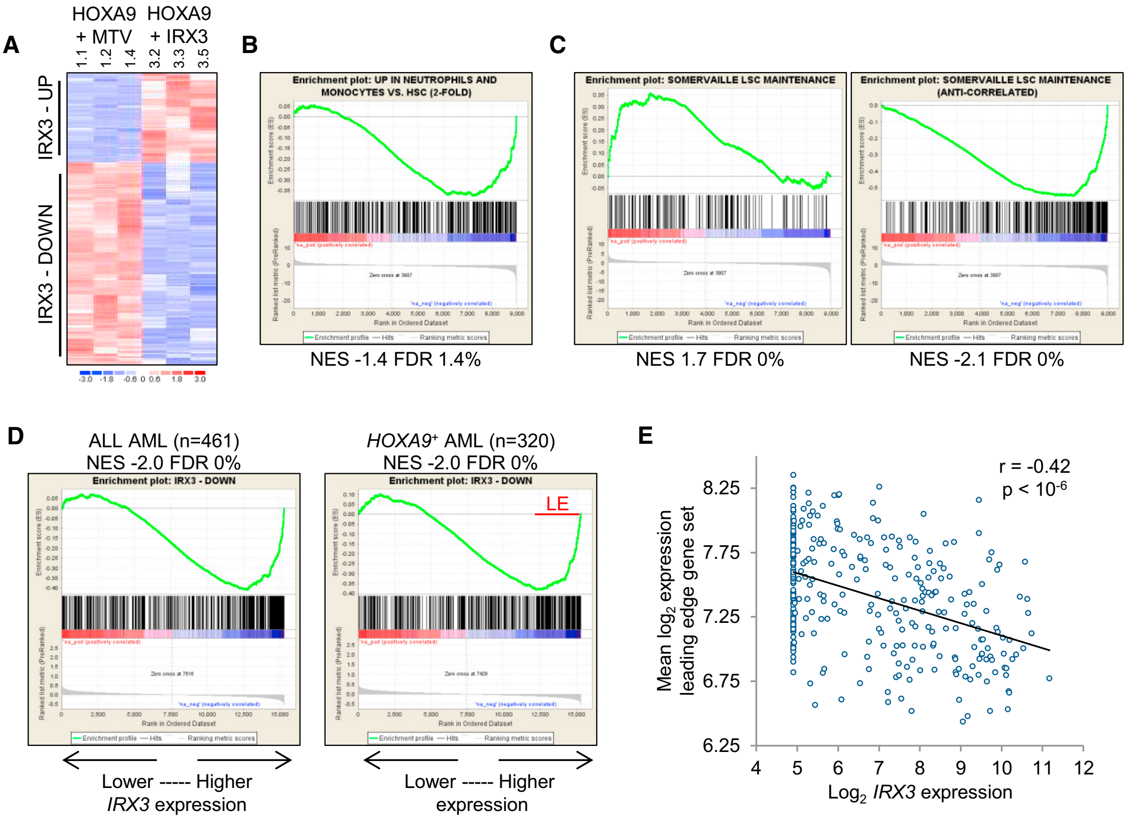

F

FAB category	*IRX3*high (n=131)	*IRX3*low (n= 145)	p value
M0	2 (2%)	9 (6%)	NS
M1	40 (31%)	27 (19%)	0.02
M2	36 (27%)	22 (15%)	0.02
M1 & M2	76 (58%)	49 (34%)	<0.0001
M4	13 (10%)	33 (23%)	0.006
M5	33 (25%)	49 (34%)	NS
M4 & M5	46 (35%)	82 (57%)	0.0004
M6	3 (2%)	2 (1%)	NS
M7	0 (0%)	0 (0%)	NS
Unknown	4 (3%)	3 (2%)	NS

Figure 5. IRX3 Represses a Myelomonocytic Differentiation Program in Murine and Human AML

(A) Heatmap shows differentially expressed protein-coding genes ($p < 0.05$, unpaired t test, >2-fold).

(B–D) GSEA plots show enriched expression of (B) mature myelomonocytic genes, (C) LSC maintenance signature genes, and (D) IRX3-repressed genes in (B and C) *Hoxa9*/MTV versus *Hoxa9*/*IRX3* AML cells or (D) primary human AMLs ranked by *IRX3* expression, respectively.

(E) Scatterplot shows expression of *IRX3* in primary human *HOXA9*$^+$ AML samples versus mean $\log_2$ expression level for the leading-edge gene set shown in (D).

(F) Morphologic classification of *IRX3*^{high} versus *IRX3*^{low} primary *HOXA9*$^+$ AML samples (Wouters et al., 2009). p values were calculated using Fisher's exact test.

See also Figure S5 and Tables S5, S6, and S7.

significant association of higher *IRX3* expression in human *HOXA9*$^+$ AML with greater repression of IRX3-repressed genes identified in murine leukemias (Figure 5E; Table S7). Remarkably, when the morphologic classification of *HOXA9*$^+$ AMLs was considered, among cases with high *IRX3* expression, there were significantly fewer AMLs exhibiting myelomonocytic differentiation (i.e., French-American-British [FAB] M4 subtype) and significantly more AMLs exhibiting minimal differentiation (i.e., FAB M1 subtype) or maturation toward the granulocytic lineage (i.e., FAB M2 subtype) (Figure 5F). Thus, in primary human AML, as in murine AML, misexpression of *IRX3* contributes functionally to blockade of myelomonocytic lineage differentiation.

IRX3 and FOXC1 Differentially Repress Expression of Myelomonocytic Transcription Factors

We previously reported that the mesenchymal transcription factor gene *FOXC1* is also frequently misexpressed in human AML to confer, in particular, a monocytic lineage differentiation block (Somerville et al., 2015). With regard to the morphologic classification of human AML, *FOXC1*-expressing leukemias

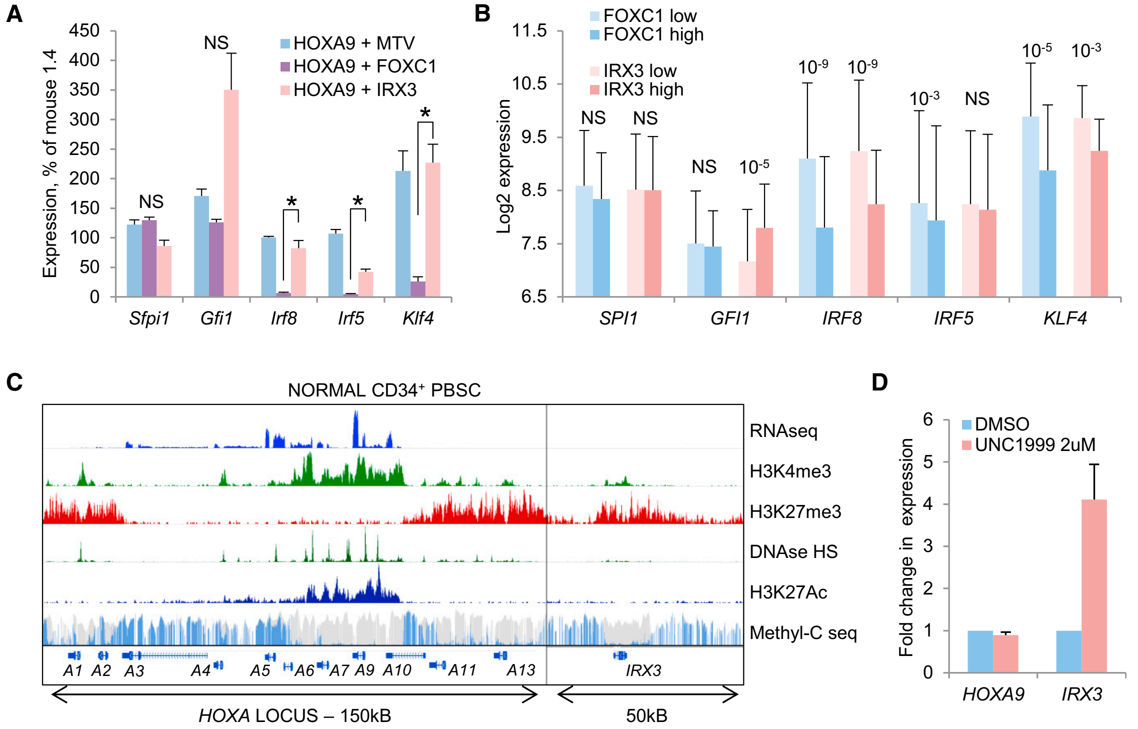

Figure 6. IRX3 Represses Myelomonocytic Genes in Human AML

(A) Bar chart shows mean + SEM relative expression of the indicated genes in flow-sorted murine KIT$^+$Gr1$^+$ BM AML cells (MTV, control, empty vector) (n = 5 or 6 per cohort). *p $\leq$ 0.05 using one-way ANOVA with Fisher's least significant difference post hoc analysis for the indicated comparison. NS, not significant.

(B) Bar chart shows mean + SD $\log_2$ array expression values for the indicated genes in *FOXC1*^{high} (n = 95) versus *FOXC1*^{low} (n = 175) and *IRX3*^{high} (n = 133) versus *IRX3*^{low} (n = 158) human *HOXA9*$^+$ AML cases (Wouters et al., 2009). p values (unpaired t test) are shown where significant. NS, not significant.

(C) Image shows high-throughput sequencing tracks from the ENCODE consortium for the *HOXA* locus and *IRX3*.

(D) Bar chart shows mean + SEM relative expression of *IRX3* and *HOXA9* in normal human CD34$^+$ cells from separate donors following 5 days of treatment with UNC1999 or DMSO in serum-free liquid culture (n = 4).

See also Figure S5.

were significantly less likely to exhibit monocytic lineage differentiation (i.e., FAB-M5) and significantly more likely to exhibit granulocytic lineage differentiation (i.e., FAB M2) (Somerville et al., 2015). The presence of high *FOXC1* expression in AML was also associated with inferior outcome, in contrast to the presence of high *IRX3* expression (Figure S5B). Murine *Hoxa9/FOXC1* leukemias also exhibited shortened latency versus *Hoxa9/MTV* leukemias (Somerville et al., 2015), in contrast to the latencies observed for *Hoxa9/IRX3* leukemias (Figure 3E).

To evaluate the consequences of misexpressed *IRX3* and *FOXC1* on expression levels of transcription factors required for normal myelomonocytic lineage differentiation, and to determine why the phenotypic consequences of *IRX3* and *FOXC1* misexpression differed one from another, we performed qPCR using murine leukemia samples and analyzed expression levels in published human AML datasets. In flow-sorted murine BM KIT$^+$Gr1$^+$ AML cells, expression levels of transcription factor genes such as *Irf8*, *Irf5*, and *Klf4* (which promote monocytic lineage differentiation) were significantly lower in *Hoxa9/FOXC1* AMLs in comparison with *Hoxa9/IRX3* and *Hoxa9*/MTV AMLs (Figure 6A). There was no significant difference in expression levels of the myeloid lineage master regulator *Sfpi1*. In the case of *Gfi1* (which promotes granulocytic lineage differentiation), there was a variable increase in expression in *Hoxa9/IRX3* AMLs compared with the other subtypes, although this did not achieve statistical significance.

In primary human AML samples, a very similar pattern was observed. In comparison with *FOXC1*^{low} or *IRX3*^{low} cases, *FOXC1*^{high} or *IRX3*^{high} cases respectively exhibited significantly lower level expression of *IRF8* and *KLF4*, and for both genes, the proportionate reduction in expression was greater for *FOXC1*^{high} cases than for *IRX3*^{high} cases (Figure 6B). *IRF5* expression was significantly lower in *FOXC1*^{high} versus *FOXC1*^{low} cases and no different between the *IRX3* groups, whereas *GFI1* expression was significantly higher in *IRX3*^{high} versus *IRX3*^{low} cases and no different between the *FOXC1* groups. Expression levels of *SPI1* did not differ. Similar analyses using a separate, smaller dataset from TCGA also gave similar results (Figure S5C), in particular with regard to expression levels of *GFI1* and *IRF8*. Together these data demonstrate that although *IRX3* and *FOXC1* misexpression contribute to the differentiation block observed in human AML, they repress myelomonocytic lineage transcription factor genes in a distinctive

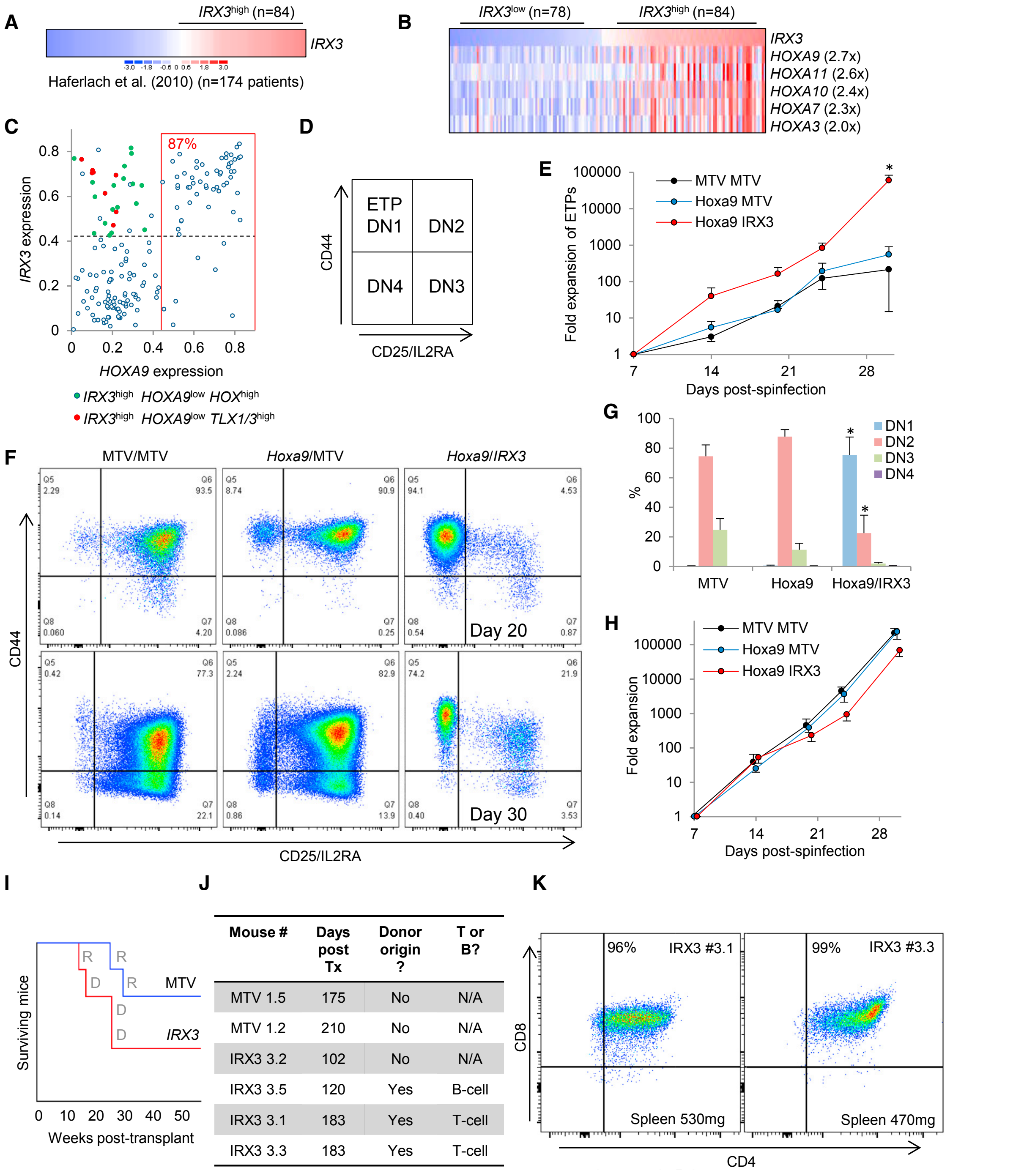

Mouse #	Days post Tx	Donor origin ?	T or B?
MTV 1.5	175	No	N/A
MTV 1.2	210	No	N/A
IRX3 3.2	102	No	N/A
IRX3 3.5	120	Yes	B-cell
IRX3 3.1	183	Yes	T-cell
IRX3 3.3	183	Yes	T-cell

Figure 7. IRX3 Misexpression in Lymphoblastic Leukemia

(A) *IRX3* expression in bulk human T-ALL samples.

(B) Heatmap shows *IRX3*^{high} versus *IRX3*^{low} T-ALL cases and the most differentially expressed transcription factor genes with mean fold change.

(C) *IRX3* versus *HOXA9* expression in primary human T-ALL (Haferlach et al., 2010). Percentage in red text indicates the proportion of *HOXA9*^{high} samples (red box) with high *IRX3* expression (above the dotted gray line).

(D) Immunophenotypic definition of early T cell progenitor double-negative (DN) populations.

(E) Mean + SEM fold expansion in OP9 DL1 stromal culture of ETP/DN1 cells over input number on day 7 (n = 4–6). MTV, empty vector.

(F) Representative flow cytometry plots.

(G) Immunophenotypic developmental stage of Lin^{neg} BM HSPCs expressing the indicated gene combinations cultured for 30 days on OP9 DL1 stroma (mean + SEM percentage, n = 6).

(legend continued on next page)

manner: FOXC1 represses monocytic lineage genes more profoundly than does IRX3, whereas the opposite is the case for GFI1.

Polycomb Activity Sustains IRX3 Repression in Normal Human CD34$^+$ Stem and Progenitor Cells

IRX3 is only minimally expressed in normal CD34$^+$ cells, and its genetic locus is marked by high levels of H3K27 trimethylation (Zhou et al., 2011) (Figure 6C), suggesting that its relative transcriptional silence is maintained by Polycomb-mediated repression. To address this question, normal human CD34$^+$ HSPCs from multiple donors were treated *in vitro* for 5 days with a dual EZH1 and EZH2 inhibitor (UNC1999; Konze et al., 2013). We observed a significant increase in *IRX3* expression but no change in *HOXA9* expression (Figure 6D); the *HOXA9* locus is not marked by H3K27 trimethylation in normal CD34$^+$ HSPCs (Figure 6C). Thus, the Polycomb complex contributes to continued repression of *IRX3* in normal HSPCs.

IRX3 Is Frequently Co-expressed with HOX Genes in Human Acute Lymphoblastic Leukemia

To provide a more comprehensive evaluation of the role of *IRX3* in human acute leukemia, we analyzed published expression datasets from patients with acute lymphoblastic leukemia (ALL). In a cohort of T-ALL patients (Microarray Innovations in Leukemia [MILE] study; Haferlach et al., 2010), *IRX3* transcripts were detected at high level (i.e., with a probeset [229638_at] value of $\geq$ 0.42, approximating to a value among the top 25% of array probeset values) in 84 of 174 cases (48%) (Figure 7A). To identify transcription factor genes concordantly expressed with *IRX3* in T-ALL, we compared *IRX3*^{high} (array signal intensity > 0.42, n = 84) with *IRX3*^{low} (array signal intensity < 0.3, n = 78) cases and observed that only *HOXA* genes were upregulated by greater than a mean 2-fold change in array signal intensity (and with $p < 10^{-5}$, by unpaired t test) (Figure 7B). Of the 84 *IRX3*^{high} cases, 52 (62%) expressed *HOXA9* at high level, 18 (21%) were *HOXA9*^{low} but expressed one or more alternate HOX genes, and 8 (14%) were HOX^{low} but instead expressed the homeodomain transcription factors *TLX1* or *TLX3* (Figure 7C). Of the *HOXA9*^{high} T-ALL cases (i.e., 60 of 174), 52 of 60 (87%) were *IRX3*^{high} (Figure 7C). High HOXA expression is a feature of human T-ALL associated with *CALM-AF10* fusions, *SET-NUP214* fusions, *MLL* gene rearrangements, or inv(7) or t(7;7) translocations (resulting in apposition of the HOXA locus to *TCR-β* regulatory elements; Soulier et al., 2005; Van Vlierberghe et al., 2008).

In B-acute lymphoblastic leukemia (B-ALL), high *IRX3* expression was detected in 116 of 563 cases (21%) in the MILE cohort (Figure S6A). There was a particular association of high *IRX3* expression with the presence of an *MLL* gene rearrangement (31 of 70 cases [44%]), or the presence of t(12;21), the cytogenetic hallmark of the *ETV6-RUNX1* fusion (44 of 58 cases [76%]) (Figure S6B). To identify transcription factor genes exhibiting concordant expression with *IRX3* in B-ALL, we compared *IRX3*^{high} (array signal intensity > 0.42, n = 116) with *IRX3*^{low} (array signal intensity < 0.3, n = 398) B-ALLs. Only *HOXA9*, *MEIS1*, and *SOX11* were upregulated by greater than a mean 2-fold change in array signal intensity (and with $p < 10^{-5}$, by unpaired t test) in *IRX3*^{high} B-ALLs (Figure S6B). Of the 57 *MLL* rearranged *HOXA9*^{high} cases, 27 (47%) were *IRX3*^{high} (Figure S6C). *IRX3*^{high} cases with a t(12;21) did not express HOX genes at high level (Figure S6B). Together these data demonstrate that *IRX3*, which is expressed at very low levels in normal hematopoiesis, is frequently highly expressed in human ALL.

IRX3 Impedes Phenotypic Differentiation of T Cell Precursors and Induces Lymphoid Leukemias

To evaluate the functional consequences of *IRX3* expression in early stage lymphoid development, Lin^{neg} murine BM HSPCs were double-transduced with pairwise combinations of *IRX3*, *Hoxa9*, or control retroviral vectors and, following drug selection, co-cultured on stromal layers. Where BM cells were co-cultured on OP9 stroma (which supports B-lineage differentiation), we observed no significant difference in upregulation of the B-lineage markers B220 and CD19 or expansion of cell numbers (Figure S6D). In contrast, where cells were co-cultured on OP9 DL1 stroma (which ectopically expresses the Notch ligand DLL1 and supports T-lineage differentiation), we observed a highly significant block in differentiation of *Hoxa9*/*IRX3* co-expressing cells at the early thymic progenitor (ETP) stage. Differentiating T cell progenitors exhibit sequential expression of CD44 and CD25, which together define developmental stages ETP/DN1 through to DN4 (Figure 7D) (Yui and Rothenberg, 2014). Over the 4 week assay, there was a mean 100-fold greater expansion of cells with an ETP/DN1 immunophenotype in the presence of *Hoxa9*/*IRX3* co-expression, whereas cells expressing *Hoxa9* alone or control cells readily progressed to DN2 and DN3 downstream differentiation stages (Figures 7E–7G). Overall expansion of cell numbers was similar (Figure 7H). Of note, in contrast to BM HSPCs cultured in myeloid conditions (Figure 2), *IRX3*/MTV expressing Lin^{neg} cells failed to expand on OP9 DL1 stroma (n = 4).

In keeping with a role for IRX3 in promoting the development of lymphoid leukemias, we found that irradiated congenic mice transplanted with *IRX3*-expressing KIT$^+$ BM HSPCs developed lymphoid leukemias with incomplete penetrance. In the 12 weeks after transplantation, although there was significantly reduced donor:recipient chimerism in comparison with animals receiving

(H) Mean + SEM fold expansion in OP9 DL1 stromal culture of total cell number over input numbers on day 7 (n = 4–6). For (G) and (H), *$p \leq 0.05$ using one-way ANOVA with Fisher's least significant difference post hoc analysis for *Hoxa9*/*IRX3* versus *Hoxa9* and MTV conditions.
Murine CD45.1$^+$ KIT$^+$ BM cells were infected with *IRX3*-expressing or control retroviral vectors, and 96 hr later, 10^6 drug-resistant cells were transplanted into irradiated CD45.2$^+$ congenic recipients.
(I) Survival curves. D, donor-derived leukemia; R, recipient-derived leukemia.
(J) Survival times and cause of death.
(K) Flow cytometry plots show immunophenotype of CD45.1$^+$ BM cells at death in the indicated mice.
See also Figures S6 and S7.

control cells (Figure S6E) (as observed in mice transplanted with *IRX3*/*Hoxa9*-expressing cells; Figure 3D), there was no significant proportional difference in myeloid, B-lineage, and T-lineage engraftment (Figures S6E and S6F). Three of six *IRX3*-expressing HSPC recipients developed donor-derived lymphoid leukemia (Figures 7I and 7J); in two cases, the leukemia was T-lineage, and mice exhibited splenomegaly and near total BM involvement (Figures 7K and S7A). These cases expressed *Hoxa* genes at comparable levels to KIT^{+} BM HSPCs (Figure S7B). In the third case, although the mouse was found dead and detailed autopsy could not be completed, flow cytometry analysis of blood cells performed 8 days before death revealed a CD45.1^{lo}B220^{+}CD19^{+} population accounting for 92% of donor-derived leucocytes (Figure S7C). This population was not present 4 weeks earlier, suggesting that this mouse died of a B-lineage leukemia. Three other mice died during the 400 day follow-up period, but all succumbed to recipient-derived hematologic malignancies likely induced by irradiation at transplant conditioning (Figures 7I, 7J, and S7A). At experiment termination, there was no evidence of incipient hematologic neoplasms in remaining mice. Together these data demonstrate that *IRX3* expression impedes normal T-progenitor differentiation *in vitro* and induces T-lineage leukemias *in vivo*.

DISCUSSION

Our studies demonstrate that tissue-inappropriate misexpression of *IRX3* is both frequent and functional in human acute leukemias of multiple lineages. The lack of a major role for *IRX3* and its paralog *IRX5* in normal hematopoiesis is emphasized by the observation of minimal or absent expression in human BM cell populations and genetic knockout experiments that demonstrate that *Irx3*$^{-/-}$ and *Irx5*$^{-/-}$ mice are viable and fertile (Smemo et al., 2014). At least in AML, *IRX3* misexpression is found in both bulk and the putative stem cell compartments, suggesting functional contribution throughout the leukemia clone. With regard to co-regulated gene expression within the *IRX3* topologically associated domain, our qPCR and published dataset analyses indicate that the set of *IRX5*-expressing AMLs is a subset of the *IRX3*-expressing cases, with lower expression levels of *IRX5* than *IRX3*.

Why is *IRX3* expressed so extensively in acute leukemia? Our *in vitro* and *in vivo* studies demonstrate that misexpression of *IRX3* contributes to the cardinal pathologic feature of acute leukemia, the differentiation block. In cultures supporting myeloid lineage differentiation, *IRX3* expression alone or in combination with *Hoxa9* enhanced clonogenic potential and impeded differentiation of normal HSPCs. *In vivo*, *IRX3* co-expression with *Hoxa9* dramatically enhanced the degree of differentiation block in murine AMLs, even though the onset of AML was delayed. Critically, the same transcriptional signature of IRX3-mediated repression of myelomonocytic differentiation was readily identified in human AML, confirming that *IRX3* misexpression is both frequent and functional in human acute leukemia. The paradigm that misexpression of *IRX3* confers a differentiation block is further supported by our observation that co-expression of *IRX3* with *Hoxa9* in T-lineage cultures impeded differentiation of ETPs into downstream developmental stages and that *IRX3*-expressing HSPCs generated lymphoid leukemias *in vivo*. The expanded ETP population may serve as a reservoir for acquisition of genetic mutations required for full-fledged leukemia. The observations that HSPCs expressing *IRX3* alone were immortalized in myeloid culture, failed to expand significantly in OP9 stromal culture, but generated *Hoxa*-expressing lymphoid leukemias *in vivo* reflect the importance of the cellular microenvironment in supporting phenotypic outcome. The outcomes also emphasize the importance of the interaction of misexpressed IRX3 with cell type-specific patterns of chromatin accessibility for transcription factor binding. Cells of different lineages, and of different differentiation states, express different repertoires of transcription factor genes; it is likely that IRX3 binds to chromatin and interferes with gene expression in distinct ways in cells of different lineages.

How is *IRX3* gene expression turned on in hematopoietic cells? Although its expression is positively associated with NPM1 and FLT3 mutations in AML, the link is not absolute; for example, more than a third of NPM1 mutant cases do not express *IRX3* (Wouters et al., 2009). The positive association with other genetic lesions that near invariably lead to high-level HOX gene expression (i.e., a t[6;9] or translocations targeting *MLL* at chromosome 11q23) raises the possibility that *IRX3* is positively regulated by HOX transcription factors. In keeping with this, when *HOXA9* or *HOXA10* is expressed in human CD34^{+} HSPCs, *IRX3* is upregulated (Ferrell et al., 2005). Relatedly, in *Xenopus* development, HOXB4 and IRX5 have overlapping patterns of expression, and the latter is a direct target of the former (Theokli et al., 2003). However, high-level HOX gene expression alone in AML is not sufficient to result in *IRX3* expression, because only 40% of *HOXA9*$^{+}$ cases express *IRX3*. This suggests that additional factors act combinatorially to induce *IRX3*. For example, the Wnt signaling pathway, which is active in AML, induces *IRX3* in forebrain development (Braun et al., 2003). In addition to these candidate positive regulators, it seems likely that loss of repressor activity makes a significant contribution. Our observation that the *IRX3* locus is marked by H3K27me3 in normal CD34^{+} HSPCs and that treatment of cells with a dual EZH1 and EZH2 inhibitor led to *IRX3* upregulation demonstrates that its repression in normal hematopoiesis is dependent on Polycomb.

As well as the strong association of *IRX3* with HOX gene expression in acute leukemias of multiple lineages, there was also high-level expression of *IRX3* in ~90% of cases of APML and in ~75% of cases of t(12;21) B-ALL, leukemias that do not express HOX genes. It is possible that *IRX3* is induced as a direct consequence of PML-RARA or ETV6-RUNX1 fusions, respectively, although the close association of co-expressed *SOX11* in the latter case suggests potential collaboration.

The molecular consequences of *IRX3* misexpression in the acute leukemias remain unclear. It is known that TALE family transcription factors such as MEIS1 and PBX can form triple complexes with HOXA9 that bind to PBX-HOXA9 consensus sequences to regulate gene expression (Shen et al., 1999). We speculate that misexpressed IRX3 might alter the function or stability of HOX transcription factor heterotrimeric complexes, perhaps to prevent downregulation of self-renewal genes or upregulation of transcription factors required for terminal

differentiation. Alternatively, it may redirect HOX transcription factors to new binding sites or function on its own to activate or repress key transcription regulators.

Like *IRX3*, the Forkhead transcription factor gene *FOXC1* is also frequently misexpressed in AML, although the phenotypic consequences in mouse models and primary human AMLs are quite distinct. This is likely related to distinct mechanisms of action and sites of genomic binding; for example, Forkhead and Iroquois transcription factors have different consensus binding motifs and will bind different sites in the genome to regulate overlapping but fundamentally distinct gene sets. In particular, FOXC1 seems more effective than IRX3 at suppressing expression of monocytic lineage transcription factor genes such as *IRF8*, *IRF5*, and *KLF4*. In contrast, in comparison with $IRX3^{low}$ AML cases, in $IRX3^{high}$ cases there is increased expression of the granulocytic lineage regulator gene *GFI1*. In some cases both *FOXC1* and *IRX3* are misexpressed, and here the resulting cellular phenotype will represent the integrated consequence of the prevailing nuclear transcription factor milieu.

In summary, we demonstrate that the Iroquois homeodomain transcription factor IRX3 is frequently misexpressed in human acute leukemia to contribute to the differentiation block that is the pathognomonic feature of the disease. Future investigations will identify approaches to target these transcription factors for pro-differentiation therapies to improve patient outcomes.

EXPERIMENTAL PROCEDURES

Human Tissue and Ethical Approval

Normal $CD34^+$ HSPCs surplus to requirements were from patients undergoing autologous transplantation for lymphoma. Their use was authorized by the Salford and Trafford Research Ethics Committee and, for samples collected since 2006, following written informed consent from donors. Normal human BM was collected with informed consent from healthy adult male donors, with the ethical approval of the Yorkshire Independent Research Ethics Committee. Primary human AML samples were from Manchester Cancer Research Centre's Tissue Biobank (approved by the South Manchester Research Ethics Committee). Their use was authorized by the Tissue Biobank's scientific sub-committee, with the informed consent of donors.

Murine Experiments

Experiments using mice (female, aged 6–12 weeks) were approved by Cancer Research UK Manchester Institute's Animal Ethics Committee and performed under a project license issued by the United Kingdom Home Office, in keeping with the Home Office Animal Scientific Procedures Act of 1986. C57BL/6 ($CD45.2^+$) mice were from Envigo. B6.SJL-*Ptprc*^{a}*Pepc*^{b}/BoyJ ($CD45.1^+$) mice were from Jackson Laboratories and bred in house. Details of transplantation procedures are in the Supplemental Information.

Reagents, Cell Culture, and Flow Cytometry

Details are in the Supplemental Information.

RNA Preparation, qPCR, RNA Sequencing, Bioinformatics, and Statistics

Details are in the Supplemental Information.

Statistical Analyses

Statistical analyses were performed using StatsDirect software version 1.9.7 (StatsDirect), Microsoft Excel, or SPSS for Mac version 22 (IBM). Survival curves were generated using Prism software version 6.0 (GraphPad Software).

DATA AND SOFTWARE AVAILABILITY

The accession number for the RNA sequencing data files reported in this paper is GEO: GSE97450.

SUPPLEMENTAL INFORMATION

Supplemental Information includes Supplemental Experimental Procedures, seven figures, and seven tables and can be found with this article online at https://doi.org/10.1016/j.celrep.2017.12.063.

ACKNOWLEDGMENTS

We thank John Weightman, Jeff Barry, Toni Banyard, Abi Johnson, and staff at the Biological Resources Unit for technical assistance; Dan Wiseman for assistance with figures; and Ruud Delwel for sharing the survival data from the Dutch AML cohort. This work was supported by Cancer Research UK (grant C5759/A20971).

AUTHOR CONTRIBUTIONS

T.D.D.S., F.S., J.A.C., E.L.W., G.J.S., and T.C.P.S. performed experiments and analyzed data. K.B., E.T., and R.J.B. generated the tissue array and performed histopathological stains and analyses. C.W. performed bioinformatics analyses. T.D.D.S. and T.C.P.S. wrote the manuscript.

DECLARATION OF INTERESTS

The authors declare no competing interests.

Received: April 16, 2017
Revised: October 22, 2017
Accepted: December 20, 2017
Published: January 16, 2018

REFERENCES

Braun, M.M., Etheridge, A., Bernard, A., Robertson, C.P., and Roelink, H. (2003). Wnt signaling is required at distinct stages of development for the induction of the posterior forebrain. Development *130*, 5579–5587.

Cerami, E., Gao, J., Dogrusoz, U., Gross, B.E., Sumer, S.O., Aksoy, B.A., Jacobsen, A., Byrne, C.J., Heuer, M.L., Larsson, E., et al. (2012). The cBio cancer genomics portal: an open platform for exploring multidimensional cancer genomics data. Cancer Discov. *2*, 401–404.

Claussnitzer, M., Dankel, S.N., Kim, K.H., Quon, G., Meuleman, W., Haugen, C., Glunk, V., Sousa, I.S., Beaudry, J.L., Puviindran, V., et al. (2015). FTO obesity variant circuitry and adipocyte browning in humans. N. Engl. J. Med. *373*, 895–907.

Cohen, D.R., Cheng, C.W., Cheng, S.H., and Hui, C.C. (2000). Expression of two novel mouse Iroquois homeobox genes during neurogenesis. Mech. Dev. *91*, 317–321.

Ferrell, C.M., Dorsam, S.T., Ohta, H., Humphries, R.K., Derynck, M.K., Haqq, C., Largman, C., and Lawrence, H.J. (2005). Activation of stem-cell specific genes by HOXA9 and HOXA10 homeodomain proteins in CD34+ human cord blood cells. Stem Cells *23*, 644–655.

Gaborit, N., Sakuma, R., Wylie, J.N., Kim, K.H., Zhang, S.S., Hui, C.C., and Bruneau, B.G. (2012). Cooperative and antagonistic roles for Irx3 and Irx5 in cardiac morphogenesis and postnatal physiology. Development *139*, 4007–4019.

Gibbs, K.D., Jr., Jager, A., Crespo, O., Goltsev, Y., Trejo, A., Richard, C.E., and Nolan, G.P. (2012). Decoupling of tumor-initiating activity from stable immunophenotype in HoxA9-Meis1-driven AML. Cell Stem Cell *10*, 210–217.

Goardon, N., Marchi, E., Atzberger, A., Quek, L., Schuh, A., Soneji, S., Woll, P., Mead, A., Alford, K.A., Rout, R., et al. (2011). Coexistence of LMPP-like and GMP-like leukemia stem cells in acute myeloid leukemia. Cancer Cell *19*, 138–152.

Haferlach, T., Kohlmann, A., Wieczorek, L., Basso, G., Kronnie, G.T., Béné, M.C., De Vos, J., Hernández, J.M., Hofmann, W.K., Mills, K.I., et al. (2010). Clinical utility of microarray-based gene expression profiling in the diagnosis and subclassification of leukemia: report from the International Microarray Innovations in Leukemia Study Group. J. Clin. Oncol. *28*, 2529–2537.

Hess, J.L., Bittner, C.B., Zeisig, D.T., Bach, C., Fuchs, U., Borkhardt, A., Frampton, J., and Slany, R.K. (2006). c-Myb is an essential downstream target for homeobox-mediated transformation of hematopoietic cells. Blood *108*, 297–304.

Houweling, A.C., Dildrop, R., Peters, T., Mummenhoff, J., Moorman, A.F., Rüther, U., and Christoffels, V.M. (2001). Gene and cluster-specific expression of the Iroquois family members during mouse development. Mech. Dev. *107*, 169–174.

Kikushige, Y., Shima, T., Takayanagi, S., Urata, S., Miyamoto, T., Iwasaki, H., Takenaka, K., Teshima, T., Tanaka, T., Inagaki, Y., and Akashi, K. (2010). TIM-3 is a promising target to selectively kill acute myeloid leukemia stem cells. Cell Stem Cell *7*, 708–717.

Konze, K.D., Ma, A., Li, F., Barsyte-Lovejoy, D., Parton, T., Macnevin, C.J., Liu, F., Gao, C., Huang, X.P., Kuznetsova, E., et al. (2013). An orally bioavailable chemical probe of the Lysine Methyltransferases EZH2 and EZH1. ACS Chem. Biol. *8*, 1324–1334.

Kroon, E., Krosl, J., Thorsteinsdottir, U., Baban, S., Buchberg, A.M., and Sauvageau, G. (1998). Hoxa9 transforms primary bone marrow cells through specific collaboration with Meis1a but not Pbx1b. EMBO J. *17*, 3714–3725.

Ley, T.J., Miller, C., Ding, L., Raphael, B.J., Mungall, A.J., Robertson, A., Hoadley, K., Triche, T.J., Jr., Laird, P.W., Baty, J.D., et al.; Cancer Genome Atlas Research Network (2013). Genomic and epigenomic landscapes of adult de novo acute myeloid leukemia. N. Engl. J. Med. *368*, 2059–2074.

Li, D., Sakuma, R., Vakili, N.A., Mo, R., Puviindran, V., Deimling, S., Zhang, X., Hopyan, S., and Hui, C.C. (2014). Formation of proximal and anterior limb skeleton requires early function of Irx3 and Irx5 and is negatively regulated by Shh signaling. Dev. Cell *29*, 233–240.

Martorell, Ò., Barriga, F.M., Merlos-Suárez, A., Stephan-Otto Attolini, C., Casanova, J., Batlle, E., Sancho, E., and Casali, A. (2014). Iro/IRX transcription factors negatively regulate Dpp/TGF-β pathway activity during intestinal tumorigenesis. EMBO Rep. *15*, 1210–1218.

Mukherjee, K., and Bürglin, T.R. (2007). Comprehensive analysis of animal TALE homeobox genes: new conserved motifs and cases of accelerated evolution. J. Mol. Evol. *65*, 137–153.

Omatsu, Y., Seike, M., Sugiyama, T., Kume, T., and Nagasawa, T. (2014). Foxc1 is a critical regulator of haematopoietic stem/progenitor cell niche formation. Nature *508*, 536–540.

Saito, Y., Kitamura, H., Hijikata, A., Tomizawa-Murasawa, M., Tanaka, S., Takagi, S., Uchida, N., Suzuki, N., Sone, A., Najima, Y., et al. (2010). Identification of therapeutic targets for quiescent, chemotherapy-resistant human leukemia stem cells. Sci. Transl. Med. *2*, 17ra9.

Shen, W.F., Rozenfeld, S., Kwong, A., Köm ves, L.G., Lawrence, H.J., and Largman, C. (1999). HOXA9 forms triple complexes with PBX2 and MEIS1 in myeloid cells. Mol. Cell. Biol. *19*, 3051–3061.

Smemo, S., Tena, J.J., Kim, K.H., Gamazon, E.R., Sakabe, N.J., Gómez-Marín, C., Aneas, I., Credidio, F.L., Sobreira, D.R., Wasserman, N.F., et al. (2014). Obesity-associated variants within FTO form long-range functional connections with IRX3. Nature *507*, 371–375.

Somervaille, T.C., Matheny, C.J., Spencer, G.J., Iwasaki, M., Rinn, J.L., Witten, D.M., Chang, H.Y., Shurtleff, S.A., Downing, J.R., and Cleary, M.L. (2009). Hierarchical maintenance of MLL myeloid leukemia stem cells employs a transcriptional program shared with embryonic rather than adult stem cells. Cell Stem Cell *4*, 129–140.

Somerville, T.D., Wiseman, D.H., Spencer, G.J., Huang, X., Lynch, J.T., Leong, H.S., Williams, E.L., Cheesman, E., and Somervaille, T.C. (2015). Frequent derepression of the mesenchymal transcription factor gene FOXC1 in acute myeloid leukemia. Cancer Cell *28*, 329–342.

Soulier, J., Clappier, E., Cayuela, J.M., Regnault, A., García-Peydró, M., Dombret, H., Baruchel, A., Toribio, M.L., and Sigaux, F. (2005). HOXA genes are included in genetic and biologic networks defining human acute T-cell leukemia (T-ALL). Blood *106*, 274–286.

Theokli, C., Morsi El-Kadi, A.S., and Morgan, R. (2003). TALE class homeodomain gene Irx5 is an immediate downstream target for Hoxb4 transcriptional regulation. Dev. Dyn. *227*, 48–55.

Van Vlierberghe, P., Pieters, R., Beverloo, H.B., and Meijerink, J.P. (2008). Molecular-genetic insights in paediatric T-cell acute lymphoblastic leukaemia. Br. J. Haematol. *143*, 153–168.

Wiseman, D.H., Greystoke, B.F., and Somervaille, T.C. (2014). The variety of leukemic stem cells in myeloid malignancy. Oncogene *33*, 3091–3098.

Wouters, B.J., Löwenberg, B., Erpelinck-Verschueren, C.A., van Putten, W.L., Valk, P.J., and Delwel, R. (2009). Double CEBPA mutations, but not single CEBPA mutations, define a subgroup of acute myeloid leukemia with a distinctive gene expression profile that is uniquely associated with a favorable outcome. Blood *113*, 3088–3091.

Yui, M.A., and Rothenberg, E.V. (2014). Developmental gene networks: a triathlon on the course to T cell identity. Nat. Rev. Immunol. *14*, 529–545.

Zhang, S.S., Kim, K.H., Rosen, A., Smyth, J.W., Sakuma, R., Delgado-Olguín, P., Davis, M., Chi, N.C., Puviindran, V., Gaborit, N., et al. (2011). Iroquois homeobox gene 3 establishes fast conduction in the cardiac His-Purkinje network. Proc. Natl. Acad. Sci. USA *108*, 13576–13581.

Zhou, X., Maricque, B., Xie, M., Li, D., Sundaram, V., Martin, E.A., Koebbe, B.C., Nielsen, C., Hirst, M., Farnham, P., et al. (2011). The Human Epigenome Browser at Washington University. Nat. Methods *8*, 989–990.

Cell Reports, Volume *22*

Supplemental Information

Derepression of the Iroquois Homeodomain Transcription Factor Gene *IRX3* Confers Differentiation Block in Acute Leukemia

Tim D.D. Somerville, Fabrizio Simeoni, John A. Chadwick, Emma L. Williams, Gary J. Spencer, Katalin Boros, Christopher Wirth, Eleni Tholouli, Richard J. Byers, and Tim C.P. Somervaille

Table S1. Karyotype of 29 AML samples analyzed for *IRX3* expression. Related to Figure 1.

Samples in red exhibit high *IRX3* expression (see Figure 1G). PB = peripheral blood blasts; BM = bone marrow blasts; sl = stemline; sdl = sideline; der = derivative; t = translocation; del = deletion; add = additional material of unknown origin; idem = denotes the stemline karyotype in a subclone; inv = inversion; mar = marker chromosome; cp = composite karyotype.

ID	Sample	Disease Status	Gender	Age	Cytogenetics
54	PB	2nd Relapse	Male	75	47,XY,+11[1]/48,sl,+8[7]/49,sdl,+4[2]
77	BM	Diagnosis	Female	67	Normal
79	BM	Diagnosis	Female	65	47,XX,der(3)t(3;9;14;4)(q2?1;q34;q24;q21),der(4)t(3;9;14;4),del(7)(q22q32),add(8)(q24),der(9)del(9)(p2?3)t(3;9;14;4),der(14)t(3;9;14;4),+18,add(19)(q13.3)x2[9]/46,XX[1]
81	PB	1st Relapse	Male	64	Normal
88	BM	Refractory	Female	64	46,XX,del(12)(p1?2p1?3)[18]/46,XX[2]
93	BM	Diagnosis	Male	59	Normal
103	BM	Diagnosis	Male	51	Normal
104	BM	1st Relapse	Female	31	46,XX,t(6;9;11)(p2?1;p22;q23)[6]/45,idem,der(15)t(15;17)(p11.2;q11.2),-17[4]
107	BM	1st Relapse	Female	16	45,XX,del(7)(q11.2q3?2),t(8;21)(q22;q22),der(12)t(12;18)(p11.2;q11.2),-18[10]/46,XX[1]
114	BM	Diagnosis	Male	56	Normal
120	BM	Diagnosis	Female	68	Normal
122	PB	Diagnosis	Male	66	Normal
127	BM	Diagnosis	Female	63	46,XX,inv(16)(p13q22)[8]/46,XX[2]
136	BM	Diagnosis	Female	61	Normal
144	PB	Diagnosis	Male	71	Normal
146	BM	Diagnosis	Male	52	46,Y,?t(X;6)(p22.1;q?25),t(15;17)(q22;q11.2)[9]/46,XY[1]
149	BM	Diagnosis	Female	49	46,XX,t(15;17)(q22;q11.2)[7]/46,sl,-6,add(16)(q12),+mar[3]/46,XX[3]
155	PB	Diagnosis	Male	63	Normal
163	BM	Diagnosis	Male	20	45,X,-Y,t(8;21)(q22;q22)[8]/46,XY[2]
165	BM	Diagnosis	Female	48	46,XX,t(8;22)(p11;q13),del(9)(q13q32)[10]
170	BM	1st Relapse	Male	50	46,XY,add(6)(p22)[8]/46,XY[2]
189	BM	Diagnosis	Female	76	Normal
191	BM	Diagnosis	Female	38	Normal
222	BM	Diagnosis	Female	80	Normal
228	BM	Diagnosis	Male	54	46,XY,t(1;2)(q2?4;p1?3)[9]/46,XY[1]
232	PB	Diagnosis	Female	85	Normal
234	PB	Diagnosis	Male	84	47,XY,+13[3]/93~94,idemx2[cp3]/92~94<4n>,XXYY,+X,der(5)t(1;5)(q21;q31),+13[cp5]/46,XY[4]
251	BM	Diagnosis	Male	16	46,XY,t(6;9)(p22;q34)[9]/46,XY,der(6)t(6;9),der(9)t(6;9)del(9)(q21q34)[2]
259	BM	Diagnosis	Male	75	47,XY,+8[10]

Table S2. H scoring of trephine biopsy material. Related to Figure 1.

H score and karyotype of 58 AML samples and nine normal samples analyzed for IRX3 protein expression by bone marrow trephine biopsy tissue array. del = deletion; der = derivative; mar = marker chromosome; idem = denotes the stemline karyotype in a subclone; cp = composite karyotype; add = additional material of unknown origin; dic = dicentric; t = translocation; inv = inversion.

Sample number	H score	Karyotype of AML cells or indication for bone marrow (non-malignant)
**1**	0	Weight loss of unclear cause
**2**	0	Normal
**3**	0	Wegener's granulomatosis with pyrexia of unknown origin
**4**	0	Pyrexia of unknown origin
**5**	10	Anaemia
**6**	20	Systemic lupus erythematosus
**7**	20	Normal
**8**	30	Pyrexia of unknown origin
**9**	30	Pyrexia of unknown origin
**1**	0	failed
**2**	0	44X,-Y,t(4;15)(q23;q22),-5,del(17)(p12),+mar1/45X,-Y,idem,+mar2
**3**	0	45XX,der(3)t(3;?11)(p21;q13),-11,-17,+mar1,+?mar2[cp14]/46xx[2]
**4**	0	46,XY,del(7)(q22) [7] / 46,XY [14]
**5**	0	46,XX [73] / 92,XXXX [19]
**6**	0	47,XY,+8[3]/46,XY[9]
**7**	0	Normal
**8**	0	46XX,t(6;9)(q23;q34)/46XX
**9**	0	46XY,add(7)(q3?2),t(8;21)(q22;q22)[2]/45,idem,-Y[8]
**10**	0	Normal
**11**	0	46XX,?del(12)(q24)/46XX
**12**	0	Normal
**13**	0	Normal
**14**	0	42-45,XY,add(1)(q21),-3,-5,add(7)(q3?6),del(11)(q13),-13,-16,dic(16;17)(p11;p11),-21,+2-3 mar[cp3]/83-87,idem[cp3]/46,XY
**15**	0	Normal
**16**	0	failed
**17**	0	46XY,inv(9)(p11;q33)inv(12)(p11;q14)/46XY,inv(9)(p11;q13)
**18**	0	92,XXYY,der(2),t(1;2)(q12;q23)/46XY
**19**	0	45XY,-7/46,idem,+9/46XY
**20**	5	Normal
**21**	10	Normal
**22**	10	Normal
**23**	10	Normal
**24**	20	Normal
**25**	20	Normal
**26**	20	Normal
**27**	20	Normal
**28**	20	46XX,?22p+
**29**	30	Normal
**30**	37.5	failed
**31**	40	Normal
**32**	40	Normal
**33**	45	46-48XX,der(3)t(1;3)(p21;q28),del(5)(q13;q33),der(7)?t(7;13)(q22;q13),-13,add(13)(p12),-21,+mar[cp10]
**34**	45	44,XX,der(4)t(4;12)(p16;q13),-5,-7,-9,-12,+mar1,+mar2
**35**	50	failed
**36**	50	47-50,XY,+1,-5,inv(9)(p11q13),+11,add(17)(p1?1),-18,-22,+r1,+r2,+mar[cp8]
**37**	50	Normal
**38**	70	46XX,del(9)(q21q32),inv(16)(p13q22) [8] /idem,+21 [2]
**39**	80	Normal
**40**	80	Normal
**41**	90	failed
**42**	95	Normal
**43**	105	46XX,t(6;9)(p23;q24)[8]/46XX[5]
**44**	105	Normal
**45**	110	Normal
**46**	120	47XY,+8
**47**	120	45XX,-7,tri(21)(q10)[9]/46XX[2]
**48**	140	Normal
**49**	140	Normal
**50**	140	Normal
**51**	170	failed
**52**	200	Normal
**53**	210	Normal
**54**	230	Normal
**55**	250	failed
**56**	260	Normal
**57**	320	Normal
**58**	350	Normal

Table S3. Association of *IRX3* expression with diagnostic and genetic features in AML. Related to Figure 1.

AML cases were divided into those expressing high or low level *IRX3* based on log_2 expression values of <6.1 or >7.1 respectively (probeset 229638_at). Statistical significance for the indicated comparisons was assessed with Fisher's Exact Test, except for age where an unpaired t-test was used. FAB= French-American-British. NS = not significant.

Characteristic		IRX3^{low} (n=271)	*IRX3*^{high} (n=159)	p value
**Gender**		137M:134F	79M:80F	NS
**Age**		44 (range 15-61)	43 (range 16-60)	NS
**Cytogenetic risk**	Good	70	24	NS
	Intermediate	137 (51%)	104 (65%)	0.004
	Poor	59	26	NS
	Unknown	5	5	NS
**Karyotype**	Trisomy 8	10	8	NS
	11q23	1 (0%)	7 (4%)	0.005
	5 or 7(q) loss	25 (9%)	4 (3%)	0.009
	Complex	12	3	NS
	Normal	98 (36%)	78 (49%)	0.01
	Other	34	16	NS
	t(6;9)	1 (0%)	5 (3%)	0.03
	t(8;21)	33 (12%)	1 (1%)	<0.0001
	inv(16)	31 (11%)	2 (1%)	<0.0001
	t(15;17)	3 (1%)	21 (11%)	<0.0001
	abn(3q)	2	0	NS
	minus 9q	4	1	NS
	t(9;22)	1	0	NS
	Unknown	16	17	NS
**Gene mutations**	*CEBPA* double	24 (9%)	0 (0%)	<0.0001
	IDH1	12 (4%)	19 (12%)	0.006
	IDH2	21	15	NS
	FLT3 ITD	52 (19%)	66 (42%)	<0.0001
	FLT3 TKD	25	21	NS
	NRAS	34 (13%)	7 (4%)	0.006
	KRAS	2	2	NS
	NPM1	44 (16%)	80 (50%)	<0.0001
	FLT3 ITD and *NPM1*	21 (8%)	44 (28%)	0.001
**High *MECOM***		24 (9%)	2 (1%)	0.001

Table S4. Association of *IRX3* expression with genetic features in AML. Related to Figure 1.

Expression data from 163 AML cases with exome or whole genome sequencing were analyzed using cBioPortal (Cerami et al., 2012; The Cancer Genome Atlas Research Network, 2013). Cases were divided into $IRX3^{low}$ and $IRX3^{high}$ groups based on log_2 expression values of <6.1 or >7.1 respectively. Statistical significance for the indicated associations was assessed using Fisher's Exact Test.

**Mutated gene**	**$IRX3^{low}$ (n=101)**	**$IRX3^{high}$ (n=49)**	**p value**
DNMT3A	25	14	NS
TET2	9	6	NS
NPM1	16 (16%)	24 (50%)	<0.0001
IDH1	8	6	NS
IDH2	13	2	NS
RUNX1	15 (15%)	1 (2%)	0.02
TP53	12 (12%)	0 (0%)	0.009
WT1	4	5	NS
PTPN11	2	4	NS
KIT	5	0	NS
EZH2	3	0	NS
HNRNPK	1	0	NS
U2AF1	4	1	NS
SMC1A	5	0	NS
SMC3	5	1	NS
BRINP3	3	2	NS
PHF6	2	3	NS
STAG2	2	3	NS
RAD21	2	3	NS
FLT3 ITD	11 (11%)	17 (34%)	0.0008
FLT3 D835X	6	4	NS
FLT3 ITD & *NPM1* mutation	3 (3%)	12 (24%)	0.0001

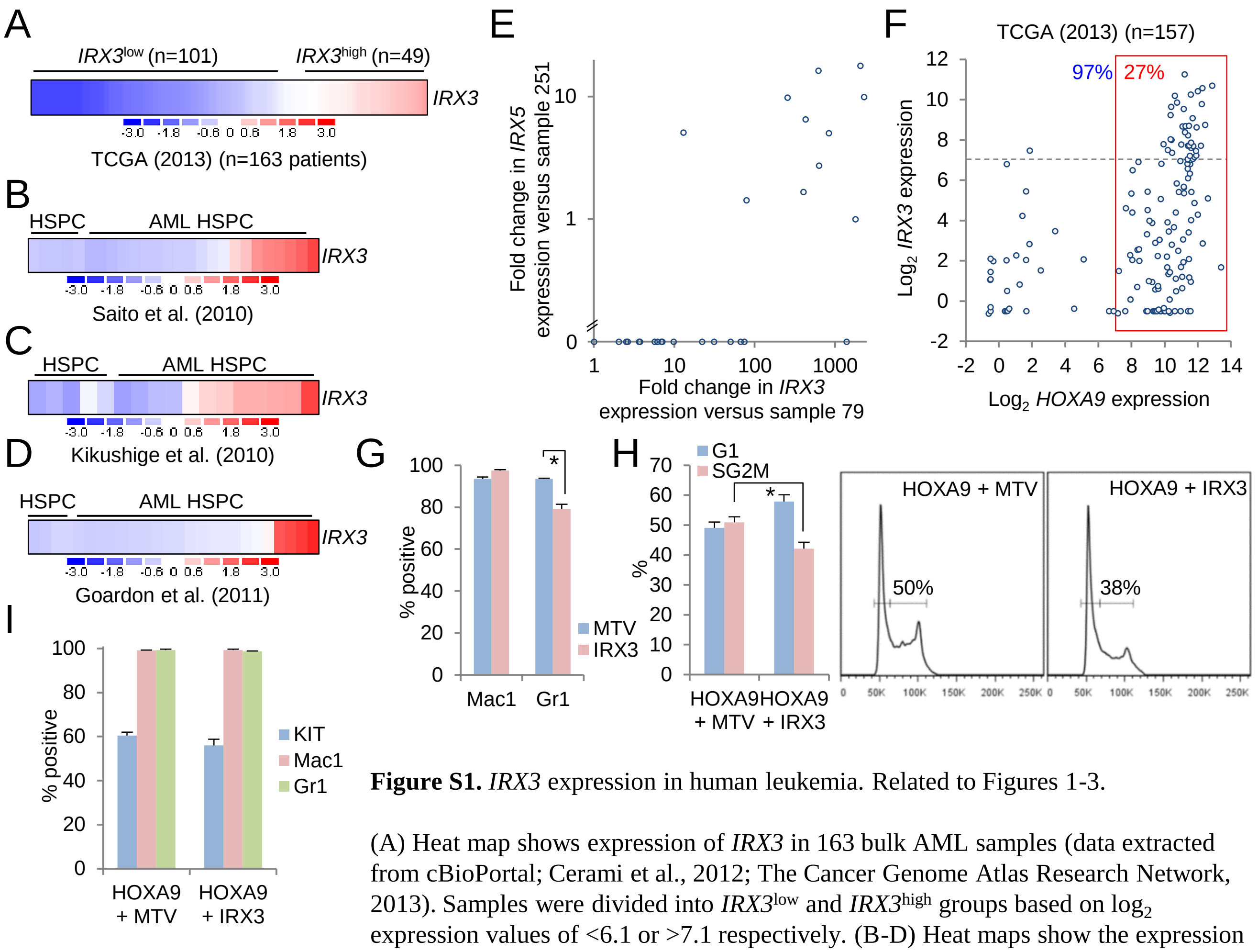

Figure S1. *IRX3* expression in human leukemia. Related to Figures 1-3.

(A) Heat map shows expression of *IRX3* in 163 bulk AML samples (data extracted from cBioPortal; Cerami et al., 2012; The Cancer Genome Atlas Research Network, 2013). Samples were divided into *IRX3*^{low} and *IRX3*^{high} groups based on log_2 expression values of <6.1 or >7.1 respectively. (B-D) Heat maps show the expression of *IRX3* in AML HSPC versus normal adult immunophenotypic BM HSPC. HSPC definitions were (B) $CD34^+CD38^-$ for normal (n=5) and AML cells (n=21); (C) $CD34^+CD38^-Lin^-$ for normal cells (n=5) and $CD34^+CD38^-$ for AML cells (n=12) and (D) $CD34^+CD38^-CD90^+CD45RA^-Lin^-$ for normal cells (n=5) and $CD34^+CD38^+CD123^{+/lo}CD110^-CD45RA^+Lin^-$ (n=21) for AML samples. (E) Scatter plot shows relative expression of *IRX3* versus *IRX5* in primary human bulk AML samples, as determined by quantitative PCR. (F) Scatter plot shows the array expression value of *IRX3* versus *HOXA9* in 157 non-APML primary AML patient samples from the indicated study. Percentage in blue text indicates proportion of *IRX3*^{high} samples exhibiting high *HOXA9* expression; percentage in red text indicates proportion of *HOXA9*^{high} samples exhibiting high *IRX3* expression.

(G) Bar chart shows mean+SEM percentage of cells positive for the indicated cell surface markers as determined by FACS analysis following seven days of liquid culture in conditions favouring myeloid differentiation (n=3).

(H) Bar chart (left panel) shows mean+SEM percentage of cells co-transduced with the indicated retroviral or control (MTV) expression vectors in the indicated stages of the cell cycle (n=3). Representative profiles (right panel) are shown. (I) Bar chart shows mean+SEM percentage of cells positive for the indicated cell surface markers (as determined by flow cytometry) following six days in liquid culture (n=3).

A

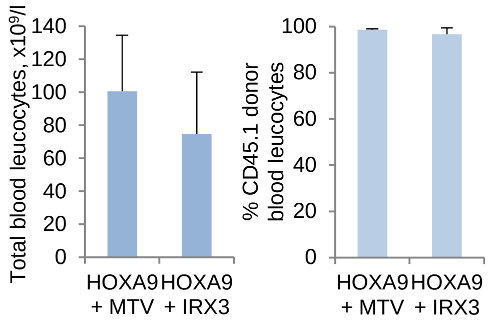

B

Combination	Liver (mg)	Spleen (mg)	Number
Hoxa9/MTV	2313±604	461±66	6
Hoxa9/IRX3	2193±560	447±147	6

C
D
E
F

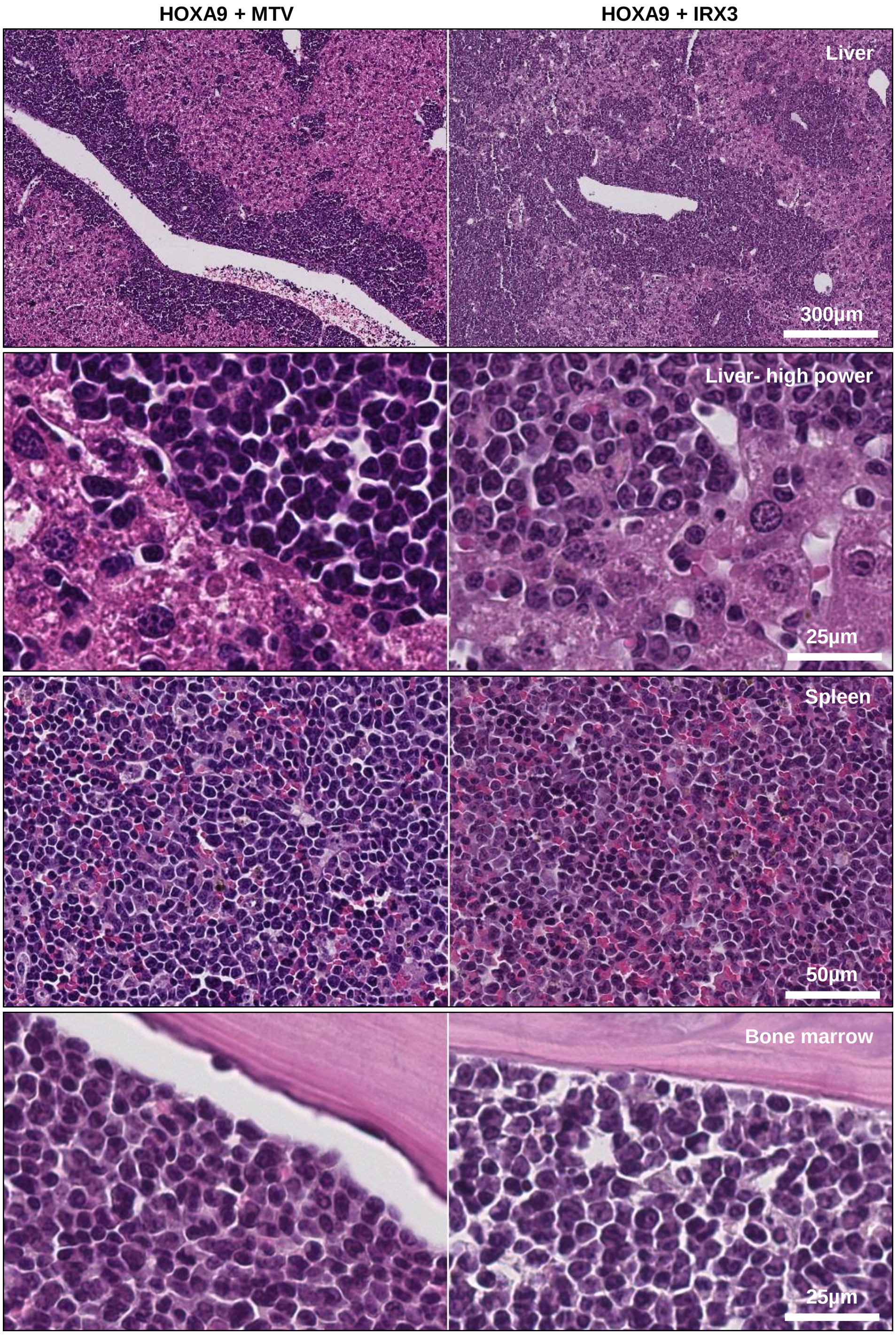

Figure S2. *IRX3* expression enhances the differentiation block in *Hoxa9* induced AML - I. Related to Figure 3.

(A) Bar chart shows mean+SEM total blood leucocyte count at death in the indicated cohorts, as determined by hemocytometer counting (n=4-6 per cohort) (left panel) and the percentage of leucocytes of CD45.1 donor origin. (B) Table shows mean±SD organ masses of leukemic animals at death. Images are representative of (C & D) liver, (E) spleen and (F) BM histology from sick mice.

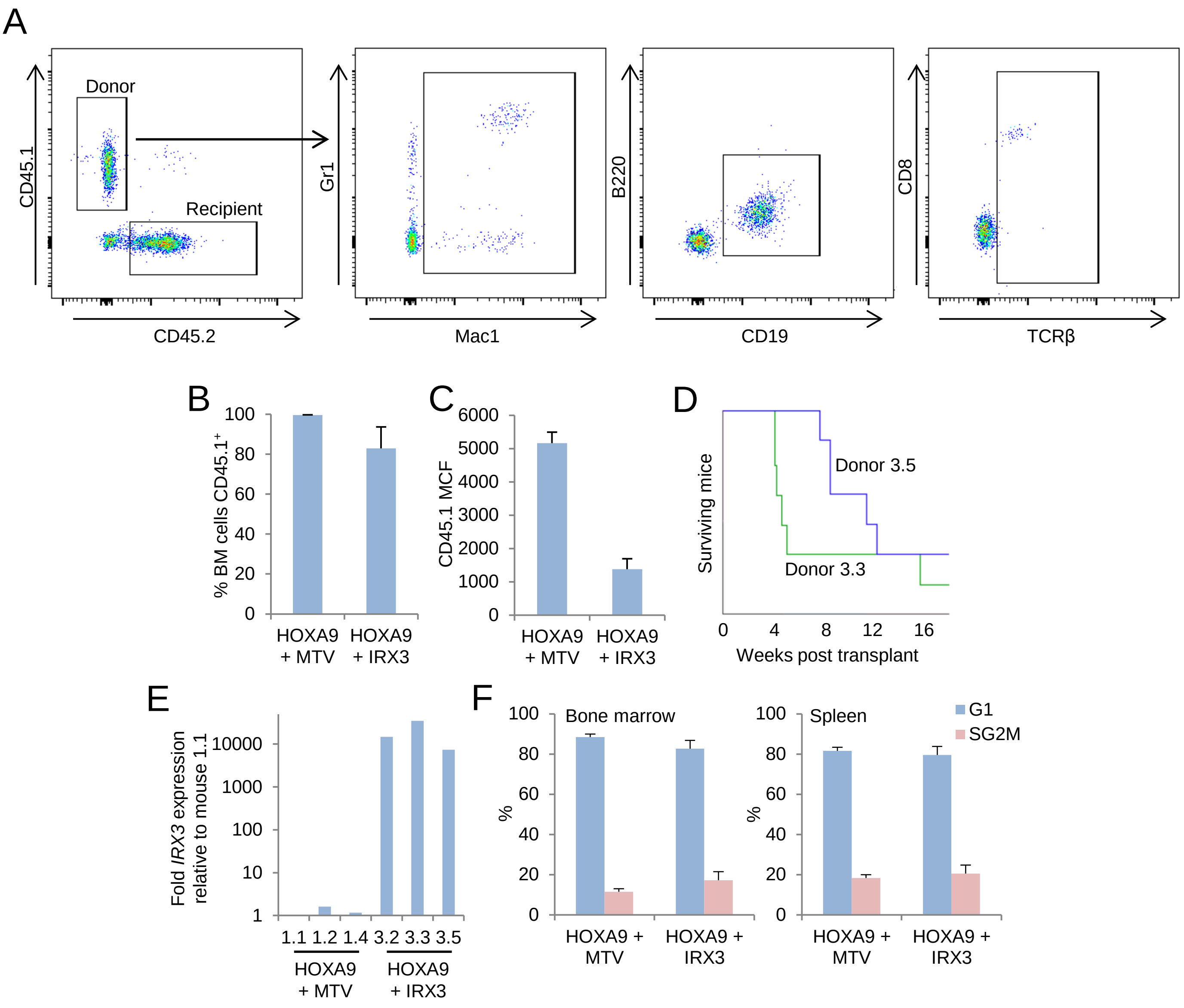

Figure S3. *IRX3* expression enhances the differentiation block in *Hoxa9* induced AML - II. Related to Figure 3.

(A) Representative flow cytometry plots indicate the gating strategy for identifying the percentage of donor-derived CD45.1$^+$ cells, and their lineage contributions, in blood. Bar charts show mean+SEM percentage (B) and mean cellular fluorescence (C) of donor-derived CD45.1$^+$ cells in BM at death (n=6 per cohort). (D) Survival curves of sub-lethally (4.5Gy) irradiated mice secondarily transplanted with 3.3x10^5 (donor 3.3) or 10^6 (donor 3.5) *Hoxa9*/*IRX3* AML cells from the indicated primary recipients. (E) Bar chart shows relative expression of *IRX3* in AML BM cells at the point of death from the indicated recipient mice. (F) Bar charts show mean+SEM percentage of leukemia cells present in the BM (left) and spleen (right) in the indicated phase of the cell cycle at death, as determined by propidium iodide staining and flow cytometry analysis.

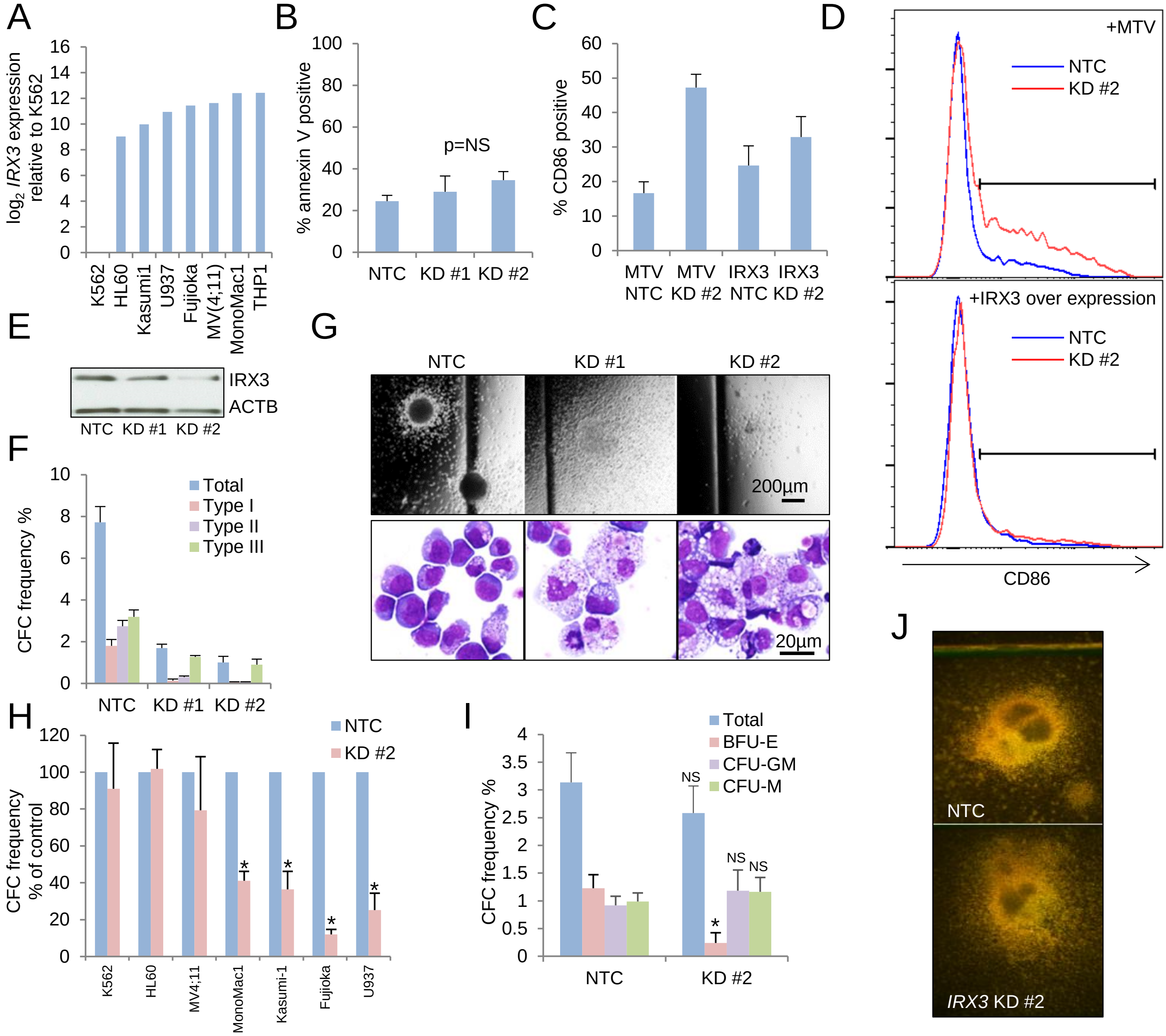

Figure S4. IRX3 sustains the differentiation block and clonogenic potential of AML cells. Related to Figure 4.

(A) Bar chart shows $\log_2$ *IRX3* expression relative to K562 cells in the AML cell lines as determined by qPCR. (B) Bar chart shows mean+SEM apoptotic cells as determined by flow cytometry after seven days of KD (n=3). (C) Bar chart shows mean+SEM percentage of cells positive for the indicated cell surface markers in the indicated conditions, as determined by flow cytometry after six days of KD (n=3). (D) Representative flow cytometry plots from (C). (E-G) Murine MLL-AF9 AML cells were infected with lentiviral vectors targeting *Irx3* for KD (KD#1 and KD#2) or a non-targeting control vector (NTC). (E) Western blot shows expression of the indicated proteins in the indicated conditions. (F) Bar chart shows the mean+SEM colony-forming cell (CFC) frequencies enumerated after seven days (n=3). Type I colonies: contain only blast cells; Type II colonies: mixed blasts and differentiating myeloid cells; Type III colonies: terminally differentiated myeloid cells. (G) Images show representative colonies (top panel) and cytospins (bottom panel) from (F). (H-J) Human AML cell lines (H) or normal human $CD34^{+}$ HSPCs (I & J) were infected with *IRX3* KD#2 vector or a NTC. (H) Bar chart shows mean+SEM CFC frequencies for KD cells relative to controls, enumerated after ten days (n=3). *indicates $p<0.05$, unpaired t-test. (I) Bar chart shows mean+SEM % total and types of colonies formed by normal human $CD34^{+}$ HSPCs infected with the *IRX3* KD#2 vector or a NTC (n=3 separate individuals). Colonies were enumerated after 14 days. (J) Representative images of CFU-GM colonies from (H). BFU-E, burst-forming unit erythroid; CFU-GM, colony-forming unit granulocyte/macrophage; CFU-M, colony-forming unit macrophage.

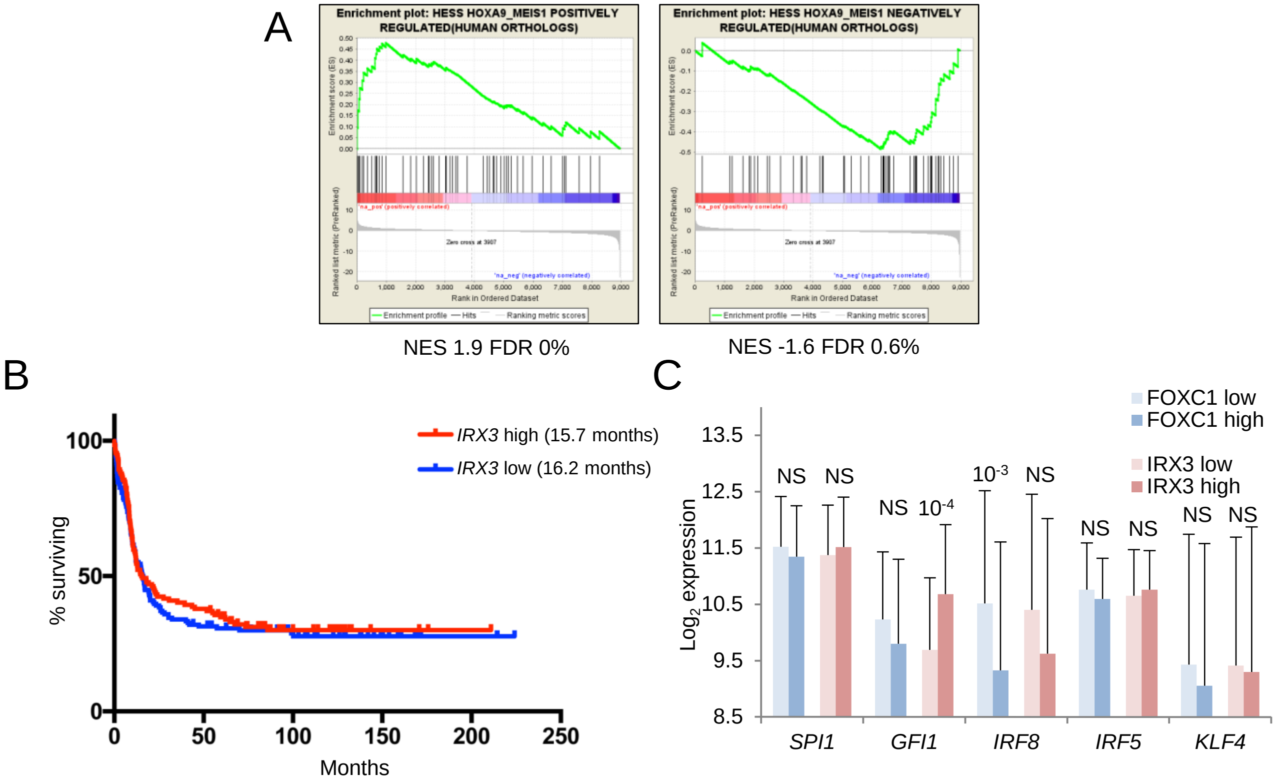

Figure S5. IRX3 represses a myelomonocytic differentiation program in murine and human AML. Related to Figures 5 & 6.

(A) GSEA plots show enriched expression of HOXA9/MEIS1 regulated genes (Hess et al., 2006) in *Hoxa9/IRX3* AMLs versus *Hoxa9/MTV* AMLs.

(B) Survival curves of patients with *IRX3*^{high} versus *IRX3*^{low} AML. (C) Bar chart shows mean+SD $\log_2$ RNAseq expression values for the indicated myelomonocytic transcription factor genes in *HOXA9*$^{+}$ *FOXC1*^{high} (n=45) versus *FOXC1*^{low} (n=69) and *IRX3*^{high} (n=35) versus *IRX3*^{low} (n=81) human AML cases (data extracted from cBioPortal; Cerami et al., 2012; The Cancer Genome Atlas Research Network, 2013). *P* values (unpaired t-test) are shown where significant. NS = not significant.

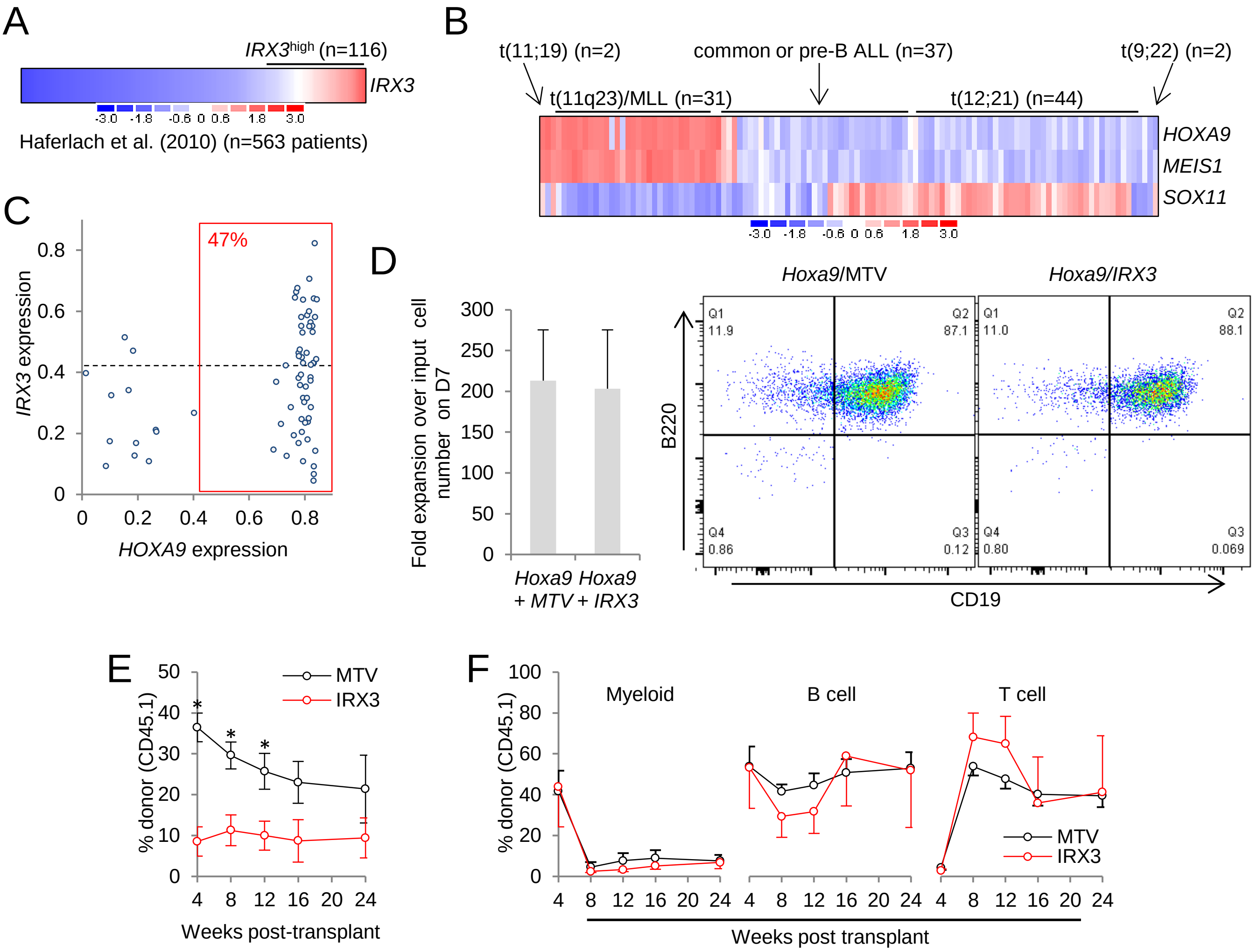

Figure S6. The association of *IRX3* expression with lymphoid leukemia - I. Related to Figure 7.

(A) Heat map shows *IRX3* expression in 563 bulk B-ALL samples (data from Haferlach et al., 2010). The *IRX3*^{high} group was defined by expression values of ≥0.42 (probeset 229638_at). (B) Heat map shows relative expression of the indicated genes in 116 *IRX3*^{high} B-ALL patient samples. (C) Expression values of *IRX3* versus *HOXA9* in primary B-ALL patient samples with an MLL-gene rearrangement (Haferlach et al., 2010). For heat maps, color scale indicates standardized expression level.

(D) Bar chart (left panel) shows mean+SEM fold expansion over input numbers after 21 days of culture on OP9 stroma of Lin^{neg} BM HSPCs expressing the indicated combination of genes following retroviral infection (MTV – empty vector). Right panel shows representative flow cytometry profiles of D21 cells.

(E & F) Murine CD45.1^{+} CD117^{+} BM cells were infected with *IRX3*-expressing or control (MTV) retroviral vectors and 96 hours later 10^6 drug resistant cells were transplanted into irradiated CD45.2^{+} congenic recipients. Line graphs show the mean±SEM percentage (E) total and (F) lineage specific contribution of donor-derived cells in blood at the indicated times post-transplantation.

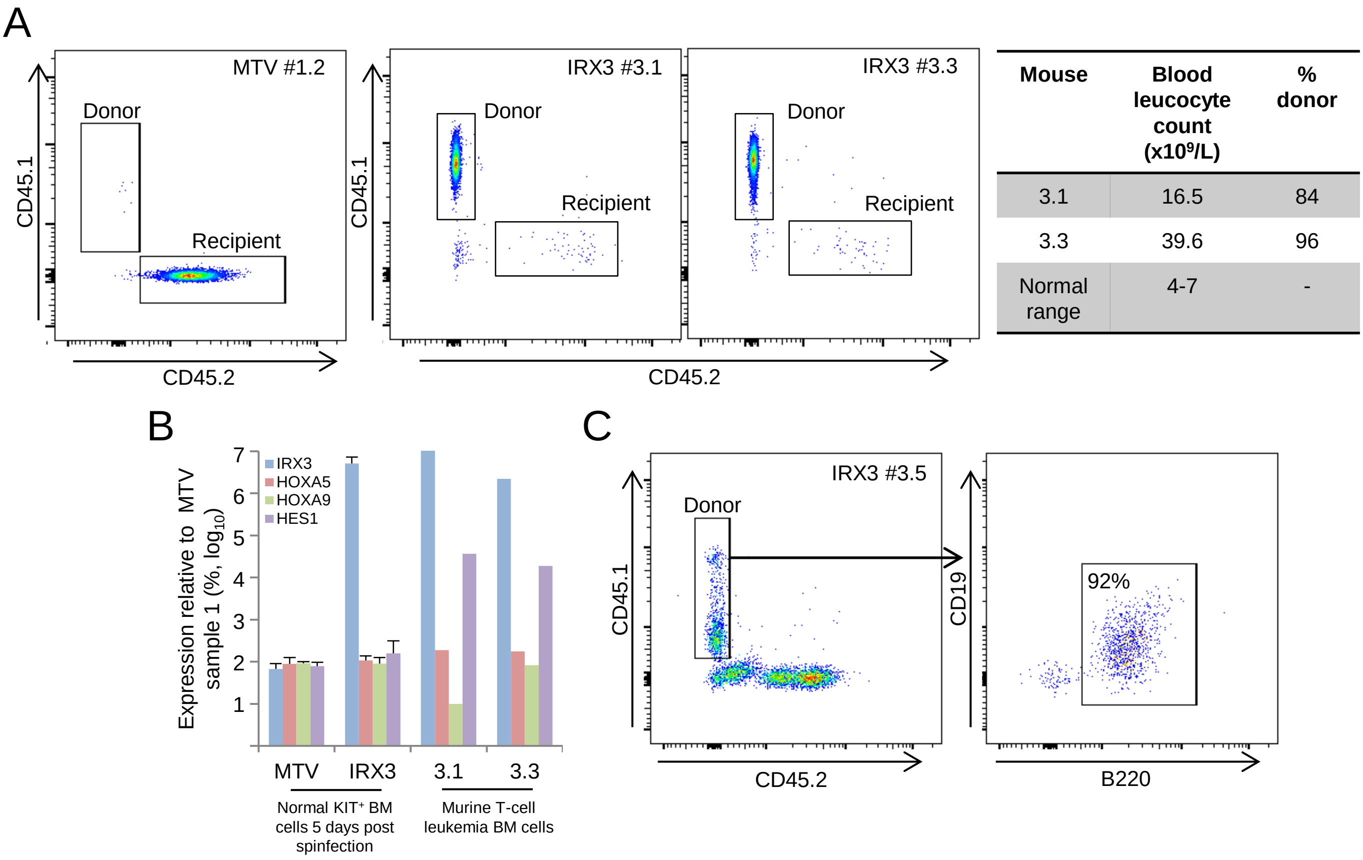

Mouse	Blood leucocyte count ($x10^9$/L)	% donor
3.1	16.5	84
3.3	39.6	96
Normal range	4-7	-

Figure S7. The association of *IRX3* expression with lymphoid leukemia - II. Related to Figure 7.

Murine CD45.1$^+$ CD117$^+$ BM cells were infected with *IRX3*-expressing or control (MTV) retroviral vectors and 96 hours later 10^6 drug resistant cells were transplanted into irradiated CD45.2$^+$ congenic recipients. (A) Representative flow cytometry plots indicate the proportional contribution of donor (CD45.1$^+$) and recipient (CD45.2$^+$) cells to the BM of the indicated mice at death. Table (right panel) shows total white cell counts in blood at death in the indicated mice. (B) Bar chart shows gene expression (as determined by Q-PCR) in the indicated cell populations. For KIT$^+$ BM cells, mean±SEM for three biological replicates is shown. For murine leukemias, the mean of three technical replicates is shown. (C) Flow cytometry plots show immunophenotype of CD45.1$^+$ nucleated cells in blood in the indicated mouse eight days prior to death.

Supplemental experimental procedures

Murine experiments

For transplantation experiments, BM cells were harvested from the long bones of six to 12 week-old female CD45.1$^+$ mice and KIT$^+$ cells recovered with an AutoMACS Pro device (Miltenyi Biotec, Bergisch Gladbach, Germany). Cells were incubated overnight in RPMI with 20% fetal bovine serum (FBS) (R20) and 20ng/ml SCF, 10ng/ml IL6, 10ng/ml GM-CSF and 10ng/ml IL3 (Peprotech, London, UK). Cells were then spinoculated with retroviral supernatant, prepared as described (Harris et al., 2012), and incubated overnight in R20 with growth factors prior to the addition of puromycin (3μg/ml) and/or neomycin (1.5mg/ml). After 96hr of drug selection 1x10^6 viable cells (as determined by Trypan blue dye exclusion) were injected into the tail veins of lethally irradiated (800 cGy) recipient six to 12 week-old female CD45.2$^+$ mice. Mice receiving doubly transduced cells received in addition 2x10^5 CD45.2$^+$ unfractionated BM cells. Tail bleeds were performed monthly and blood donor:recipient chimerism and lineage contributions determined by flow cytometry following ammonium chloride lysis of erythrocytes. Blood leucocytes were counted using a hemocytometer following staining with Türk's solution. Sick mice were euthanized and leukemia cells cryopreserved for later use.

Reagents, plasmids and virus manufacture

Puromycin, neomycin and UNC1999 were from Sigma (St Louis, MO). Polybrene® was from Millipore (Billerica, MA). Retroviral constructs encoding FLAG-Hoxa9 (pMSCVneo and pMSCVpuro) are as described (Somerville et al., 2015). To generate a lentiviral expression vector for *IRX3, IRX3* cDNA (NM_024336.2) was first excised from pOTB7-*IRX3* (Plasmid ID Repository, Harvard Medical School, Boston MA) through triple digest with the *EcoRI*, *XhoI* and *ClaI* restriction enzymes and, using *EcoRI* and *XhoI* restriction sites, sub-cloned into pLentiGS-EGFP (Huang et al., 2014). To generate a retroviral expression vector for *IRX3,* the coding sequence for *IRX3* was PCR amplified from pLentiGS-EGFP-*IRX3* using primers designed to incorporate *EcoRI* and *XhoI* restriction sites, complete with a 3’MYC-tag. Gel purified, amplified DNA fragments were sub-cloned directly into the *EcoRI* and *XhoI* sites of pMSCV(P2)-puro (Somervaille et al., 2009).

Lentiviral vectors for KD experiments were from Sigma: non-targeting control pLKO.1 (SHC002), *IRX3* KD#1 pLKO.1 (TRCN0000016902) and *IRX3* KD#2 pLKO.2 (TRCN0000436197). Lentiviral supernatants were prepared and normal or leukemic human or murine cells infected with viral particles as described (Harris et al., 2012; Somerville et al., 2015).

Cell culture

THP1, HL60 and MonoMac-1 cells were from DMSZ (Braunschweig, Germany). MV(4;11), U937, Fujioka and K562 cells were from Vaskar Saha (Manchester, UK) and Kasumi-1 cells were from Constanze Bonifer (Birmingham, UK). Cell lines were cultured as recommended by DMSZ and their identities verified by STR analysis. Experimentally initiated MLL-AF9 AML cells were generated and cultured as described (Somervaille and Cleary, 2006). Primary normal human CD34$^+$ cells were isolated and cultured as described (Somerville et al., 2015).

For clonogenic assays, human cells were cultured in methylcellulose medium (H4320, Stem Cell Technologies, Vancouver, Canada) and for those involving normal $CD34^+$ cells, the following cytokines were added: 10ng/ml SCF, 10ng/ml IL6, 50ng/ml GM-CSF, 20ng/ml IL3, 10ng/ml IL11, 50ng/ml TPO, 10ng/ml FL, (Peprotech), 50ng/ml G-CSF (Chugai, London, UK) and 4U/ml EPO (Janssen Cilag, Beerse, Belgium). Primary human BM stromal cells from patients with acute promyelocytic leukemia in complete molecular remission were isolated by thawing cryopreserved BM into Minimum Essential Medium-alpha (Sigma) and 15% FBS at a density of 10^6 cells/ml. Three days later non-adherent cells were removed and cells cultured to 80% confluence for three passages prior to RNA extraction. Stromal cells displayed the expected $CD45^{neg}$ immunophenotype demonstrating non-hematopoietic origin.

Clonogenic assays of retrovirally transduced murine KIT^+ BM cells were performed in methylcellulose medium (M3231, Stem Cell Technologies) with 20ng/ml SCF, 10ng/ml IL6, 10ng/ml GM-CSF and 10ng/ml IL3 (Peprotech) and puromycin (3µg/ml) or neomycin (1.5mg/ml) as described (Somerville et al., 2015). For liquid culture of murine myeloid cells, R20 supplemented with the same growth factors and antibiotics (where required) was used.

For stromal B- and T-cell lineage co-cultures, Lin^{neg} BM cells were recovered from C57BL/6 mice using immunomagnetic beads and an AutoMacs Pro device (Miltenyi Biotec) and incubated overnight in R20 with 100 ng/ml SCF, 50 ng/ml TPO and 100 ng/ml FLT3L (Peprotech). Cells were next spinfected with retroviral supernatant and then cultured overnight in the same growth factors. Following 96hr of drug selection with puromycin (3 µg/ml) and neomycin (1.5 mg/ml), cells were washed and seeded onto OP9-GFP (for B lineage differentiation) or OP9-DL1-GFP (for T lineage differentiation) monolayers in 24-well plates in α-MEM medium supplemented with 10% FBS with 5ng/ml FLT3L and 1ng/ml IL7 (Peprotech). OP9 cells were a gift from Georges Lacaud. Co-cultures were harvested by forceful pipetting at the indicated time points (i.e. every 5-7 days) and OP9-GFP/OP9-DL1-GFP clumps cells were eliminated by filtering cells through a 70-µm cell strainer prior to counting, replating and flow cytometric analysis. After Day 12, the IL7 concentration used for OP9-DL1-GFP co-cultures was decreased to 0.25ng/ml.

Flow cytometry, protocols and antibodies

Flow cytometry analyses were performed with a LSR Model II flow cytometer (BD Biosciences, Oxford, UK). Cell sorting experiments were performed with an Influx or FACS Aria II flow cytometer (BD Biosciences). Antibodies for flow cytometry were: (A) human antibodies - KIT-APC (clone 104D2), CD11b-PE (clone ICRF44), CD14-FITC (clone 61D3) and CD86-PerCP-eFluor®710 (clone B7-2); (B) murine antibodies - KIT-APC (clone 2B8), Mac1-PE (clone M1/70), Gr1-PECy7 (clone R86-8C5), F4/80-eFluor®450 (clone BM8), CD45.1-APC (clone A20), CD45.2-PerCPCy5.5 (clone104), CD19-PECy7 (clone eBio1D3), B220-PE (clone RA3-6B2), TCRβ-APCeFluor®780 (clone H57-597), CD3-APC (clone 17A2), CD8a-PE (clone 53-6.7), CD4-eFluor®450 (clone GK1.5), CD44-PECy7 (clone IM7) and CD25-PerCPCy5.5 (clone PC61.5). Antibodies were used at 1/200 dilution except for F4/80-eFluor®450 and TCRβ-APCeFluor®780 (both1/100), and CD8a-PE (1/500). All antibodies were from eBioscience (Hatfield, UK).

Normal human BM populations were isolated as described (Huang et al., 2014; Somerville et al., 2015). Human CD19$^+$ B cells and CD3$^+$CD4$^+$ or CD3$^+$CD8$^+$ T cells were flow sorted from thawed, cryopreserved normal BM samples. Apoptosis was assessed using a BD Pharmingen APC Annexin V kit. Propidium iodide cell cycle analyses were performed as described (Somerville et al., 2015).

Trephine biopsy tissue array immune staining was performed using anti-IRX3 (NBP2-32363, Novus Biologicals, Littleton, CO). Immunoreactivity was semi-quantitatively scored by blind evaluation for intensity (weak, medium or strong) and percentage staining to generate the H-score value.

Antibodies for western blotting were: anti-IRX3 (ab25703, Abcam, Cambridge, UK), anti-MYC-tag (2276, Cell Signaling, Beverley, MA) and anti-ACTB (C4, Millipore). Western blotting was performed as described (Somerville et al., 2015).

RNA preparation and quantitative PCR

RNA was extracted and quantitative PCR performed as described (Somerville et al., 2015). Universal Probe Library System (Roche, Basel, Switzerland) designed primers and probes were as follows:

Gene	Sequence (5'→3')	Probe number
ACTB **F**	ATTGGCAATGAGCGGTTC	11
ACTB **R**	GGATGCCACAGGACTCCAT	
HOXA9 **F**	AAAACAATGCTGAGAATGAGAGC	3
HOXA9 **R**	TATAGGGGCACCGCTTTTT	
IRX3 **F**	AAAAGTTACTCAAGACAGCTT	57
IRX3 **R**	GGATGAGGAGAGAGCCGATA	
SPI1 F	CCACTGGAGGTGTCTGACG	51
SPI1 R	CTGGTACAGGCGGATCTTCT	
IRF5 F	GGCTTCAGGGAGCTTCTCTC	1
IRF5 R	AGGTCTGGCAGGAGCTGTT	
Actb **F**	TGACAGGATGCAGAAGGAGA	106
Actb **R**	CGCTCAGGAGGAGCAATG	
Myc **F**	CCTAGTGCTGCATGAGGAGA	77
Myc **R**	TCCACAGACACCACATCAATT	
Myb **F**	CCTTCTCTCCCTCGCAGTT	11
Myb **R**	TGGAGGGTAAGGTAGGTGCAT	
Spi1 **F**	ATGGAGAAGCTGATGGCTTG	27
Spi1 **R**	GGAACTGGTACAGGCGAATC	
Gfi1 **F**	ATGTGCGGCAAGACCTTC	1
Gfi1 **R**	ACAGTCAAAGCTGCGTTCCT	
Irf8 **F**	CCGGCAAGCAGGATTACA	10
Irf8 **R**	GCTTTGTCTCCCTCTTTAAACTTC	
Irf5 **F**	AACCCACTGATGATTACGTTCTG	101
Irf5 **R**	GCCTGGTAGCATTCTCTGGA	
Klf4 **F**	GCTCCTCTACAGCCGAGAATC	10
Klf4 **R**	ATGTCCGCCAGGTTGAAG	

TaqMan primer/probe assays (Life Technologies, Carlsbad, CA) were used for *KLF4* (Hs00358836_m1), *GFI1* (Hs00382207_m1), *IRX5* (Hs04334749_m1) and *IRF8* (Hs00175238_m1). ΔCt values relative to *ACTB* or *Actb* were assessed using SDS software v2.1 (Applied Biosystems, Foster City, CA).

Bioinformatics and RNA sequencing

Analyses of published microarray and RNA sequencing datasets were performed as described (Somerville et al., 2015). Murine gene symbols were converted to human orthologs as described (Harris et al., 2012). For RNA sequencing analysis of murine leukemia samples, BM from sick mice (three animals per cohort) was thawed, and donor derived ($CD45.1^+$) viable KIT^+Gr1^+ leukemia cells were flow sorted. RNA was extracted and its quality confirmed using an Agilent Bioanalyzer (Agilent Technologies, Santa Clara, CA). Indexed PolyA libraries were prepared using 200ng of total RNA and 14 cycles of amplification with the Agilent SureSelect Strand Specific RNA Library Prep Kit for Illumina Sequencing (Agilent, Santa Clara, CA). Libraries were quantified by quantitative PCR using a Kapa Library Quantification Kit for Illumina sequencing platforms (Roche). Paired-end 75bp sequencing was carried out by clustering 1.8 pM of the pooled libraries on a NextSeq 500 sequencer (Illumina, San Diego, CA). Reads were aligned to the mouse genome (GRCm38) with Bowtie2, version 2.2.1 (Langmead et al., 2012) using default settings. SAMtools (v 0.1.19) was used to filter out unaligned reads, non-primary alignments or alignments with a Phred quality score of <20. There were 18.5-21.8 million input reads per sample and 8.3-10.2 million reads per sample remained after filtering. Reads were mapped to annotated protein coding genes (ENSEMBL v76) using the Annmap database, R and Bioconductor (Gentleman et al., 2004). RPKM (reads per kilobase per million uniquely mapped reads) was computed for each gene.

Supplemental references

Gentleman RC, Carey VJ, Bates DM, Bolstad B, Dettling M, Dudoit S, Ellis B, Gautier L, Ge Y, Gentry J et al. 2004. Bioconductor: open software development for computational biology and bioinformatics. *Genome Biology* **5**: R80.

Harris WJ, Huang X, Lynch JT, Spencer GJ, Hitchin JR, Li Y, Ciceri F, Blaser JG, Greystoke BF, Jordan AM et al. 2012. The histone demethylase KDM1A sustains the oncogenic potential of MLL-AF9 leukemia stem cells. *Cancer Cell* **21**: 473-487.

Huang X, Spencer GJ, Lynch JT, Ciceri F, Somerville TD, Somervaille TC. 2014. Enhancers of Polycomb EPC1 and EPC2 sustain the oncogenic potential of MLL leukemia stem cells. *Leukemia* **28**: 1081-1091.

Langmead B, Salzberg SL. 2012. Fast gapped-read alignment with Bowtie 2. *Nature Methods* **9**: 357-359.
